# Trends in Prices of Popular Brand-Name Prescription Drugs in the United States

**DOI:** 10.1001/jamanetworkopen.2019.4791

**Published:** 2019-05-31

**Authors:** Nathan E. Wineinger, Yunyue Zhang, Eric J. Topol

**Affiliations:** 1 Translational Institute, Scripps Research, La Jolla, California

## Abstract

**Importance:**

High and continually increasing pharmaceutical drug spending is a major health and health policy concern in the United States.

**Objective:**

To demonstrate trends in prices among popular brand-name prescription drugs.

**Design, Setting, and Participants:**

This economic evaluation of drug prices focuses on 49 top-selling brand-name medications in the United States. Pharmacy claims data from January 1, 2012, through December 31, 2017, were obtained from Blue Cross Blue Shield Axis, a database that includes data from more than 35 million individuals with private pharmaceutical insurance. Drugs that exceeded $500 million in US sales or $1 billion in worldwide sales were examined.

**Main Outcomes and Measures:**

The median sum of out-of-pocket and insurance costs paid by patients or insurers for common prescriptions, presented annually and monthly, was the primary outcome.

**Results:**

In total, 132 brand-name prescription drugs were identified in 2017 that met the inclusion criteria. Of this total, the study focused on 49 top-selling drugs that exceeded 100 000 pharmacy claims. Substantial cost increases among these drugs was near universal, with a 76% median cost increase from January 2012 through December 2017, and almost all drugs (48 [98%]) displaying regular annual or biannual price increases. Of the 36 drugs that have been available since 2012, 28 (78%) have seen an increase in insurer and out-of-pocket costs by more than 50%, and 16 (44%) have more than doubled in price. Insulins (ie, Novolog, Humalog, and Lantus) and tumor necrosis factor inhibitors (ie, Humira and Enbrel) demonstrated highly correlated price increases, coinciding with some of the largest growth in drug costs. Relative price changes did not differ between drugs that entered the market in the past 3 to 6 years and those that have been on the market longer (number of drugs, 13 vs 36; median, 29% increase from January 2015 through December 2017; *P* = .81) nor between drugs with or without a Food and Drug Administration–approved therapeutic equivalent (number of drugs, 17 vs 32; median, 79% vs 73%; *P* = .21). Changes in prices paid were highly correlated with third-party estimates of changes in drug net prices (ρ = 0.55; *P* = 3.8 × 10^−5^), suggesting that the current rebate system, which incentivizes high list prices and greater reliance on rebates, increases overall costs.

**Conclusions and Relevance:**

The growth of drug spending in the United States associated with government-protected market exclusivity is likely to continue; greater price transparency is warranted.

## Key Points

**Question:**

What are the prices of top-selling brand-name prescription drugs in the United States, and how have these prices changed in recent years?

**Findings:**

In this economic evaluation of 49 common top-selling brand-name drugs, 78% of the drugs that have been available since 2012 have seen an increase in insurer and out-of-pocket costs by more than 50%, and 44% have more than doubled in price.

**Meaning:**

Prices of brand-name drugs in the United States are likely to continue to increase, which warrants greater price transparency.

## Teaser

This economic evaluation study uses pharmaceutical insurance claims data to report the consistent rise in prices of commonly used prescription medicines in the United States.

## Introduction

Pharmaceutical drug net spending in the United States reached $324 billion in 2017 and is expected to increase 2% to 5% annually over the next 5 years.^1^ Per capita pharmaceutical spending is 54% to 209% higher in the United States than other high-income countries^2^ because of higher prices and widening differential of pay between public and private insurers.^3^ This trend persists despite an increase in the proportion of generic drugs prescribed,^4^ which has lowered spending when generic drugs are available,^5^ and is associated with the high and growing costs of brand-name drugs granted government-protected market exclusivity.^6^ (Throughout the text, we refer to cost as the price paid for purchasing a drug and not as manufacturing cost.)

In the United States, most of the insured population typically only directly pays through a copayment insurance program, and the full drug costs are generally not realized beyond the purchase of shared benefit plans from private insurers (often supplemented by employers) or payment of taxes that fund public insurers. For those among the 12.1% of uninsured or underinsured adults,^7^ out-of-pocket costs can be crippling or catastrophic. As such, the debate surrounding drug costs has expanded beyond the academic and political^8^ realms and even into popular culture.^9^

Data on the costs of drugs remain opaque and generally confusing. List prices are set by pharmaceutical manufacturers and represent the payment shared between the payer (ie, insurer) and the insured (ie, through any out-of-pocket costs when a product is purchased). However, manufacturers may offer rebates to pharmacy benefit managers, who act on behalf of the payer during annual negotiations in exchange for preferred formulary placement. Rebates vary by product, manufacturer, and pharmacy benefit manager. Rebates are returned retrospectively after the point of sale based on volume purchases and therefore cannot be directly linked to an individual purchase.^10^ Information on negotiated rebates is proprietary, with overall estimates of net price at 28% off list prices.^11^ Manufacturers may set list prices independently of pharmacy benefit managers, and they may do so at any time for any number of reasons, ranging from benign rationale (eg, manufacturing cost increase) to allegations of illegal practice.^12,13,14,15^

Although it is generally recognized that list prices for brand-name drugs have risen, a review of trends in recent years is warranted. One explanation of increasing list prices is that greater rebates are being offered, which sufficiently offset increased prices, although evidence suggests that adopting these practices is associated with even higher costs billed to consumers.^16^ The US Department of Health and Human Services recently concluded the current rebate system harms federal health care programs and their beneficiaries.^11^

We mapped out the drug costs of the top-selling brand-name drugs in the United States over a 6-year period from 2012 through 2107. We combined out-of-pocket and insurer-paid costs for common brand-name pharmacy prescriptions from private insurers, and we compared these costs with third-party estimates of net prices to assess the implications of rebates. The results highlight the extent of unimpeded, growing drug costs in the pharmaceutical market.

## Methods

This study was deemed as non–human participant research by the Scripps Institutional Review Board. This study followed the Consolidated Health Economic Evaluation Reporting Standards (CHEERS) reporting guideline for economic evaluations.

Prescription-level pharmacy claims data from January 1, 2012, through December 31, 2017, were obtained from the Blue Cross Blue Shield (BCBS) Axis,^17^ a database that includes administrative claims data from independent BCBS companies representing more than 35 million individuals with private pharmaceutical insurance across the United States. Composition of the BCBS Axis data is proprietary but reflects the geographic distribution of BCBS companies. Data were housed within a Microsoft SQL server managed by the BCBS Association and accessed remotely through a secure data portal. Data were prepared in November 2018, and BCBS Association employees aided our group in accessing relevant data but imposed no control over the research or publication decisions. BCBS companies allow HIPAA (Health Insurance Portability and Accountability Act of 1996)–compliant access to BCBS Axis data for research purposes.

The top-selling branded prescription drugs of 2017 were identified on the basis of total sales exceeding $500 million in the United States or exceeding $1 billion worldwide (when US sales data were not available).^18^ All pharmacy claims for each of these drugs were extracted from the database using Food and Drug Administration (FDA) National Drug Code (NDC) identifiers.^19^ Drugs that are typically administered in a clinical setting or not distributed through a pharmacy are not well represented in pharmacy claims data and were omitted from downstream analyses. The most common prescription of each drug was identified according to a combination of its NDC (in instances of a drug with multiple codes) and its billed quantity dispensed in the prescription (eTable 1 in the Supplement).

The study included drugs that reached more than 100 000 total pharmacy claims and were covered under pharmacy insurance for at least 3 years. In cases in which the same drug was found under different trade names, the most common trade name was used. Proprietary estimates of quarterly net prices of these drugs were obtained from SSR Health.^20^ These metrics were based on a comparison between quarterly estimates of third-party vendor pharmaceutical unit volumes and product-level net sales reported by manufacturers in the same quarter. Nondrug products (eg, vaccinations) were omitted, and drug approval dates and approved therapeutic equivalents were extracted from https://www.fda.gov/.

### Data Presentation

Total price paid of each claim, representing the sum of out-of-pocket cost paid by a plan member and cost paid by the insurer, was the primary outcome of interest. In instances in which the billed unit quantity differed from an individual claim and the most commonly billed unit quantity, the costs were normalized to the most commonly billed quantity by calculating the cost per billed unit. For example, a paid amount of $100 for 1 unit and $200 for 2 units for the same drug would both be considered $100 per unit. Median costs for the most common prescriptions were summarized in each calendar month. In general, measures of variation (eg, interquartile range) were small and are not presented in the results.

Relative price changes were found by calculating the difference in median costs between 2 dates and then scaling this difference by the preceding date’s median cost; the following formula was used: (S_2_-S_1_)/S_1_, in which S_1_ was the median cost for the most common prescription on the first date and S_2_ was for the second date. Relative price changes are presented here as the change in price from January 1, 2012, to December 31, 2017. For drugs that were not on the market in January 2012, the earliest available date was used to find S_1_, and a 6-year relative price change was also extrapolated by scaling the relative price change to a 72-month period (ie, double the time if the drug had been available for only the minimum 36 months). Likewise, a 3-year relative price change was calculated for all drugs from January 1, 2015, to December 31, 2017, for a more appropriate direct comparison of drugs that had entered the market after January 1, 2012, with those on the market before January 1, 2012. Month-to-month relative price changes were identified by calculating the difference in median costs between consecutive months and then scaling the difference by the preceding month’s median cost using the following formula: (S_i_-S_i-1_)/S_i-1_, in which S_i_ is the median cost for the most common prescription during the i-th month.

### Statistical Analysis

The association between relative price changes and therapeutic equivalents was assessed using a 2-sided *t* test. Correlation in month-to-month relative price changes between all pairs of drugs was calculated with Spearman rank correlation. The means of the quarterly estimates of net price-per-unit quantity were computed over a 4-quarter or 1-year interval. The mean annual net price increase was obtained by comparing the mean net prices in 2012 with those in 2017. For new drugs that entered the market, the first 4 quarters of data available were used (because of unpredictable volume patterns at launch, the first 2 quarters of a product’s patented protected commercial life were excluded from the SSR Health data). In either case, these differences were normalized by years of data. For example, a drug with a $100 net price per unit in 2012 and $200 in 2017 would have a mean annual increase of 20% each year for 5 years. Pearson correlation between these mean annual net price increases and the mean median price increase within the BCBS data was assessed with linear regression. All analyses were performed in R, version 3.3.2 (R Project for Statistical Computing). Statistical tests were 2-sided using a significance level of *P* < .05.

## Results

A total of 132 brand-name prescription drugs were identified in 2017 that met the criteria of exceeding $500 million in US sales or $1 billion worldwide. Among these products, 61 drugs had greater than 100 000 pharmacy claims in the BCBS Axis from January 1, 2012, through December 31, 2017, with 55 drugs available and covered under pharmacy insurance for at least 3 years (41 for the entire 6-year observation period) and 49 drugs with net price data available from SSR Health. This study focused on these 49 drugs, and eTable 2 in the Supplement shows data on these 49 drugs and the remaining 83 products.

Claims for 13 (27%) of the 49 drugs were first found after January 1, 2012, with claims for the remaining 36 drugs occurring throughout the observation period. The median time from FDA approval until the end of the observation period (December 31, 2017) for all drugs was 11.6 years. Seventeen drugs (35%) had FDA-approved therapeutic equivalents (or the same active ingredient, as in the case of Humulin and insulin). For reference, the term of a new patent filed in the United States is 20 years from the date of application, and new chemical entity exclusivity rights granted by the FDA last for 5 years regardless of whether the drug is protected under a patent.

Median total costs for the most common prescriptions of each of the 49 high-volume brand-name drugs from 2012 through 2017 are presented in the Table. Summarized costs by month are available in eTable 3 in the Supplement. Median cost increase of these drugs was 76% during a 6-year period from January 2012 through December 2017 (extrapolated for drugs that were not available in the entire period) or 9.8% compounded annually. Almost all drugs (48 [98%]) displayed regular annual or biannual price increases. Only Harvoni ($30 920 median cost per prescription as of December 31, 2017) decreased in cost over time, although no more than 1% annually. Of the 36 drugs that have been available since 2012, 28 (78%) have seen an increase in insurer and out-of-pocket costs by more than 50%, and 16 (44%) have more than doubled in price. In total, 17 drugs (35%) more than doubled in costs, including Chantix, Cialis, Forteo, Lexapro, Lipitor, Lyrica, Onfi, Premarin, Renvela, Simponi, Viagra, and Zetia; tumor necrosis factor inhibitors Enbrel and Humira; and insulins Humalog, Humulin, and Novolog. The median time since FDA approval among these 17 drugs was 15.2 years. However, no discernible difference in relative price increase was found between the 13 drugs that entered the market after January 1, 2012, and the remaining 36 drugs between January 1, 2015, through December 31, 2017, that were on the market during the past 3 years (median, 29% over those 3 years; *P* = .81). Similarly, the 6-year relative price increase of the 17 drugs with therapeutic equivalents was not different from the 6-year relative price increase of the other 32 drugs without therapeutic equivalents (median, 79% vs 73%; *P* = .21).

**Table.** Median Total Cost of Top-Selling Brand-Name Drugs, 2012- 2017

Brand Name	Treatment or Condition	Median Cost, US$		6-y Change, %
		January 2012	December 2017	
Advair	COPD	225	360	60
Androgel	Testosterone	321	566	76
Atripla	HIV	1776	2531	43
Brilinta	Anticoagulant	236	333	41
Chantix	Smoking cessation	175	392	124
Cialis	Erectile dysfunction	127	365	187
Creon	Exocrine pancreatic insufficiency	293	487	66
Crestor	Cholesterol	146	261	79
Eliquis	Anticoagulant	258	388	50^a^
Enbrel	Autoimmune disease	1862	4334	133
Farxiga	Type 2 diabetes	318	431	35^a^
Forteo	Osteoporosis	1116	3088	177
Harvoni	Hepatitis C	31 752	30 920	−3^a^
Humalog	Insulin	126	274	117
Humira	Autoimmune disease	1940	4338	124
Humulin	Insulin	67	146	117
Invokana	Type 2 diabetes	295	427	58^a^
Isentress	HIV	1005	1379	37
Januvia	Type 2 diabetes	219	396	80
Lantus	Insulin	212	384	82
Lexapro	Depression	120	300	150
Lipitor	Cholesterol	116	274	137
Lyrica	Pain	174	411	137
Nexium	Gastroesophageal reflux	188	252	34
Novolog	Insulin	244	532	118
Onfi	Seizures	496	996	118^a^
Orencia	Autoimmune disease	2482	3777	55^a^
Otezla	Psoriasis	1913	3118	61^a^
Premarin	Menopause	68	156	129
Prezista	HIV	1119	1454	38^a^
Pulmicort	Asthma/IBD	151	216	43
Renvela	Kidney disease	212	501	136
Restasis	Immunosuppression	266	463	74
Simponi	Autoimmune disease	1978	4094	107
Stelara	Psoriasis	5420	9213	70
Stribild	HIV	2402	3069	28^a^
Symbicort	Asthma/COPD	225	308	37
Synthroid	Thyroid	20	35	72
Tivicay	HIV	1200	1526	28^a^
Triumeq	HIV	2239	2578	16^a^
Trulicity	Type 2 diabetes	497	674	35^a^
Truvada	HIV	1188	1557	31
Viagra	Erectile dysfunction	127	370	190
Victoza	Type 2 diabetes	433	805	86
Viread	HIV	746	1057	42
Vyvanse	ADHD	162	270	67
Xarelto	Anticoagulant	225	386	72
Xeljanz	Autoimmune disease	2108	3757	79^a^
Zetia	Cholesterol	126	313	149

Abbreviations: ADHD, attention-deficit/hyperactivity disorder; COPD, chronic obstructive pulmonary disease; IBD, inflammatory bowel disease.

^a^ Drug was not available during the entire study period; the amount indicates the relative price change from first month of claim occurrences (January 2012 entry included data from the first month of occurrence).

**Figure 1.**
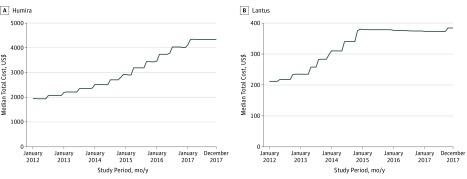
Median Total Costs Paid for Humira and Lantus

For most of the drugs examined, a steadily increasing cost trend was observed. The month-by-month median cost of Humira (Figure 1A) was representative of the general trend observed for other drugs examined (eFigure 1 in the Supplement). For most of these drugs, costs generally increased 1 to 2 times per year, often near the beginning or middle of the calendar year. Much rarer was any leveling off of costs, such as that observed from Lantus beginning in 2015 (Figure 1B), although this leveling off occurred after a 79% increase in total costs over a 3-year span.

Owing to the general cost-increasing trajectories shared across drugs, a number of nonzero correlations in the monthly relative cost changes were observed between drug pairs (eFigure 2 in the Supplement). Inspection of pairs of brand-name drug competitors that treat similar conditions demonstrated, generally, even higher correlated cost changes. For example, the cost trend of Humira was most highly correlated with that of its competitor Enbrel (highest of 48 pairwise correlations ρ = 0.53; *P* = 2.5 × 10^−6^). The costs of Humalog and Novolog were similarly most correlated with each other (ρ = 0.63; *P* = 3.5 × 10^−9^), with Lantus most correlated with Humalog (ρ = 0.38; *P* = 9.3 × 10^−4^; first of 48) and also with Novolog (ρ = 0.28; *P* = .02; second of 48).

**Figure 2.**
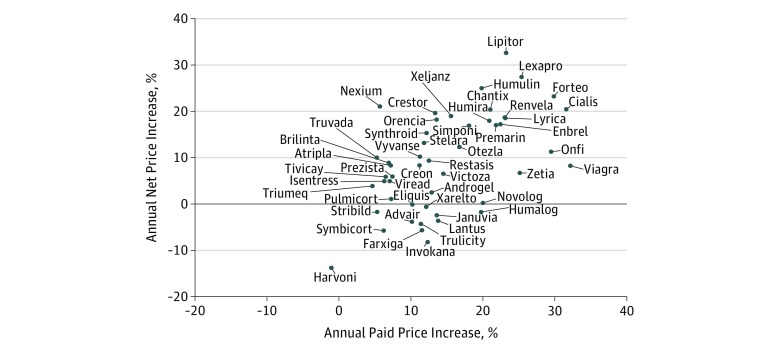
Comparison of Annual Net Price Percentage Increase and Annual Paid Price Percentage Increase for All Drugs Examined

Quarterly estimates of net price per unit were obtained for each drug. Thirty-five drugs (71%) had 24 quarters (6 years) of data from 2012 through 2017. The mean of the annual net price increases across all 49 drugs was 9.0% (95% CI, 6.1%-11.9%; *P* = 1.9 × 10^−7^). These net price rates were correlated with the paid insurer and out-of-pocket cost rates obtained from BCBS data across all drugs (ρ = 0.55; *P* = 3.8 × 10^−5^) (Figure 2). Mean annual net price rates were lower among the 26 drugs with FDA approval after January 1, 2005, compared with drugs approved before January 1, 2005 (mean, 5.8% vs 12.3%; *P* = .02). Net price rates remained correlated with drug cost rates among the 26 drugs (ρ = 0.60; *P* = 1.2 × 10^−3^).

## Discussion

This study presents drug cost data from more than 35 million US individuals with private pharmaceutical insurance. These data demonstrate an industry-wide trend of substantial increases in costs for top-selling brand-name prescription drugs from 2012 through 2017. Because most products displayed continual, marked annual increases throughout the observation window, we expect these products to continue along this price escalation course, along with emerging products. Given the median annual cost increase of 9.5%, our results suggest the costs for popular brand-name drugs would double every 7 to 8 years. Competition among brand-name competitors appeared to do little to stymie rising costs. Instead, products that may be prescribed interchangeably, such as Humira and Enbrel or Humalog, Humulin, and Novolog, were highly synchronized in relative cost changes while demonstrating some of the largest cost increases in the industry over the past 6 years. Such seeming coordination coinciding with high price increases is particularly worrisome.

We did not see any evidence of price changes being associated with the existence of therapeutic equivalents. This finding suggests that prices of brand-name drugs are not largely affected by the presence of generic drugs or perhaps biosimilar products and others that may enter the market in the future. Implementation of the price transparency legislation passed in October 2018 may guide patients to seek lower-priced alternatives to brand-name drugs when available, which may ultimately lead to different price trends in the future compared with the trends we observed. However, this likelihood is unknown. Even if patients and clinicians preferred generic alternatives (as they may given the increase in the proportion of generic prescriptions filled^4^), it is not certain if the trends we observed are not already a function of volume changes or speculation of upcoming volume changes as each drug approaches and surpasses the end of its federally protected exclusivity periods.

In addition, we did not find evidence that products that entered the market 3 to 6 years ago have different trends compared with other drugs in the first years of availability. This finding, along with the consistent, once- or twice-a-year price increases of most drugs we examined, implies that this cycle will persist throughout the lifetime of a drug in the current, private pharmaceutical insurance market.

Reasonable drug costs for consumers must be balanced with incentives in the pharmaceutical industry to produce innovative drugs that improve and save lives. The United States provides drug companies with the strongest patent protections in the world, but legal strategies in the pharmaceutical industry, such as patenting peripheral aspects of a drug that extend exclusivity rights beyond the original patent and delay generic and biosimilar competition, abuse that liberty.^21^ The large discrepancy between the prices of drugs purchased in the United States and drugs purchased in the rest of the world^2^ is often attributed to the legal inability of public and private insurers to negotiate drug prices. Innovative solutions, such as the Institute for Clinical and Economic Review’s value-based price benchmark,^22^ have the potential to find appropriate price points for patients while rewarding drug manufacturers that produce transformative products.

### Limitations 

A limitation of this study was the lack of information on rebates and how they affected net prices. Rebates are issued in bulk and cannot be linked to individual claims. Rebates vary by drug and by payer, with 16% of all private insurer-branded drug spending returned as rebates in 2016.^23^ Proponents argue that rebates can reduce costs, whereas opponents argue that pharmaceutical companies simply raise list prices to offset losses from rebates and increase profits. To address the lack of rebate data, we obtained third-party estimates of net price data on each drug. We observed high correlation between increases in the rates of insurer and out-of-pocket costs paid for each drug and the net prices (ρ = 0.55). This association suggests that the offered supposition that higher list prices and greater reliance on rebates reduce costs may be untrue. Instead, increases in list prices, and thus increases in insurer and out-of-pocket costs paid, may coincide with increases in net prices, which in turn make these drugs more expensive overall. Seemingly biannual price increases should not be considered benign pricing strategies to offset paid against net price discrepancies in the current rebate system. Greater transparency is needed.

## Conclusions

The costs of brand-name drugs have risen substantially in the past 6 years, with regular increases occurring 1 to 2 times per year. With so few exceptions to this norm, costs will likely continue to rise unless bold actions are taken.

## Notes

Supplement. **eFigure 1.** Plots of Median Monthly Total Costs for the Most Common Prescription of 49 Branded Drugs From 01/2012 to 12/2017 **eFigure 2.** Spearman Correlation Between Monthly Relative Cost Changes of Pairs of Drugs **eTable 1.** Details on Number of Claims, NDCs, and Billed Quantities for All Brand Name Products. Includes Information on 49 Drugs Identified in Main Report As Well As 83 Products Not Included in Main Report **eTable 2.** Median Monthly Costs for All 132 Products Identified Using NDC and Billed Quantity in eTable 1 **eTable 3.** Median Monthly Costs for 49 Products Included in Main Report. Includes Same Information on These Products as in eTable 2
