# Supplementary material for: Trends in Prices of Popular Brand-Name Prescription Drugs in the United States
Source: JAMA Netw Open. 2019 May 31;2(5):e194791. doi: 10.1001/jamanetworkopen.2019.4791 (PMC6547085; doi:10.1001/jamanetworkopen.2019.4791)
Supplement: Supplement. — eFigure 1. Plots of Median Monthly Total Costs for the Most Common Prescription of 49 Branded Drugs From 01/2012 to 12/2017 eFigure 2. Spearman Correlation Between Monthly Relative Cost Changes of Pairs of Drugs eTable 1. Details on Number of Claims, NDCs, and Billed Quantities for All Brand Name Products. Includes Information on 49 Drugs Identified in Main Report As Well As 83 Products Not Included in Main Report eTable 2. Median Monthly Costs for All 132 Products Identified Using NDC and Billed Quantity in eTable 1 eTable 3. Median Monthly Costs for 49 Products Included in Main Report. Includes Same Information on These Products as in eTable 2 [file jamanetwopen-2-e194791-s001.pdf]

Supplementary Online Content

Wineinger NE, Zhang Y, Topol EJ. Trends in prices of popular brand-name prescription drugs in the United States. *JAMA Netw Open.* 2019;2(5):e194791. doi:10.1001/jamanetworkopen.2019.4791

eFigure 1. Plots of Median Monthly Total Costs for the Most Common Prescription of 49 Branded Drugs From 01/2012 to 12/2017

eFigure 2. Spearman Correlation Between Monthly Relative Cost Changes of Pairs of Drugs

eTable 1. Details on Number of Claims, NDCs, and Billed Quantities for All Brand Name Products. Includes Information on 49 Drugs Identified in Main Report As Well As 83 Products Not Included in Main Report

eTable 2. Median Monthly Costs for All 132 Products Identified Using NDC and Billed Quantity in eTable 1

eTable 3. Median Monthly Costs for 49 Products Included in Main Report. Includes Same Information on These Products as in eTable 2

This supplementary material has been provided by the authors to give readers additional information about their work.

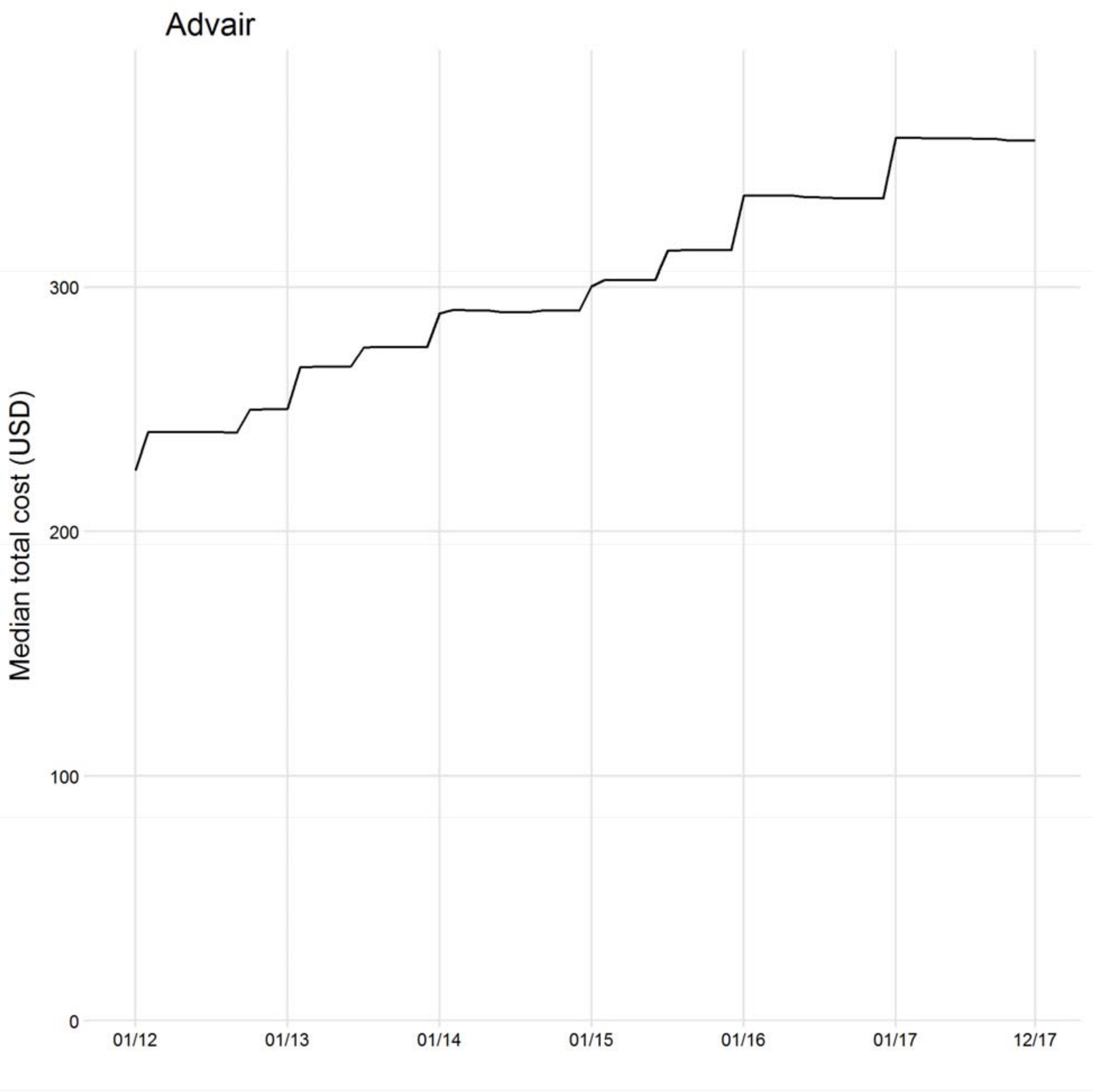

©2019 Wineinger NE et al. *JAMA Network Open*

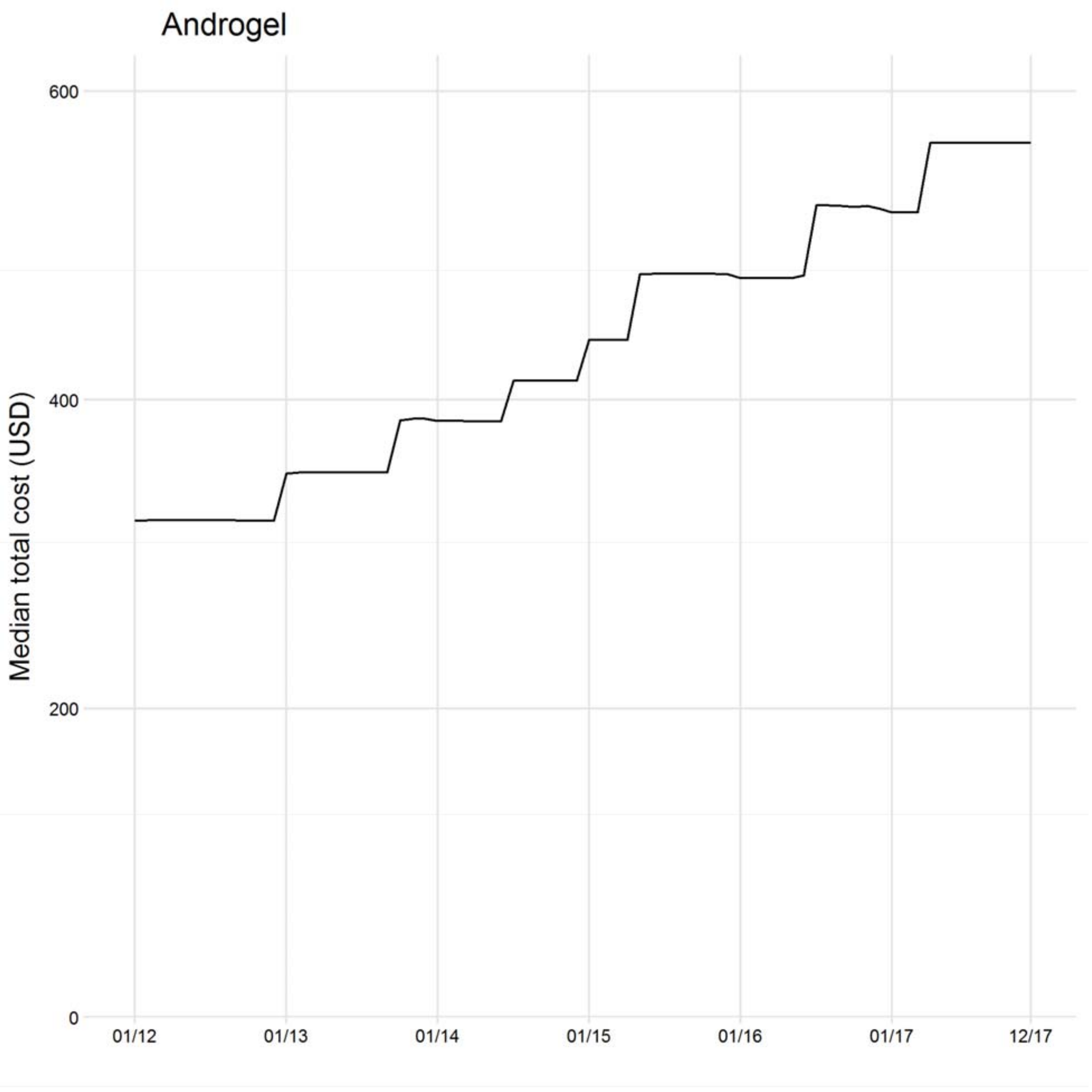

©2019 Wineinger NE et al. *JAMA Network Open*

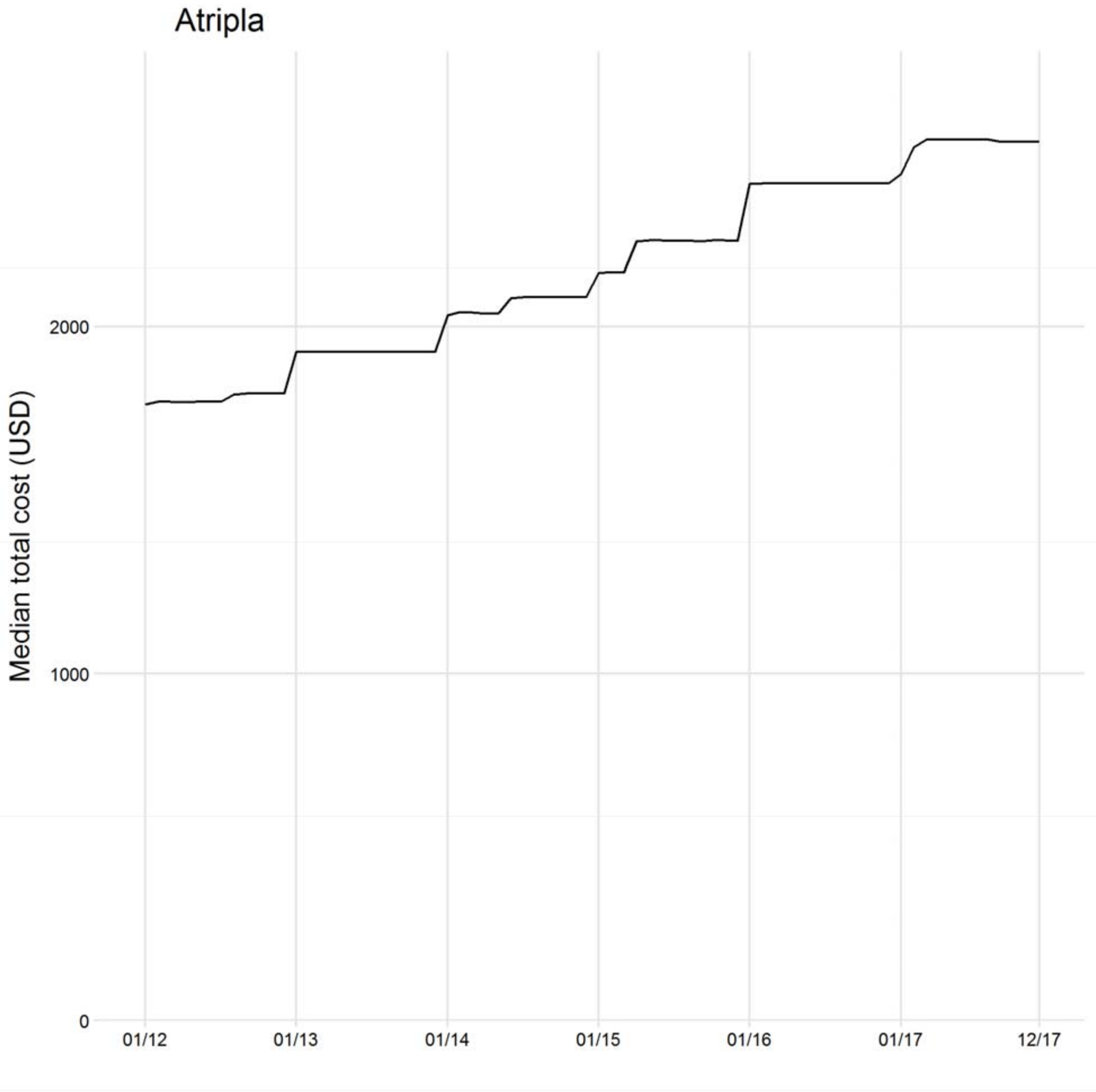

©2019 Wineinger NE et al. *JAMA Network Open*

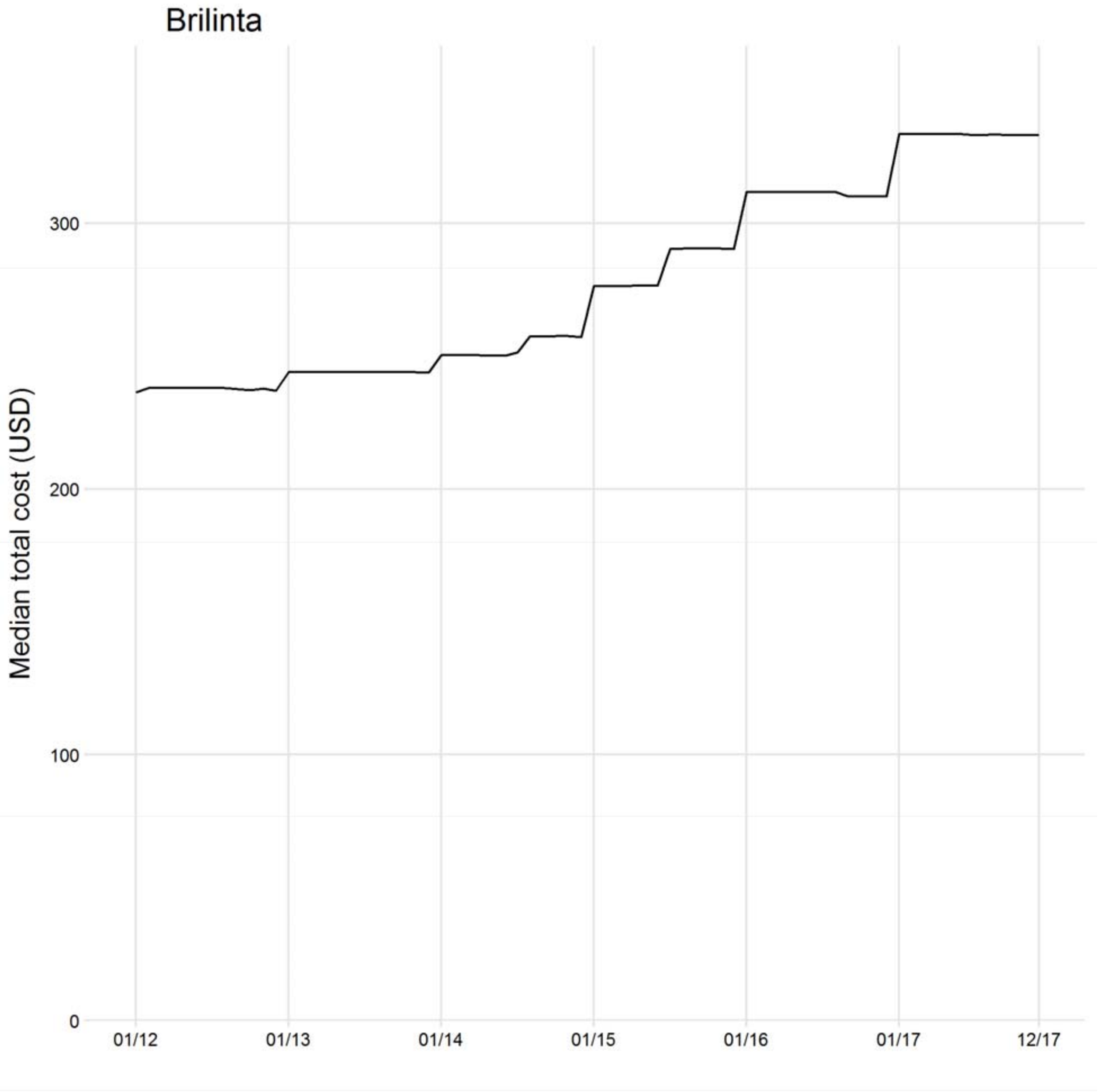

©2019 Wineinger NE et al. *JAMA Network Open*

Chantix

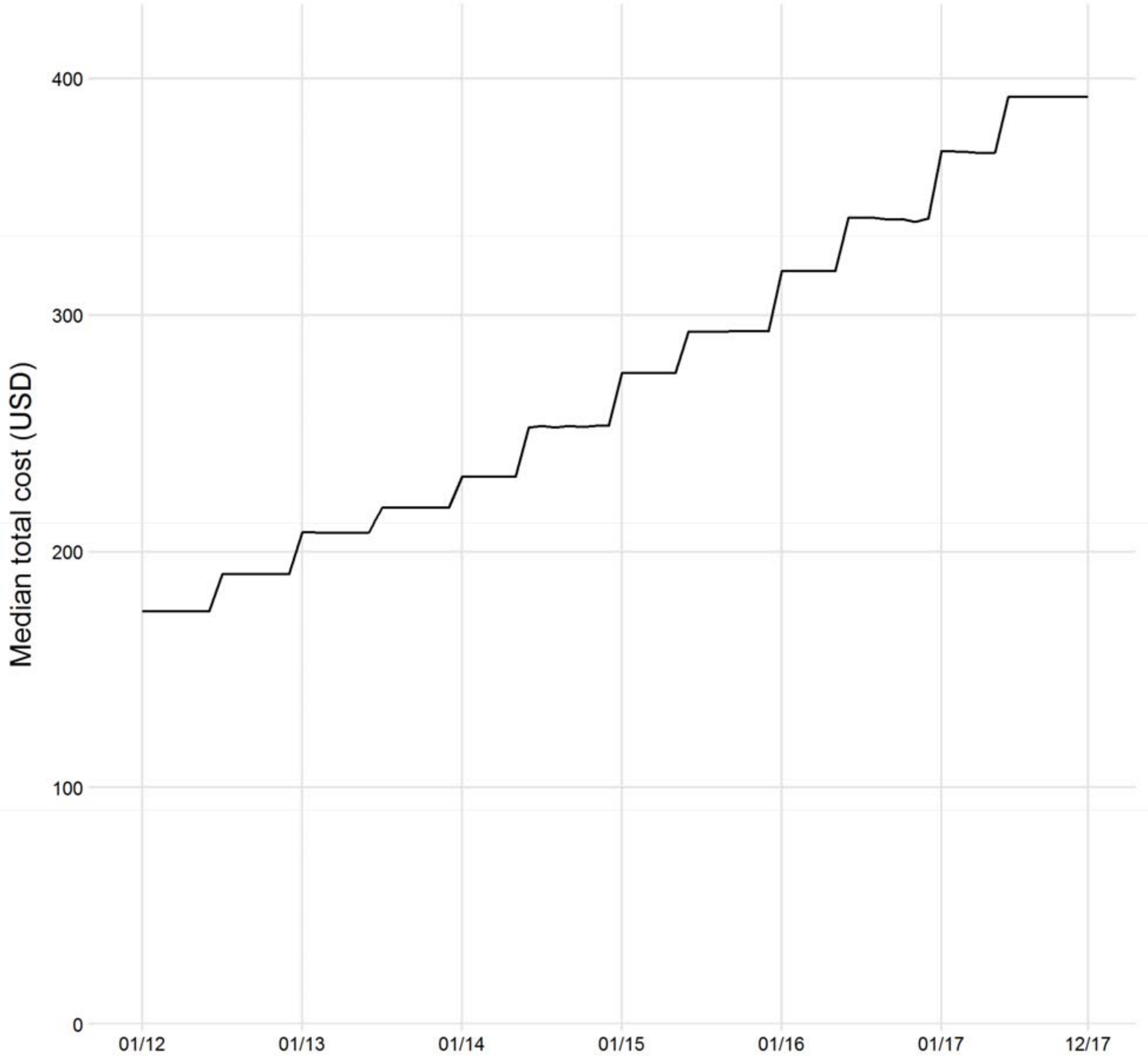

©2019 Wineinger NE et al. *JAMA Network Open*

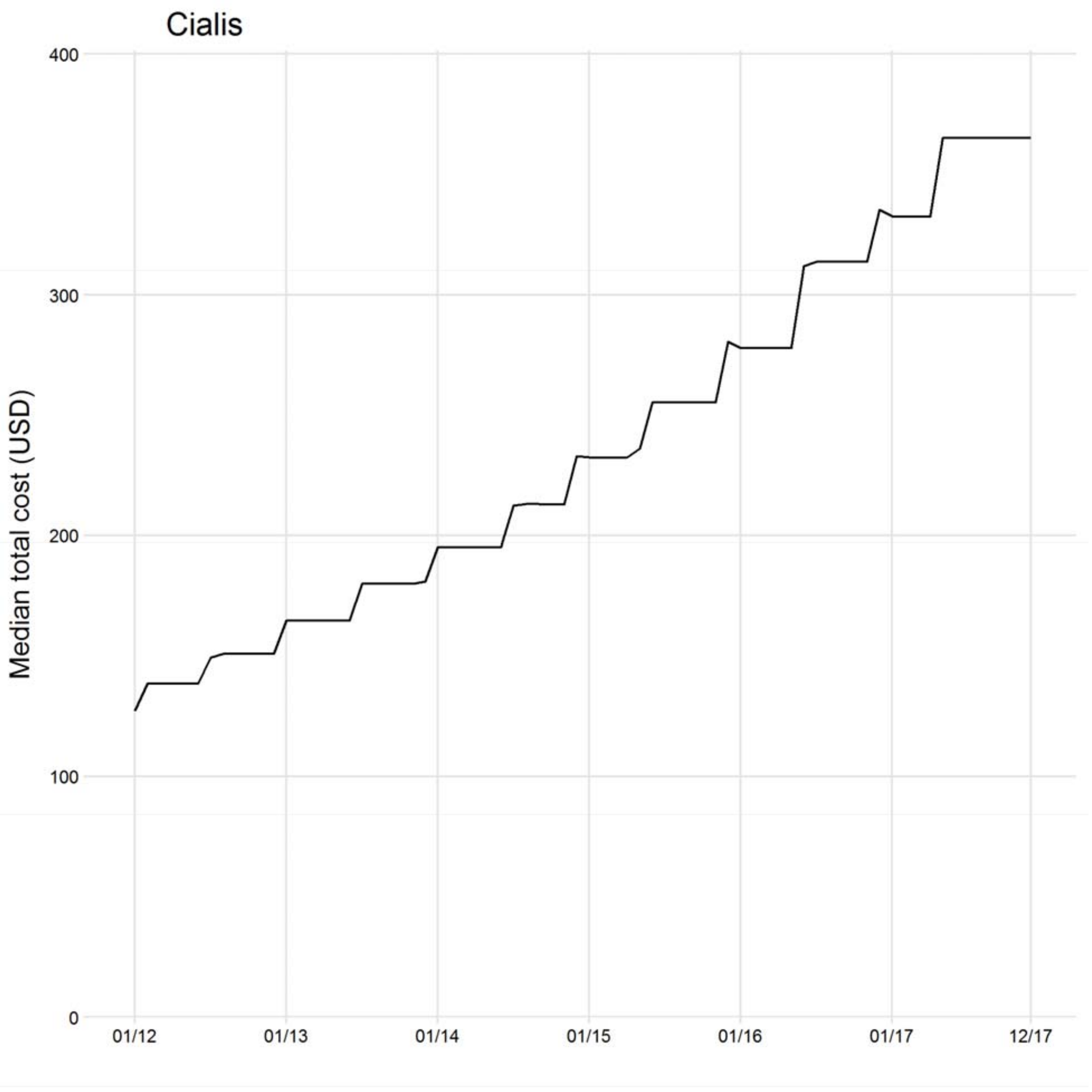

©2019 Wineinger NE et al. *JAMA Network Open*

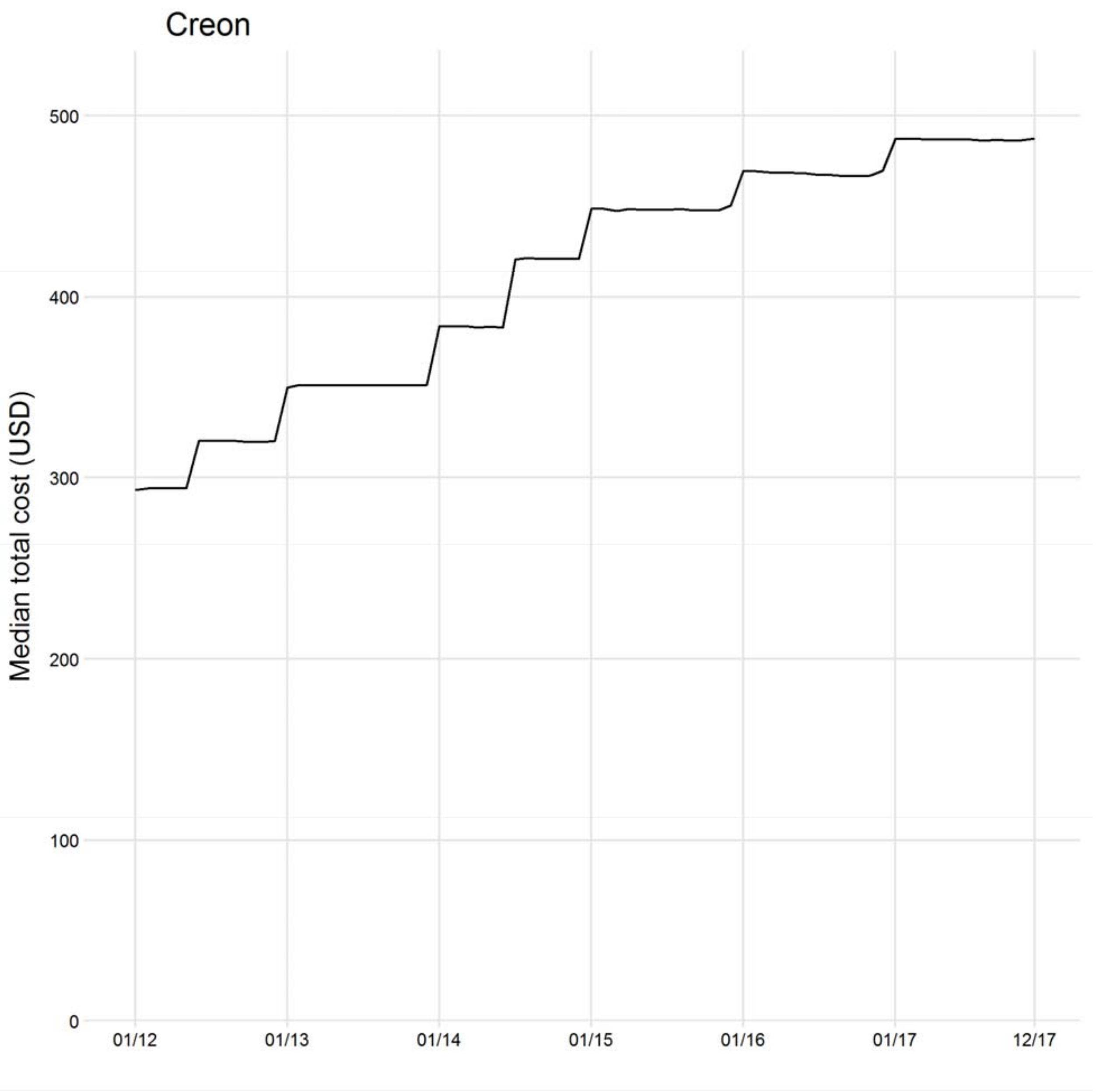

©2019 Wineinger NE et al. *JAMA Network Open*

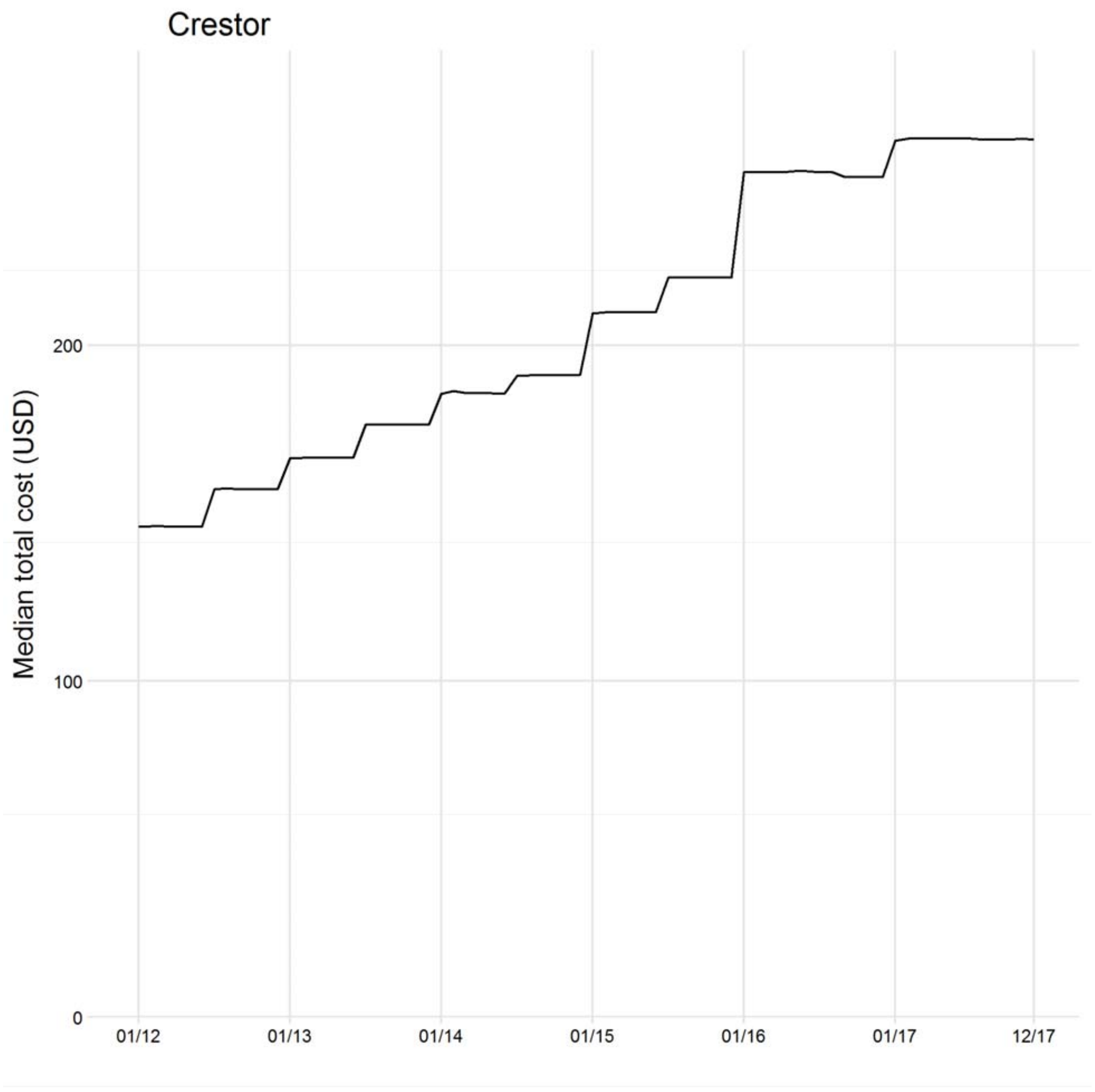

©2019 Wineinger NE et al. *JAMA Network Open*

Eliquis

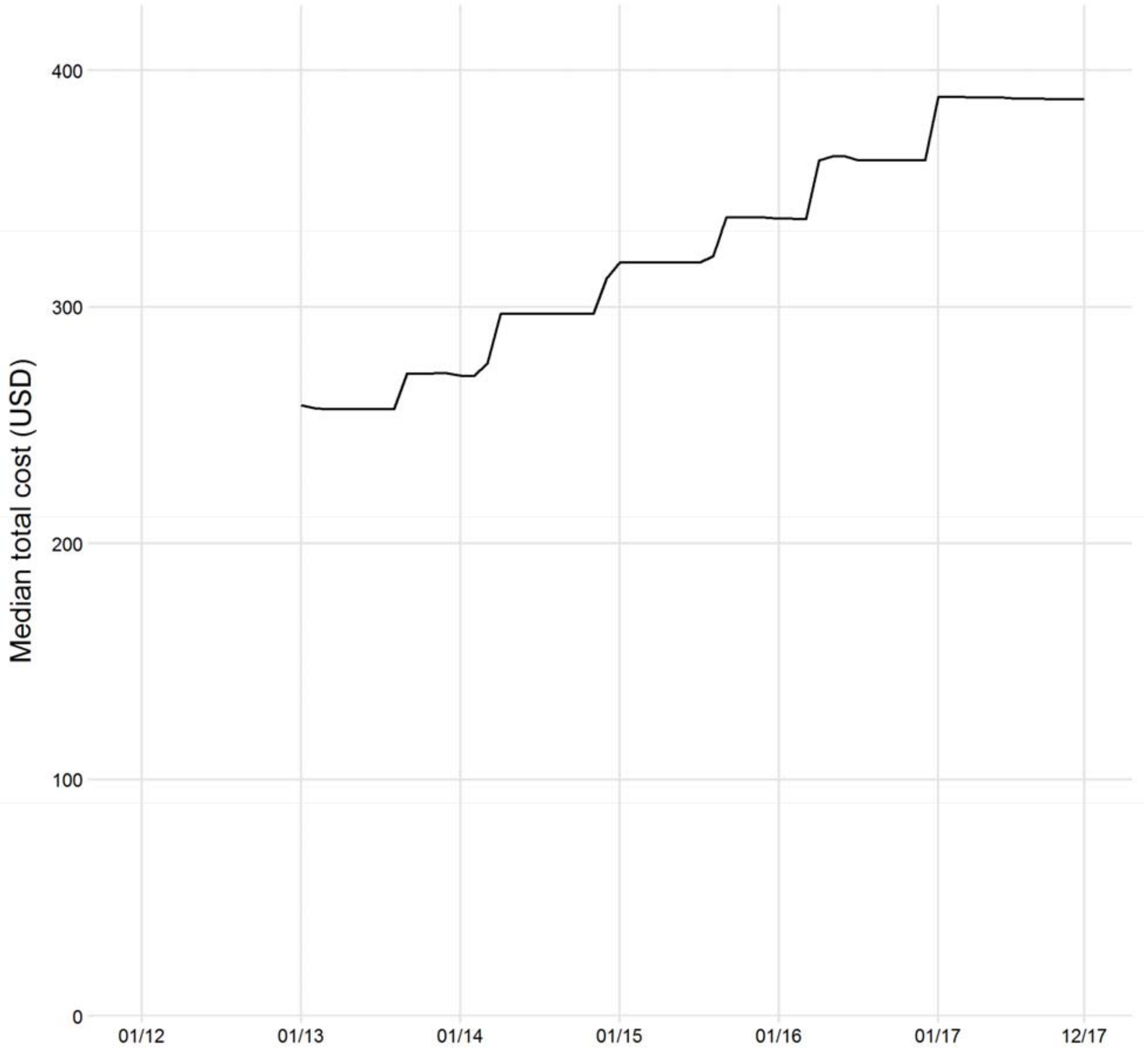

©2019 Wineinger NE et al. *JAMA Network Open*

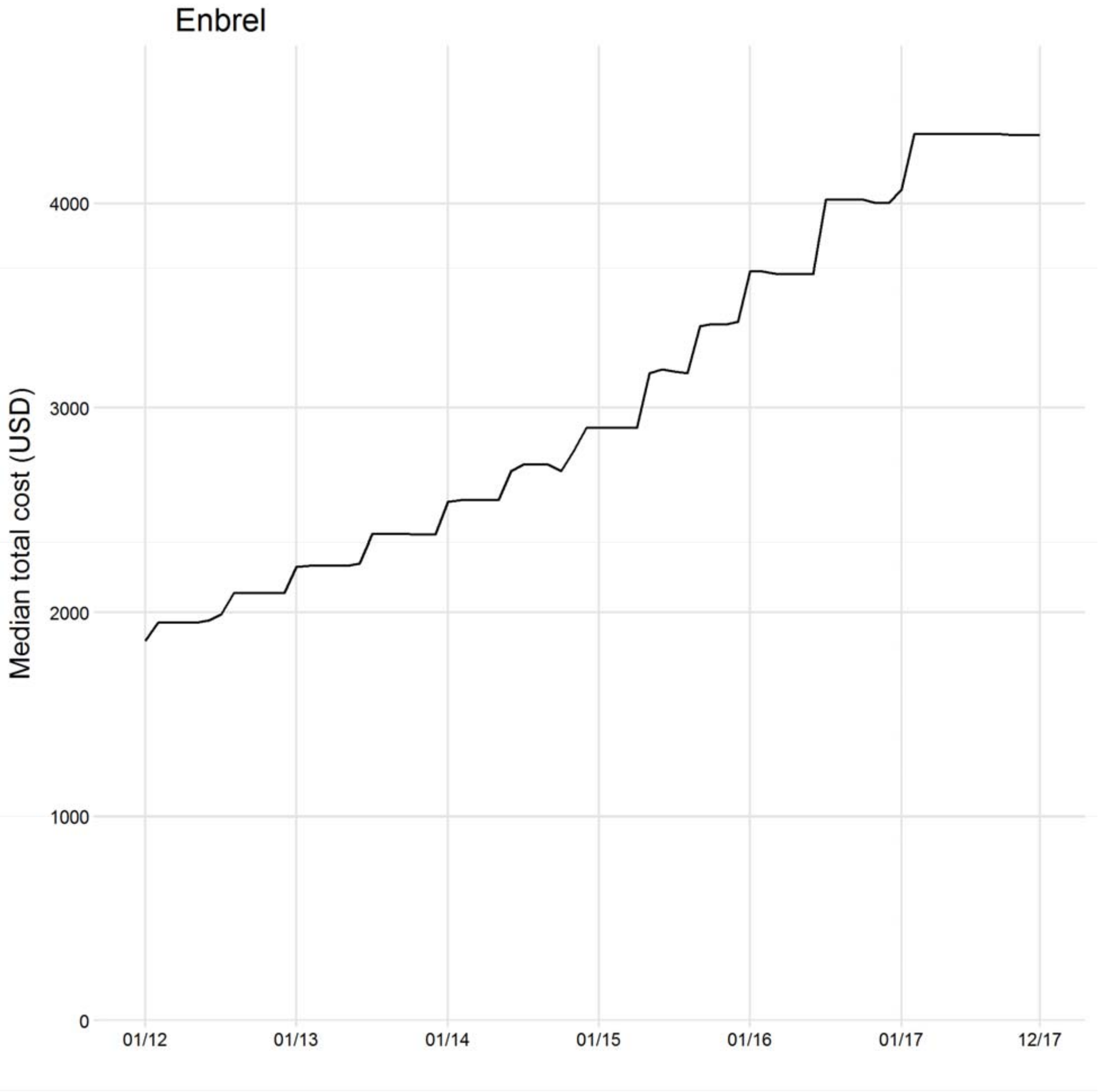

©2019 Wineinger NE et al. *JAMA Network Open*

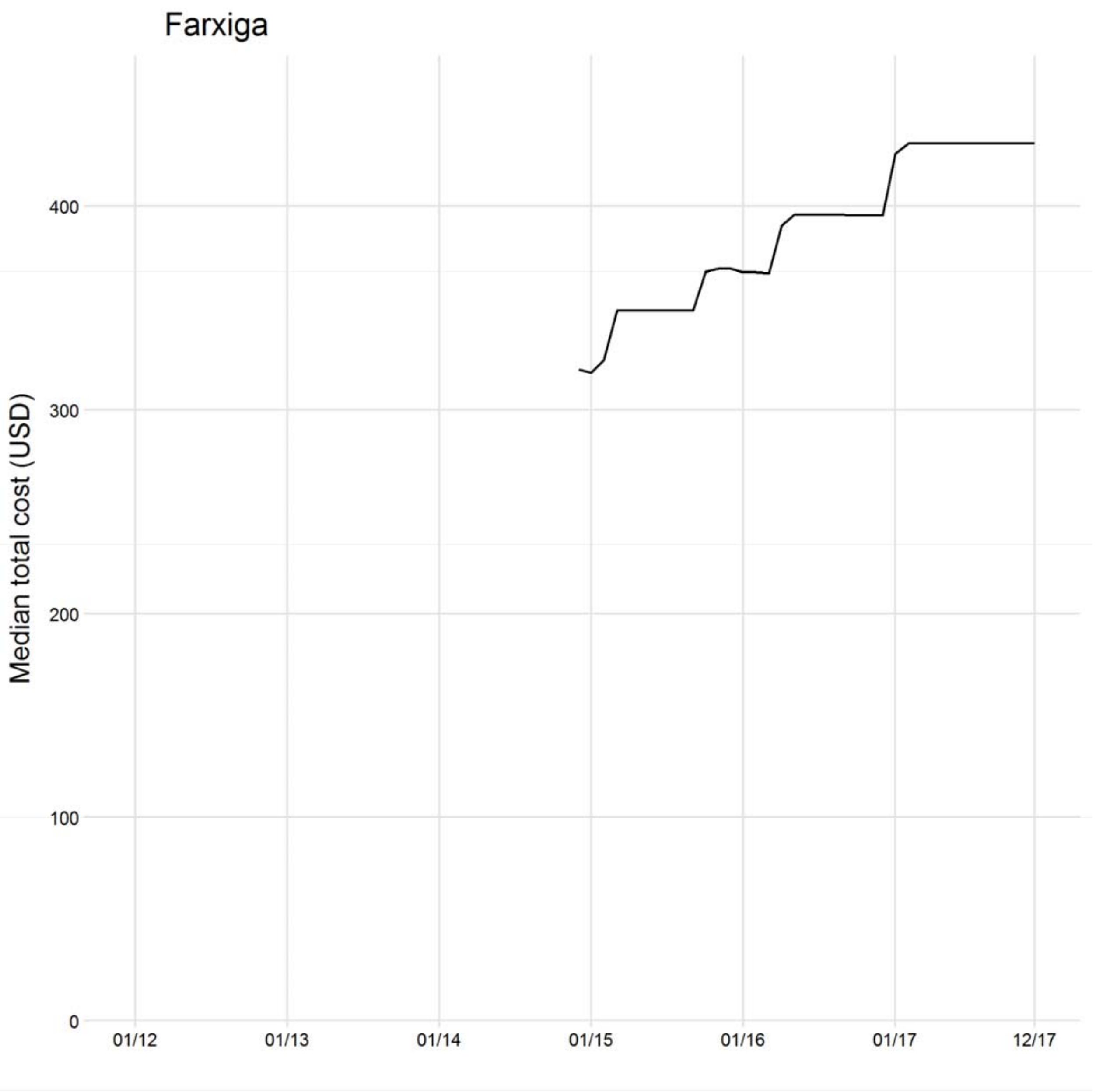

©2019 Wineinger NE et al. *JAMA Network Open*

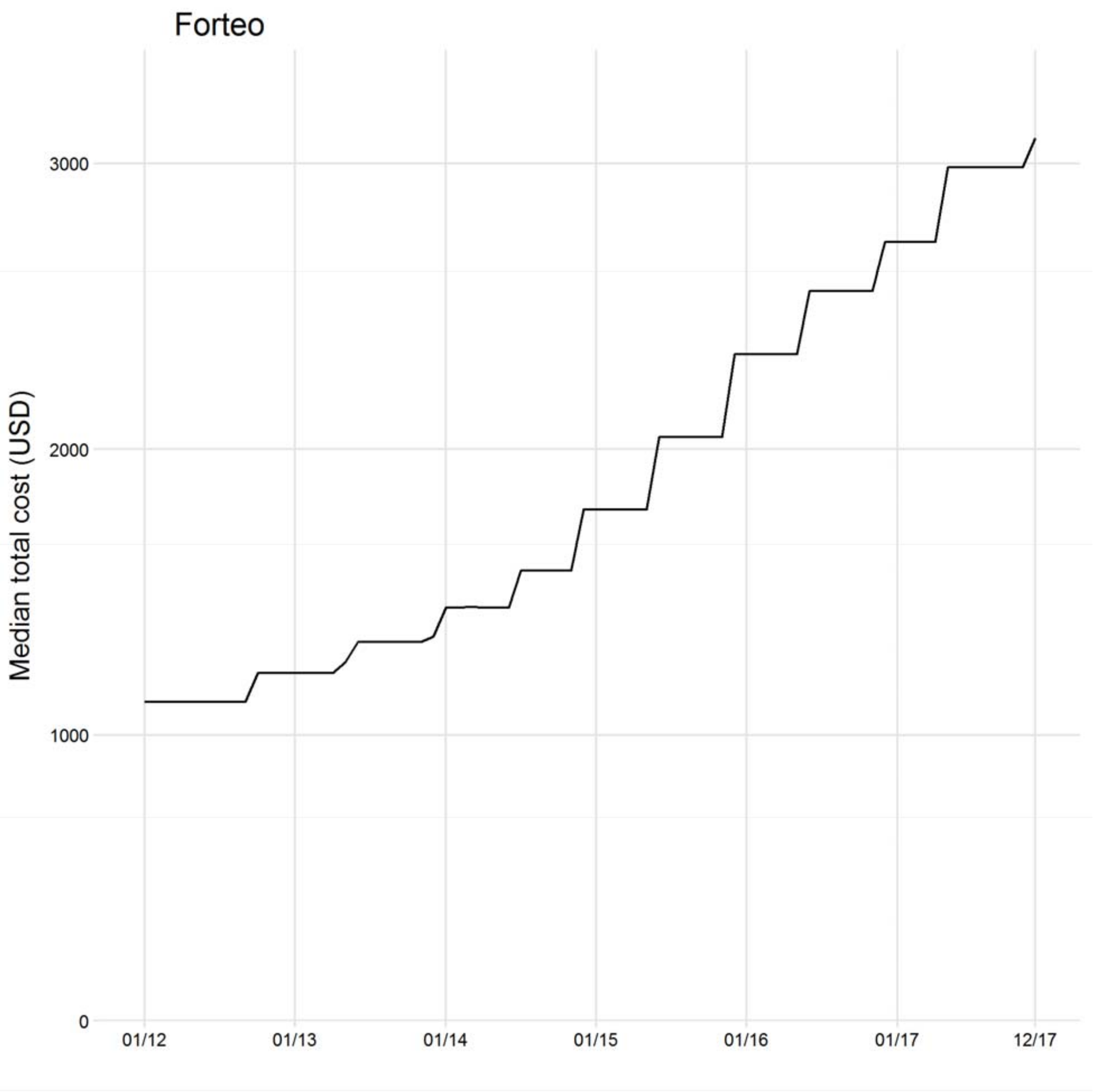

©2019 Wineinger NE et al. *JAMA Network Open*

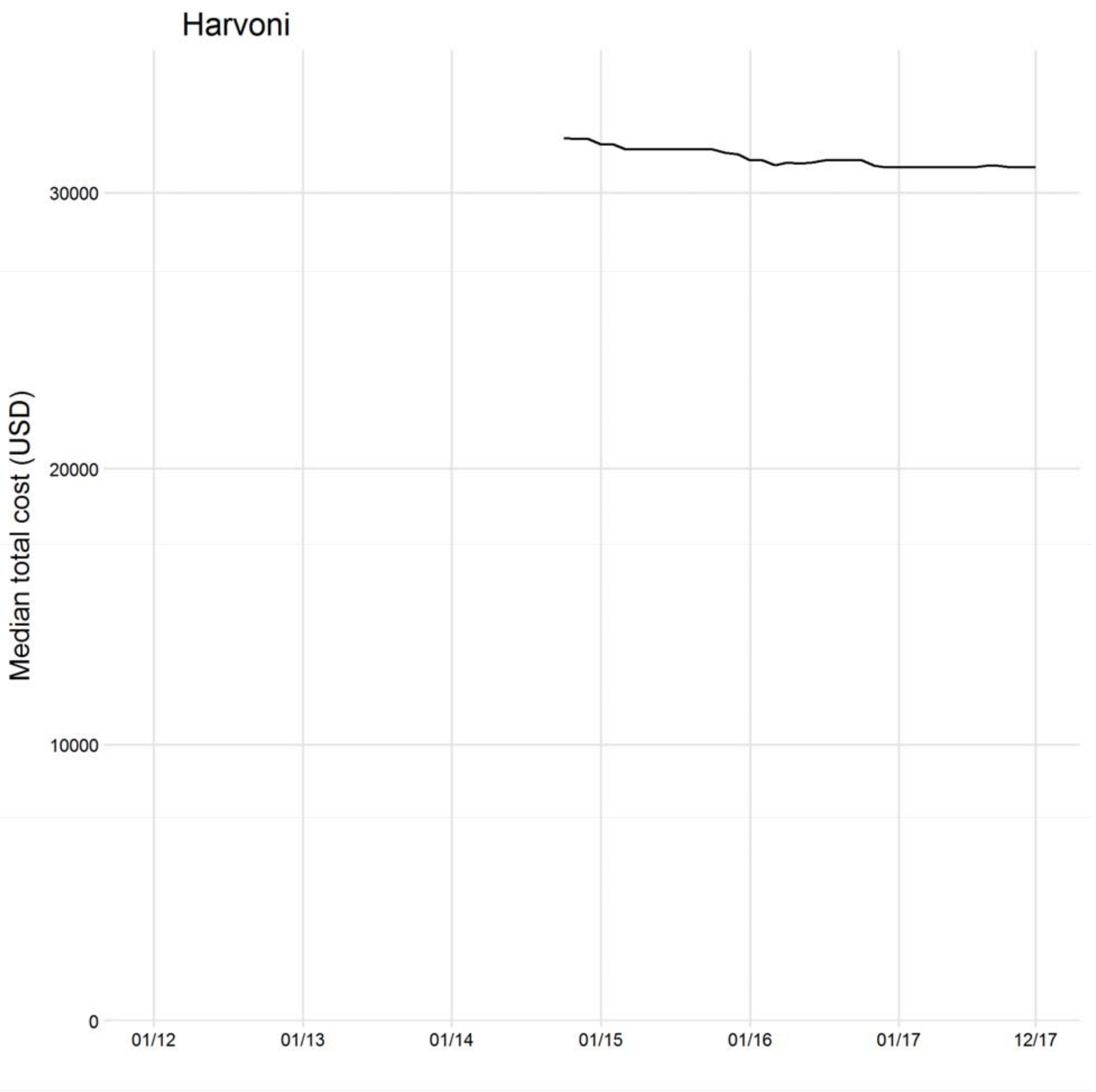

©2019 Wineinger NE et al. *JAMA Network Open*

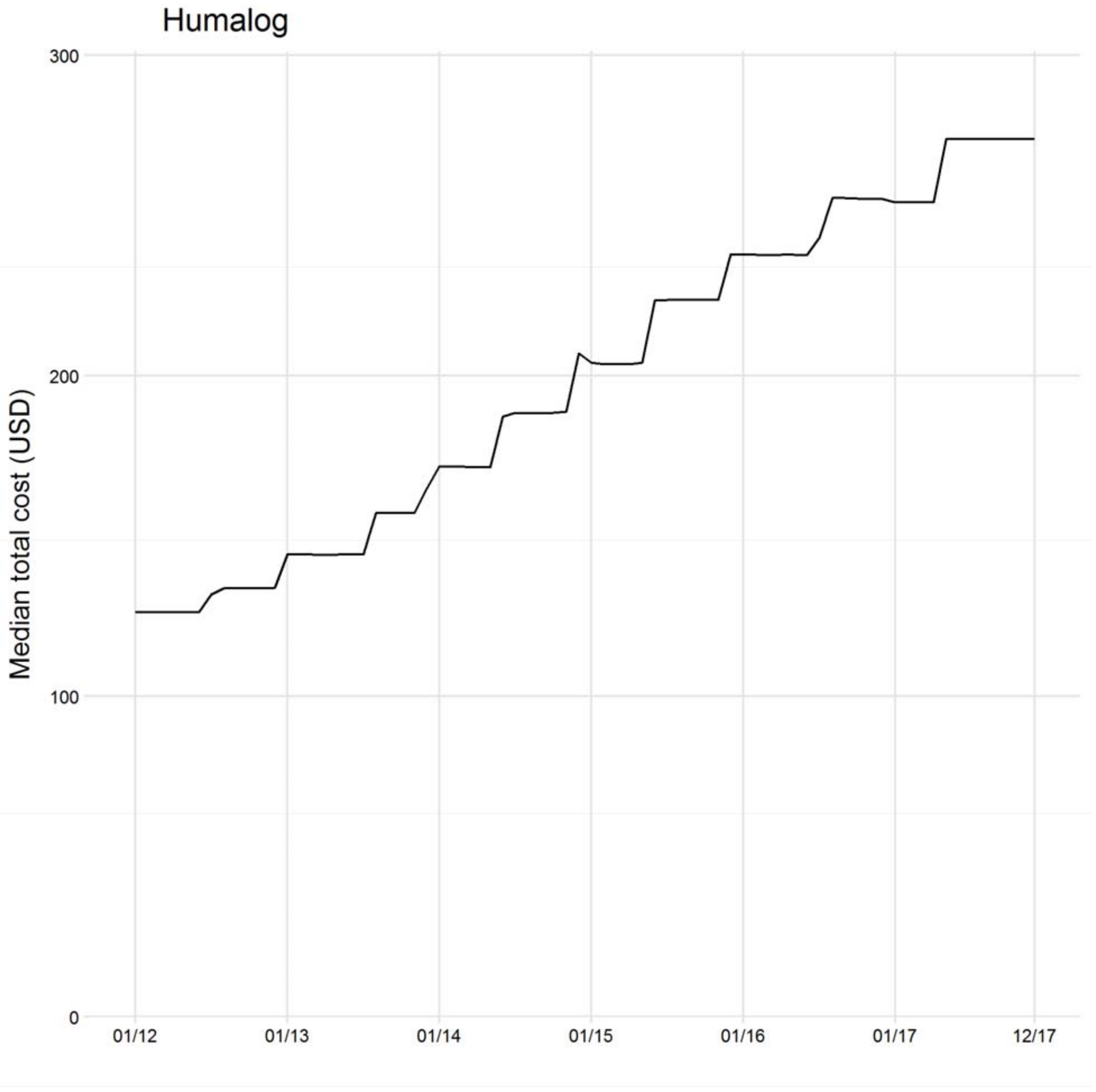

©2019 Wineinger NE et al. *JAMA Network Open*

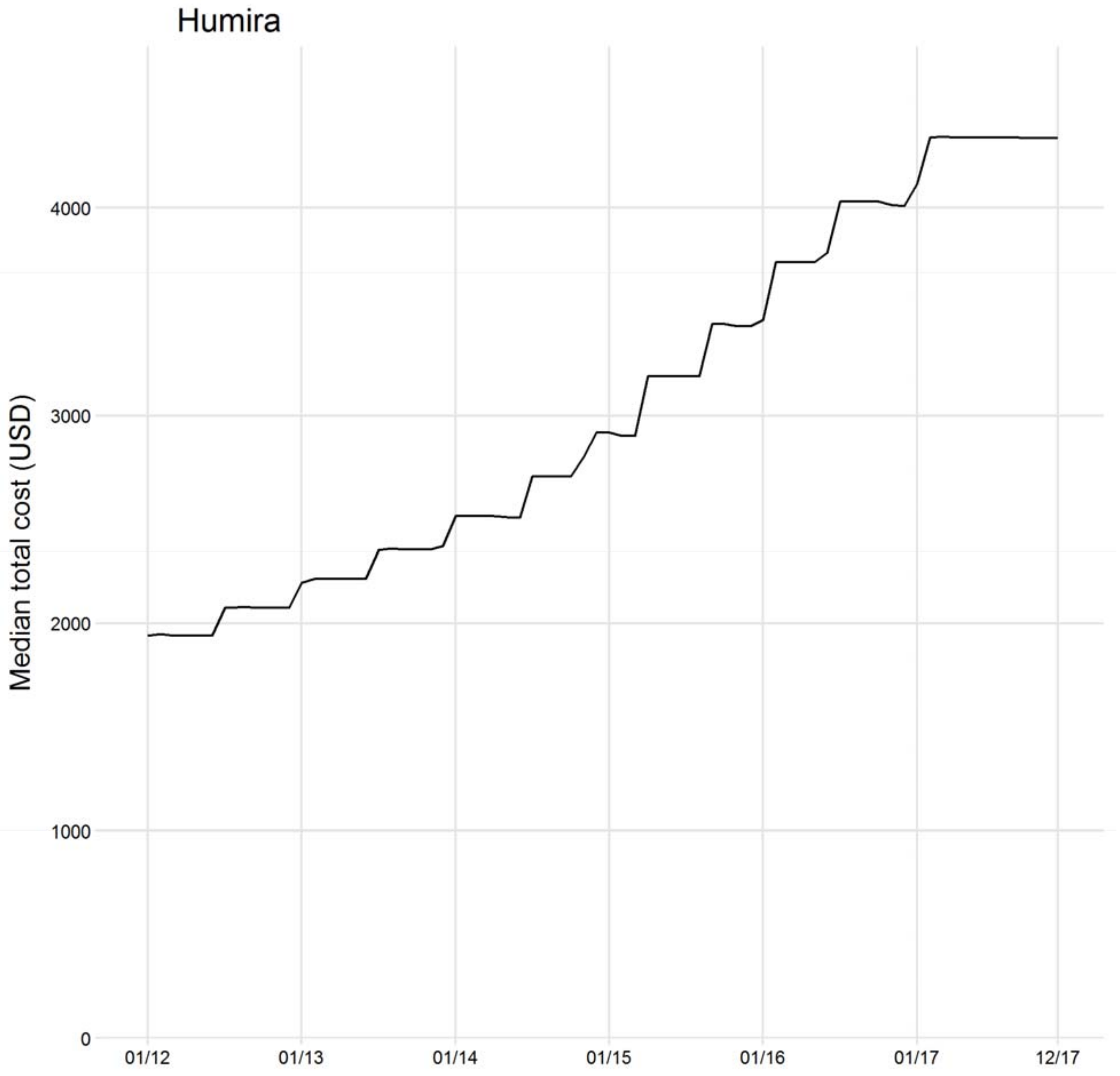

©2019 Wineinger NE et al. *JAMA Network Open*

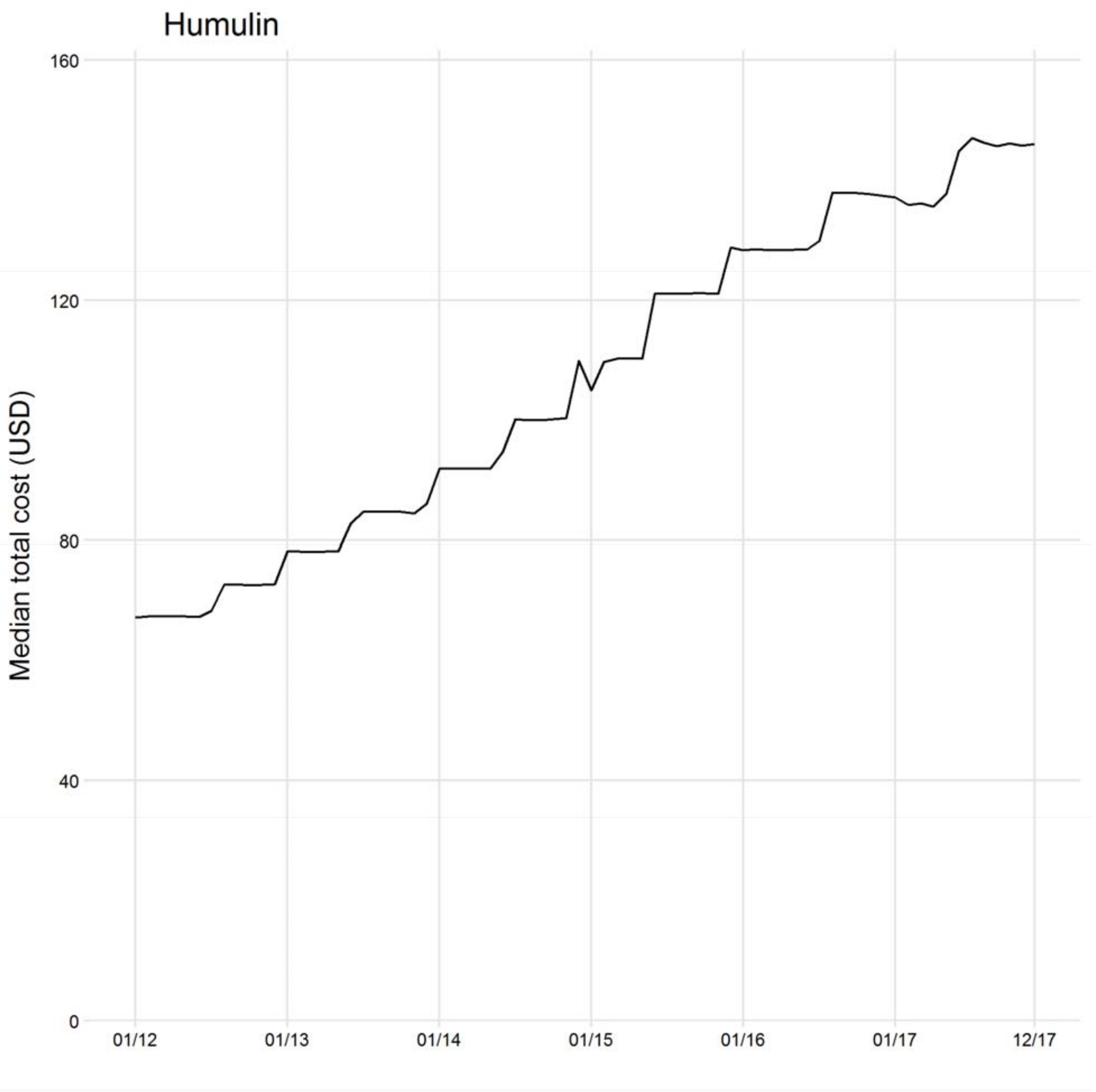

©2019 Wineinger NE et al. *JAMA Network Open*

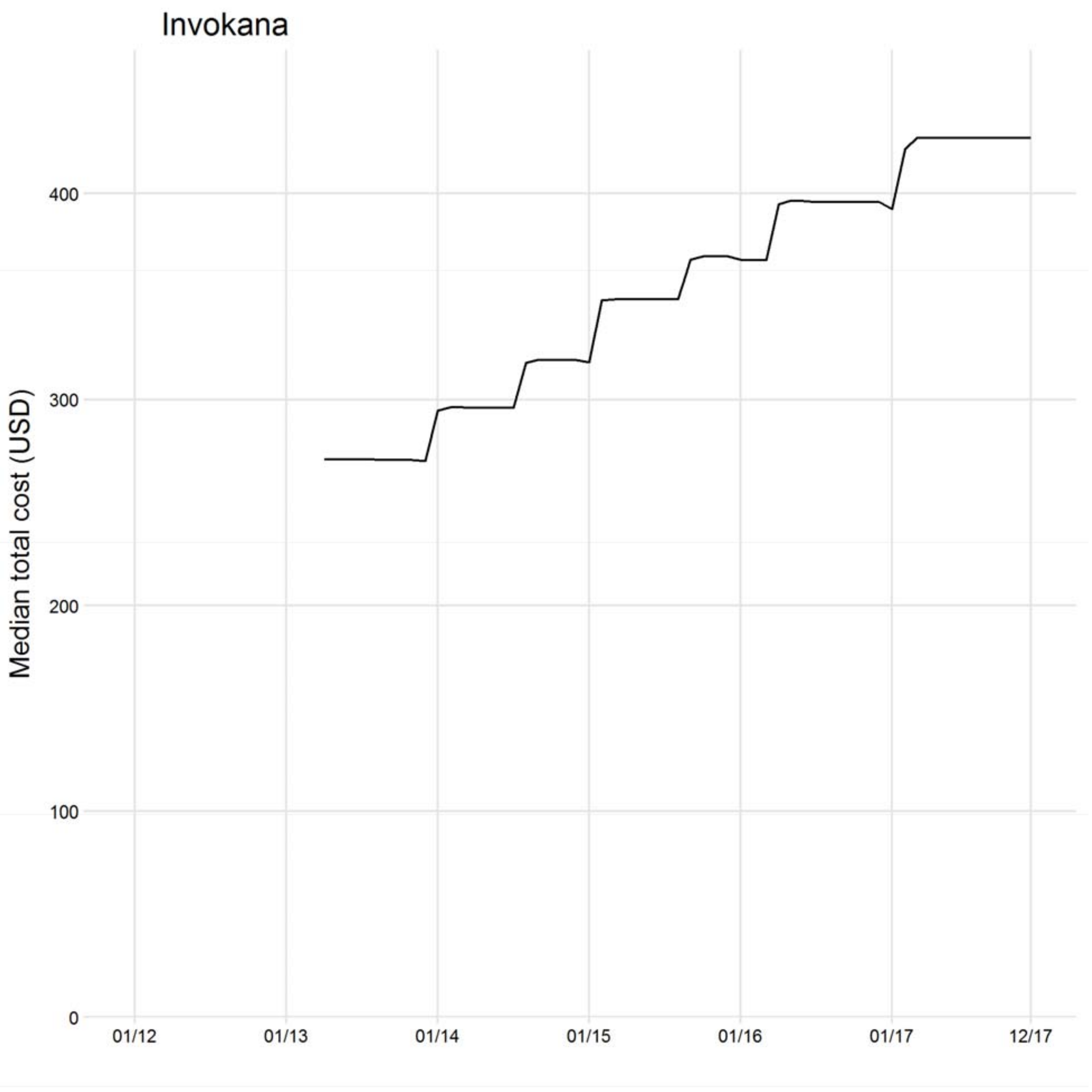

©2019 Wineinger NE et al. *JAMA Network Open*

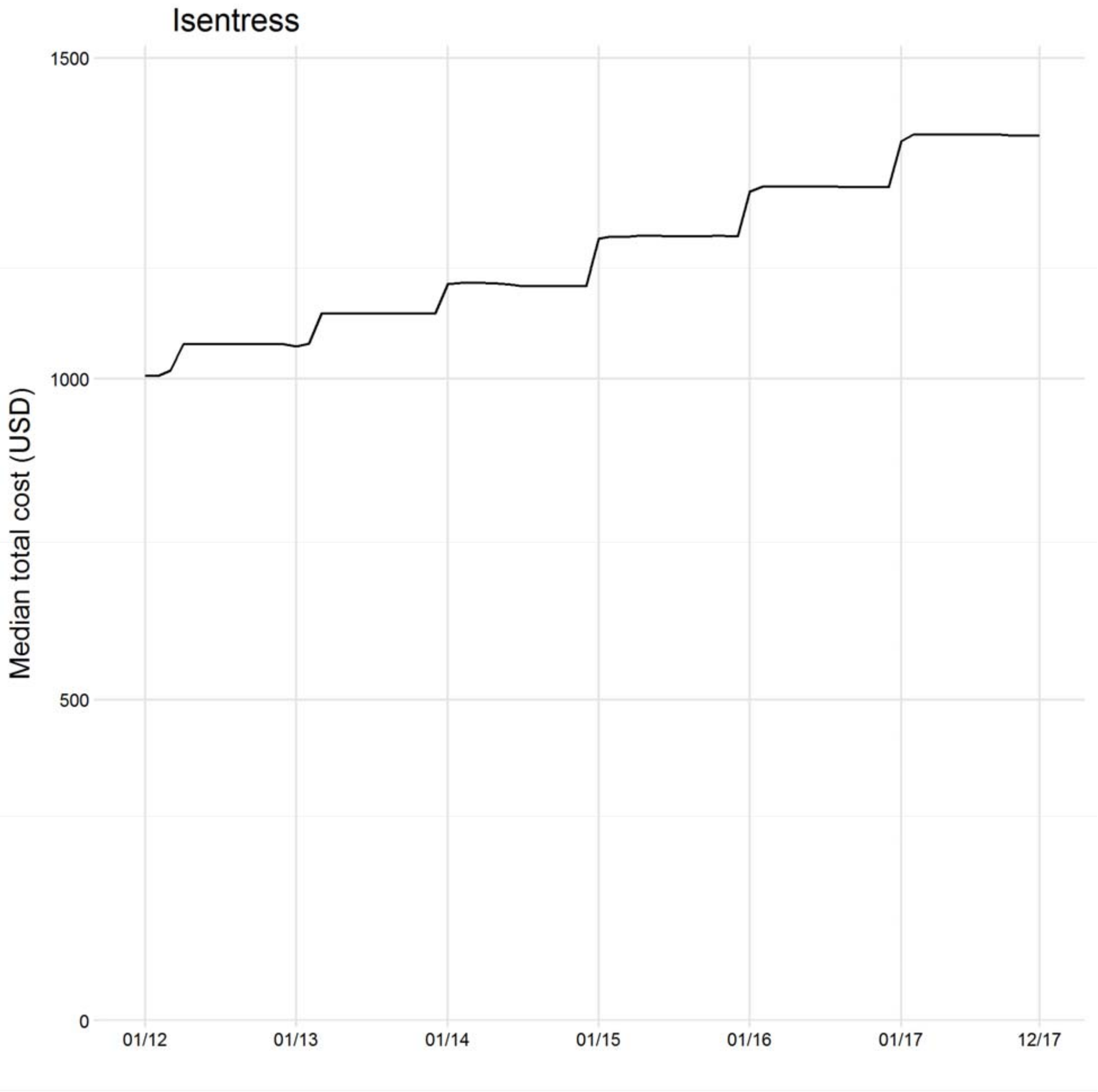

©2019 Wineinger NE et al. *JAMA Network Open*

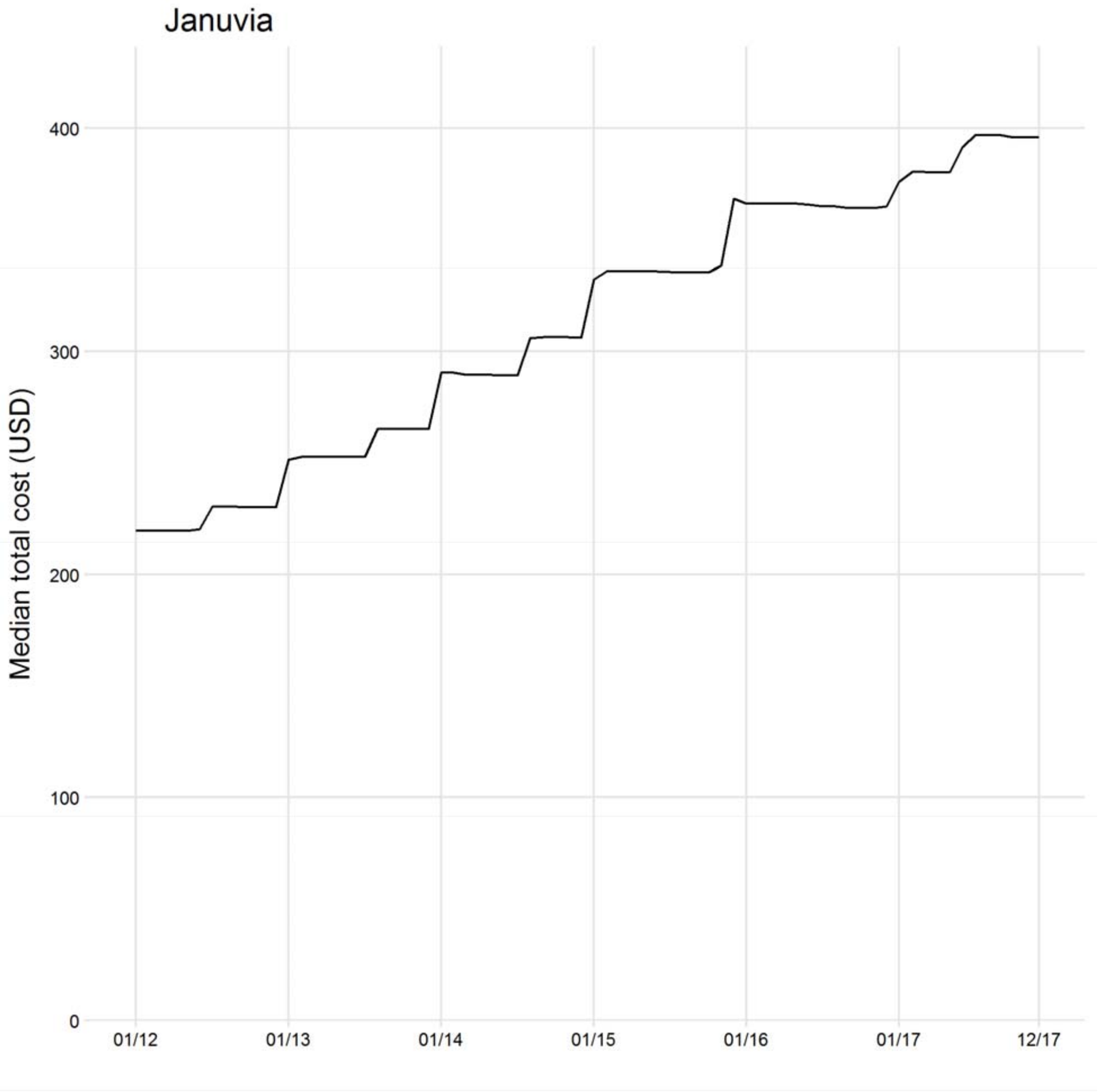

©2019 Wineinger NE et al. *JAMA Network Open*

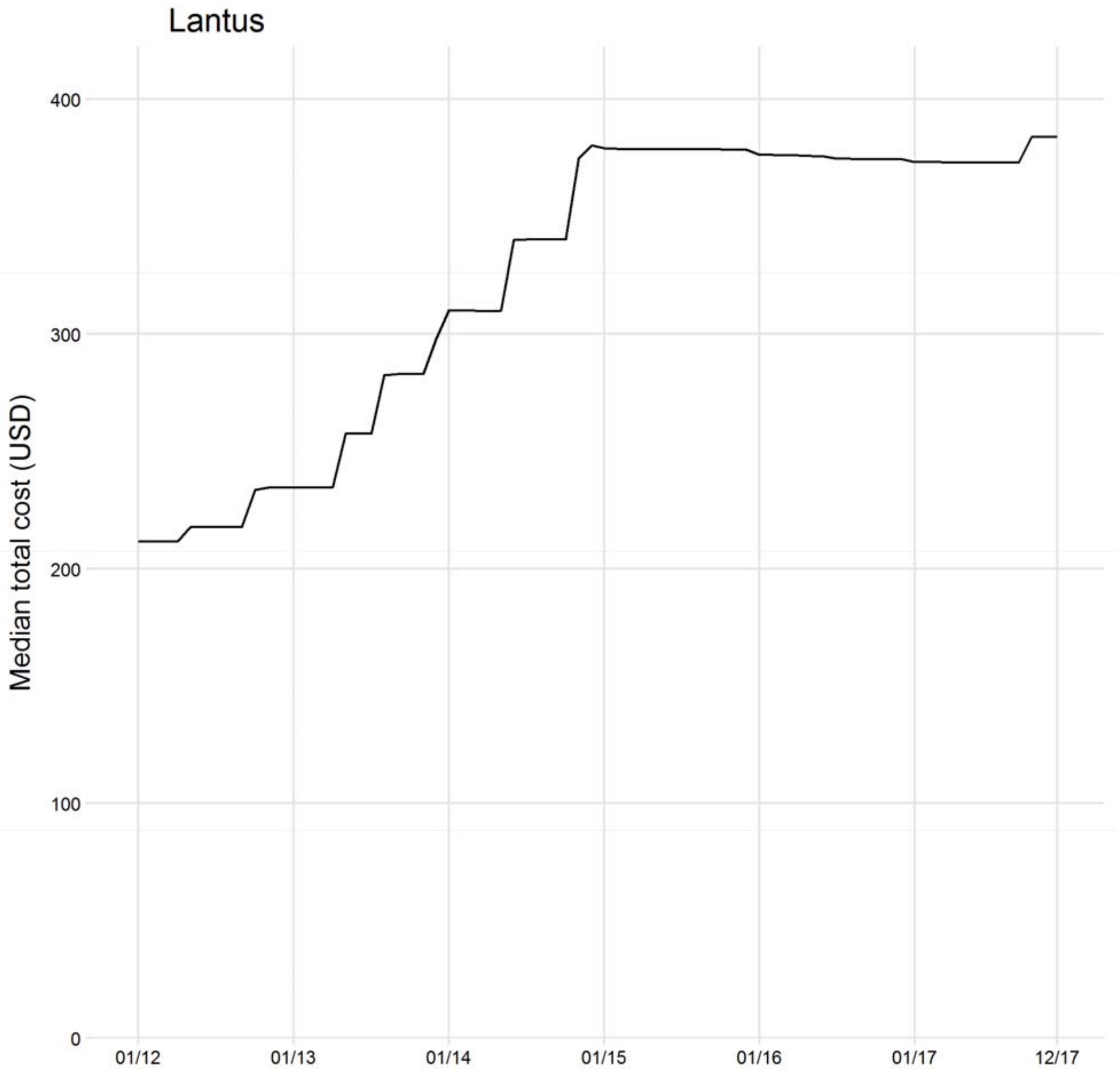

©2019 Wineinger NE et al. *JAMA Network Open*

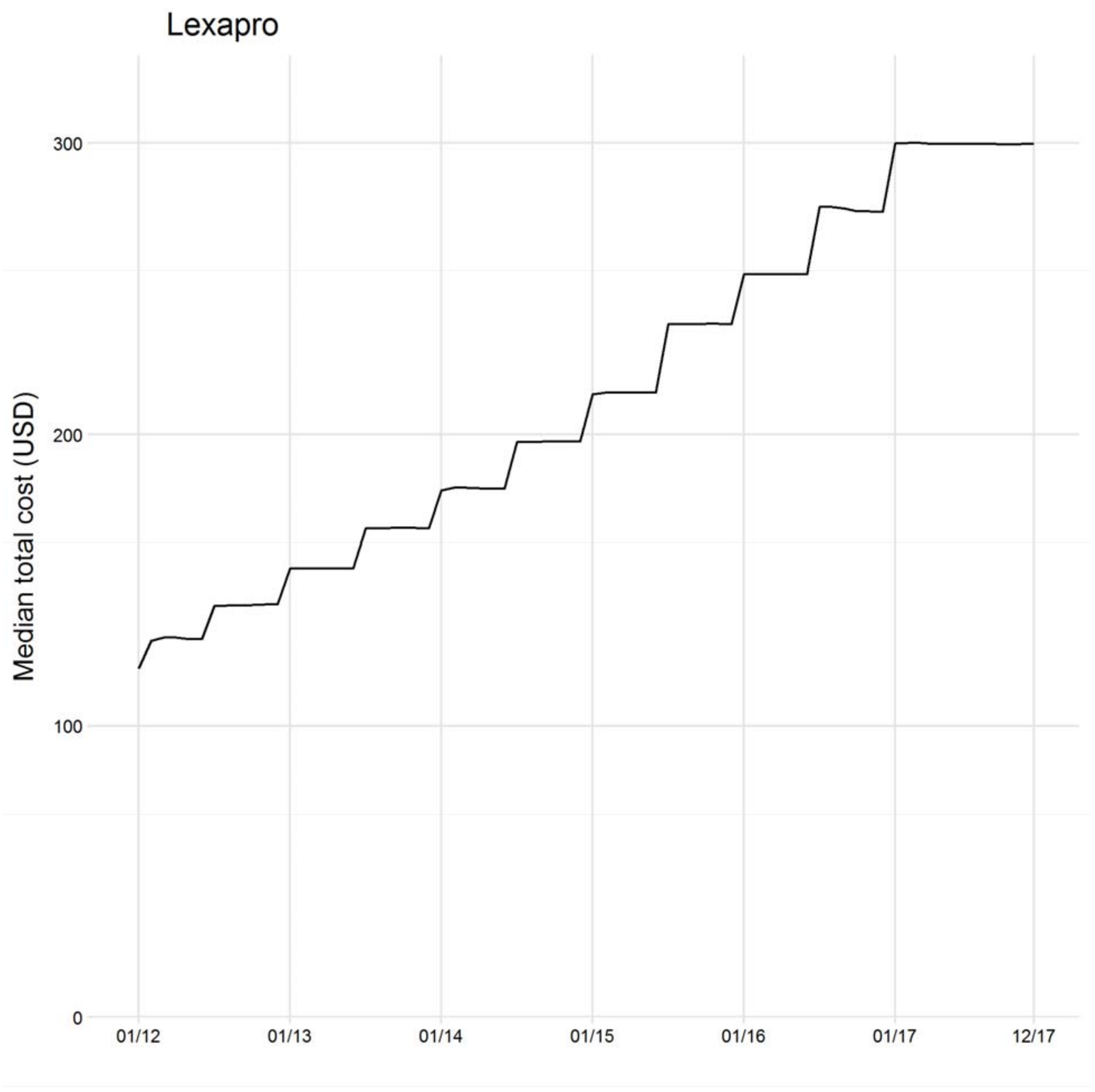

©2019 Wineinger NE et al. *JAMA Network Open*

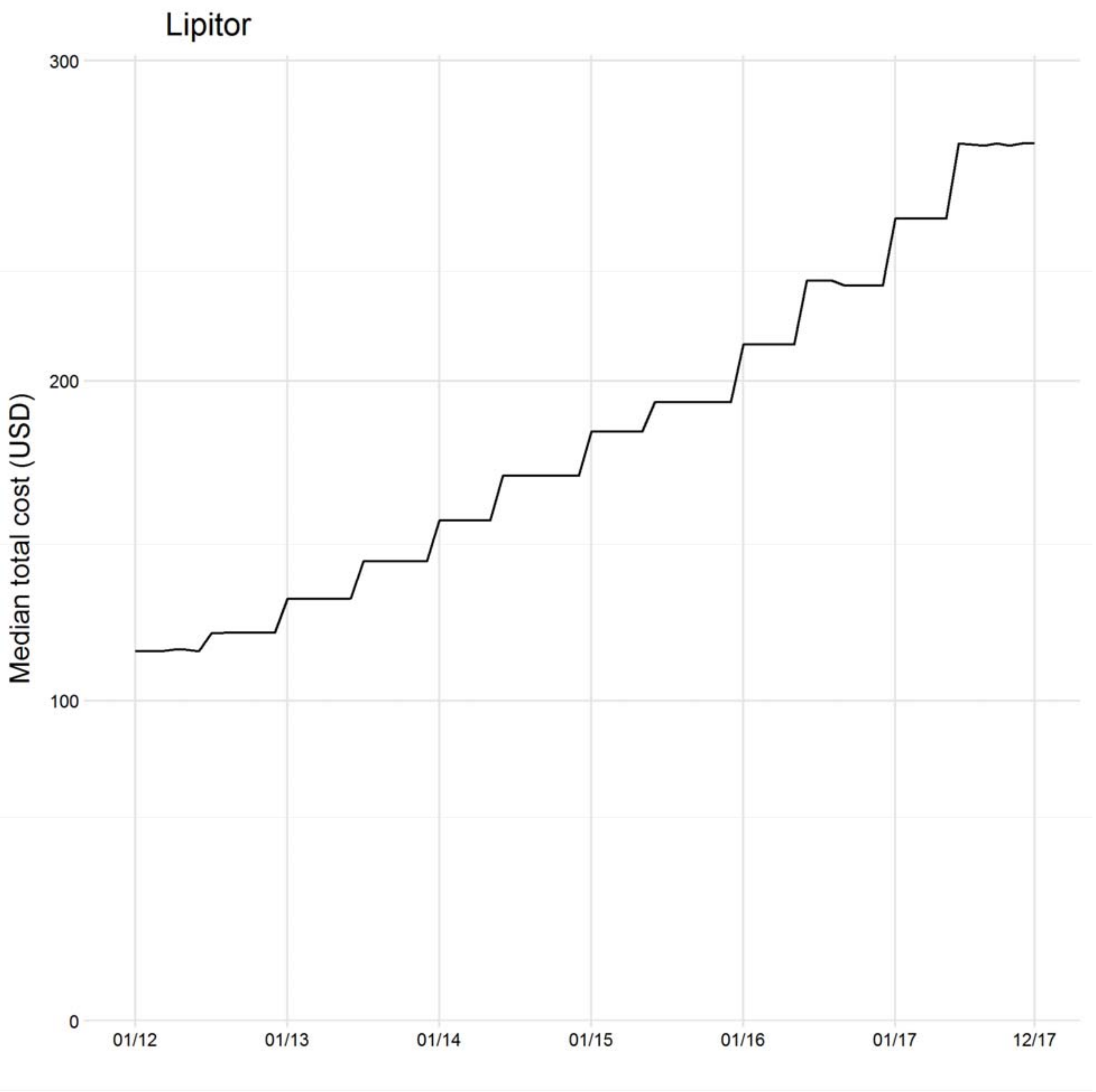

©2019 Wineinger NE et al. *JAMA Network Open*

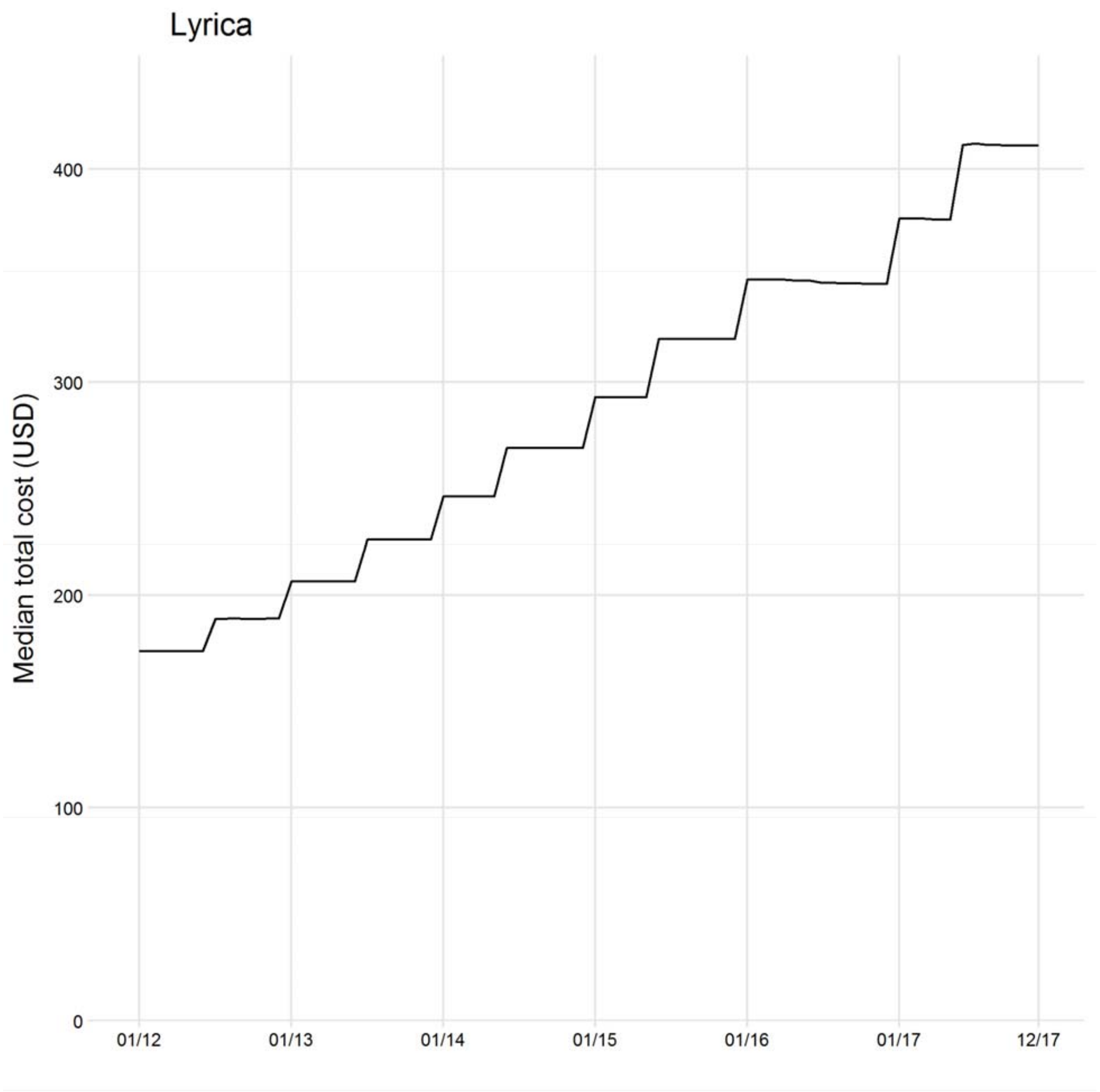

©2019 Wineinger NE et al. *JAMA Network Open*

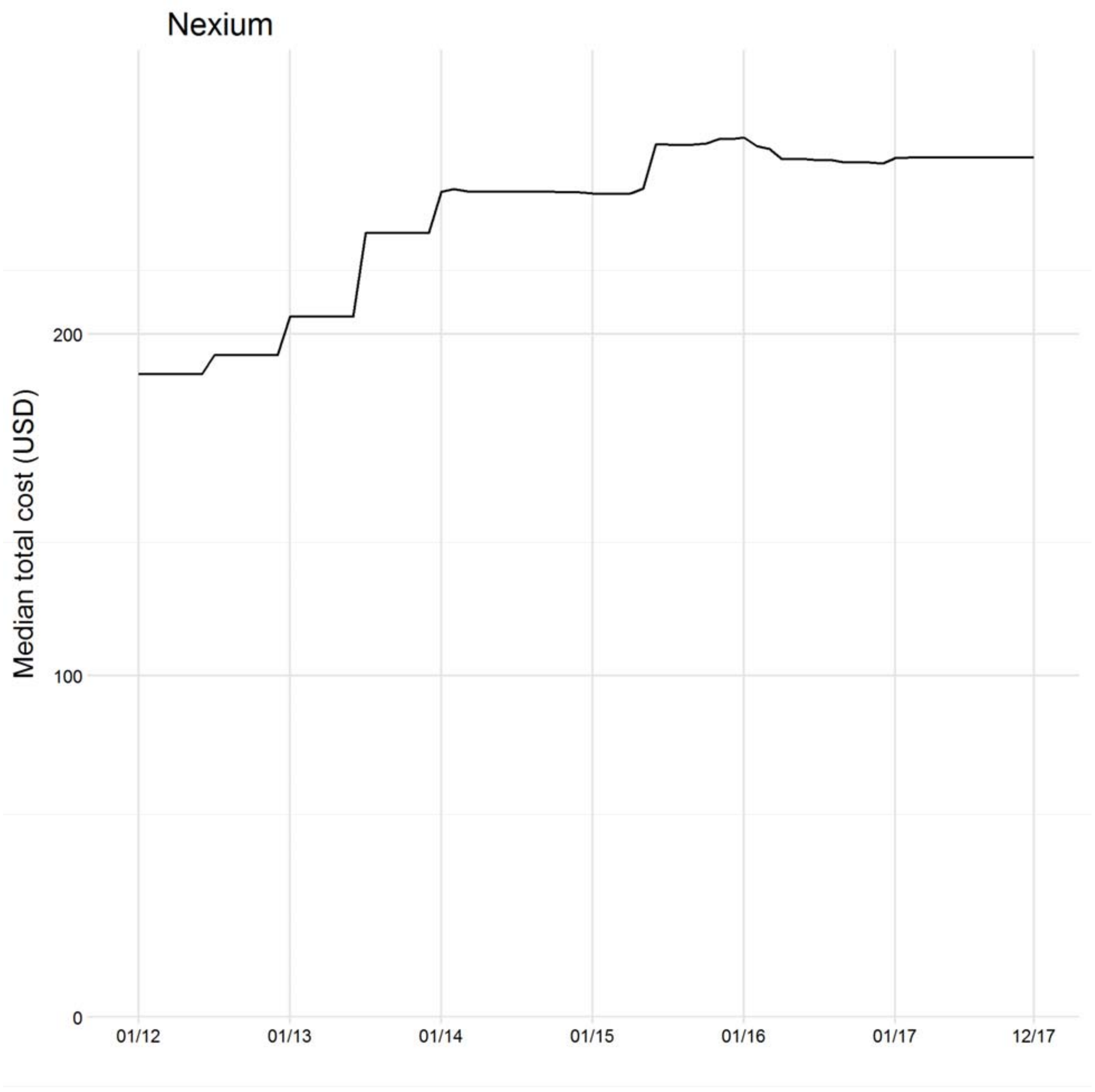

©2019 Wineinger NE et al. *JAMA Network Open*

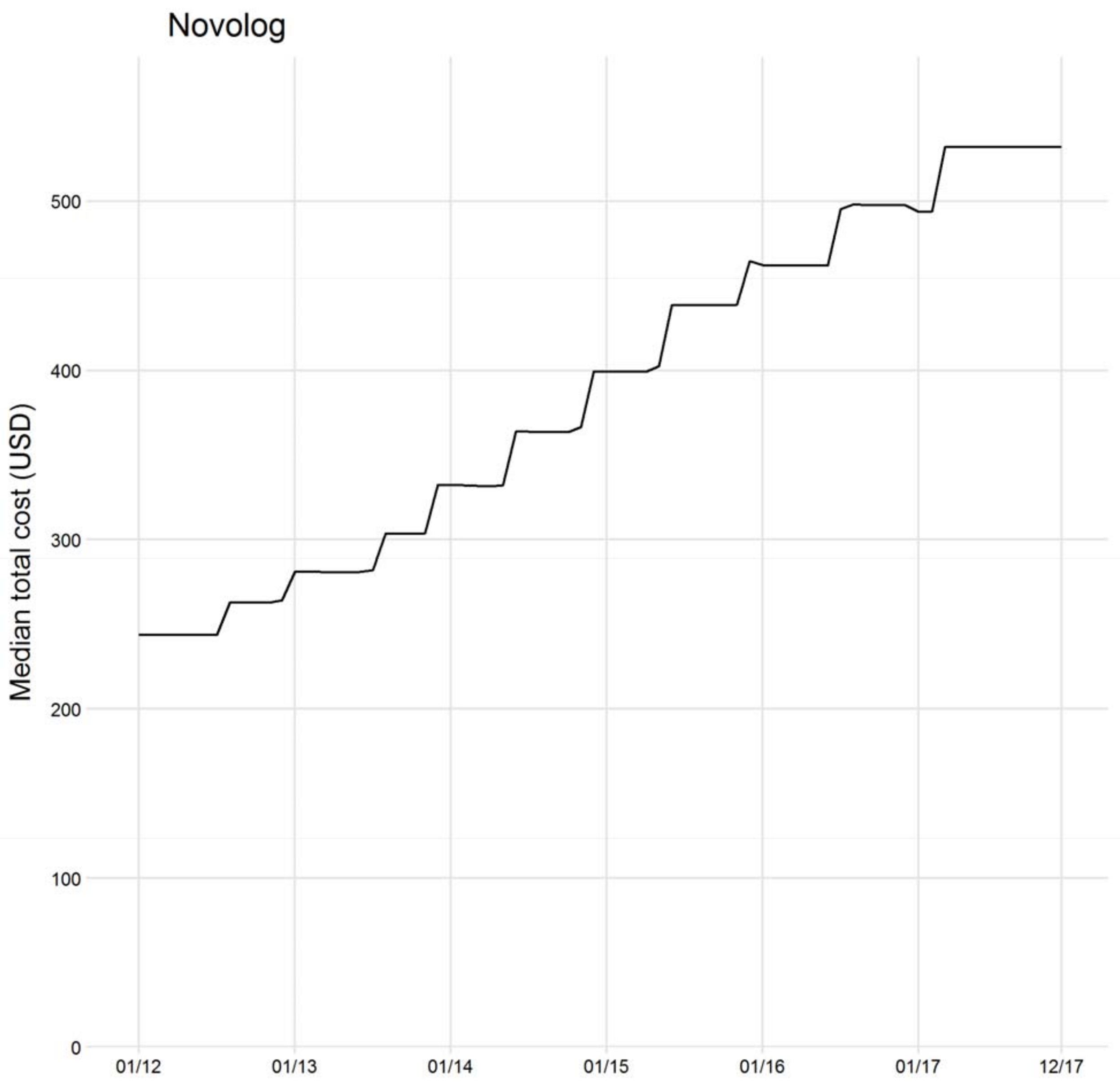

©2019 Wineinger NE et al. *JAMA Network Open*

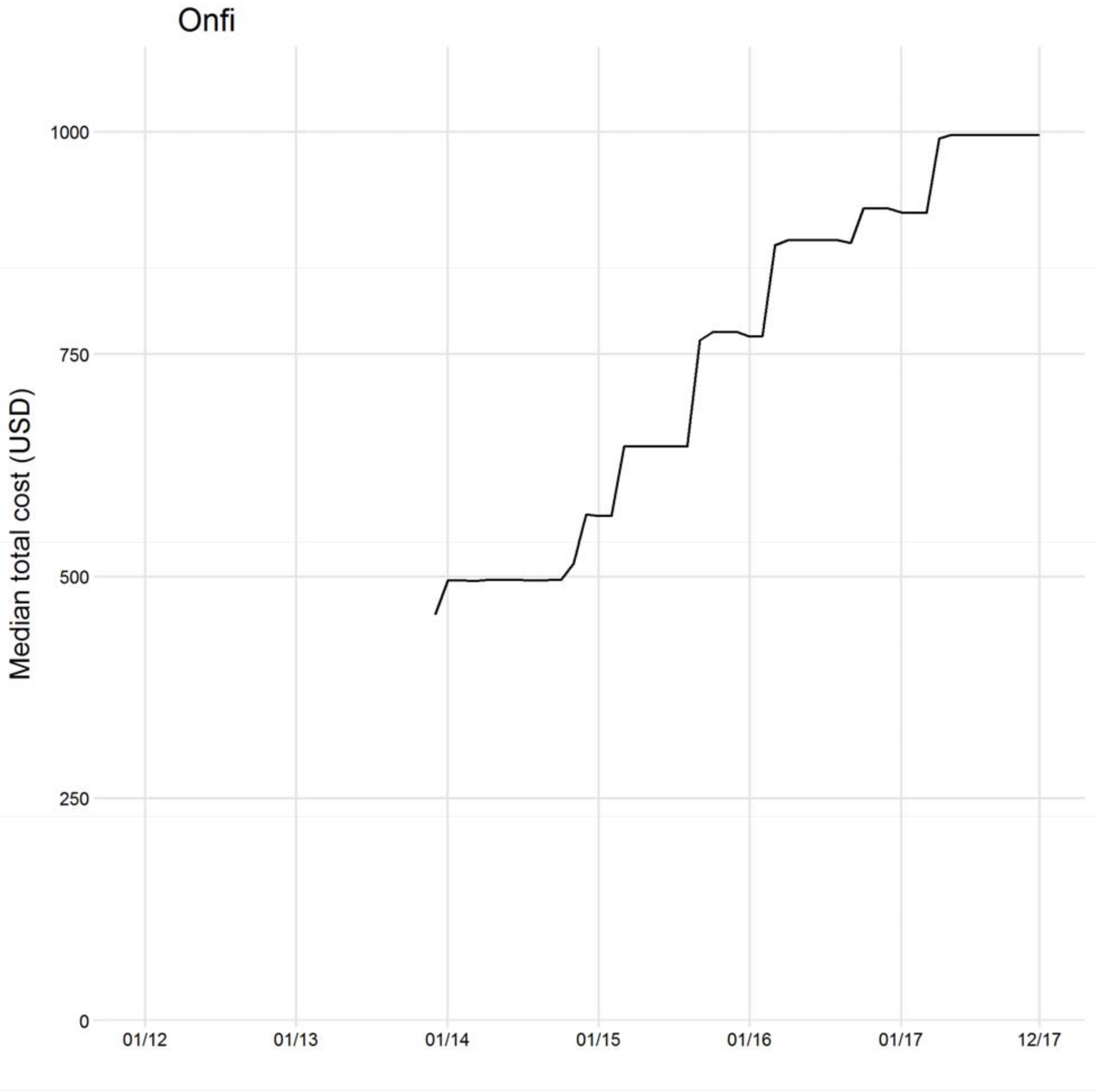

©2019 Wineinger NE et al. *JAMA Network Open*

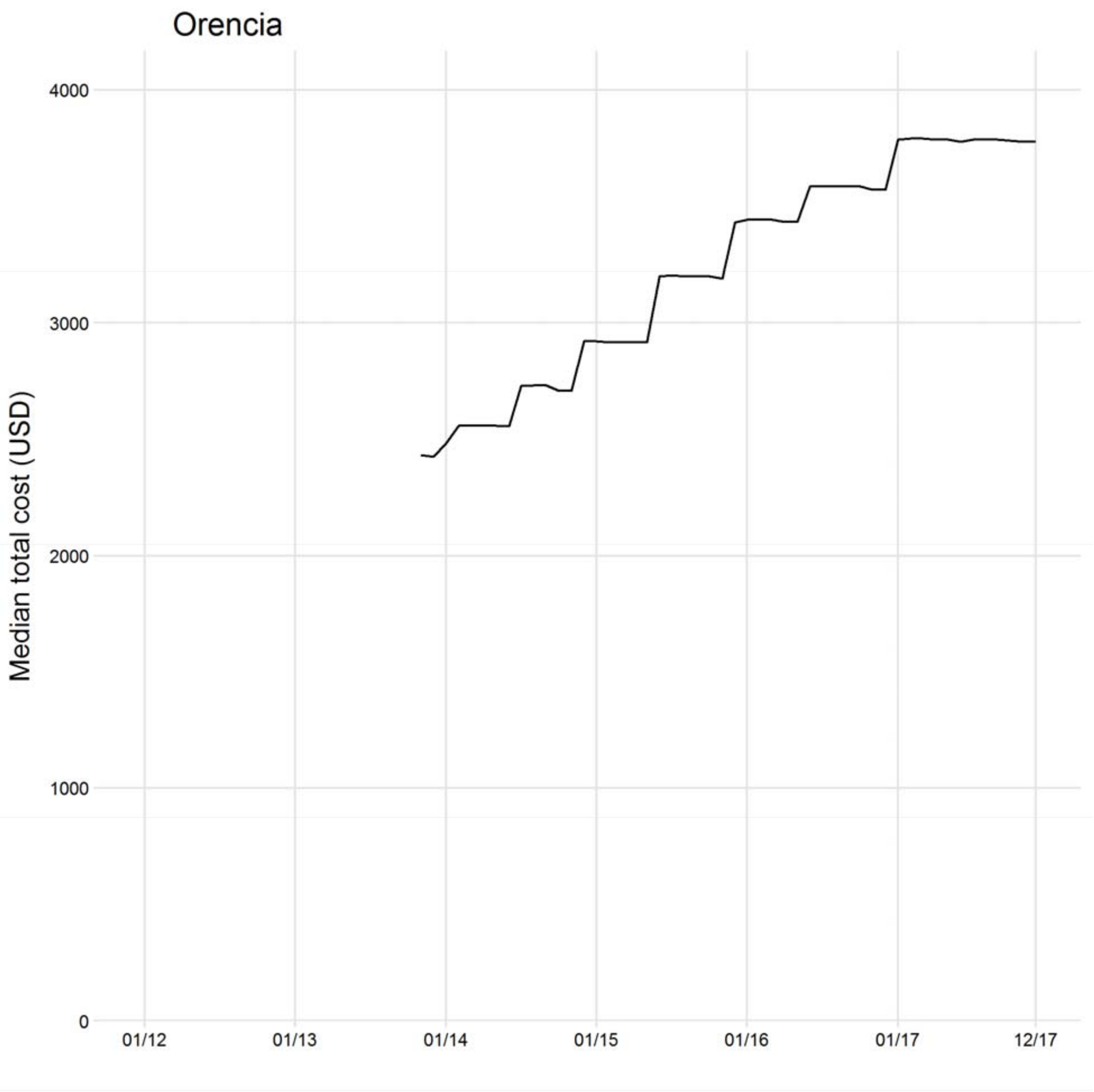

©2019 Wineinger NE et al. *JAMA Network Open*

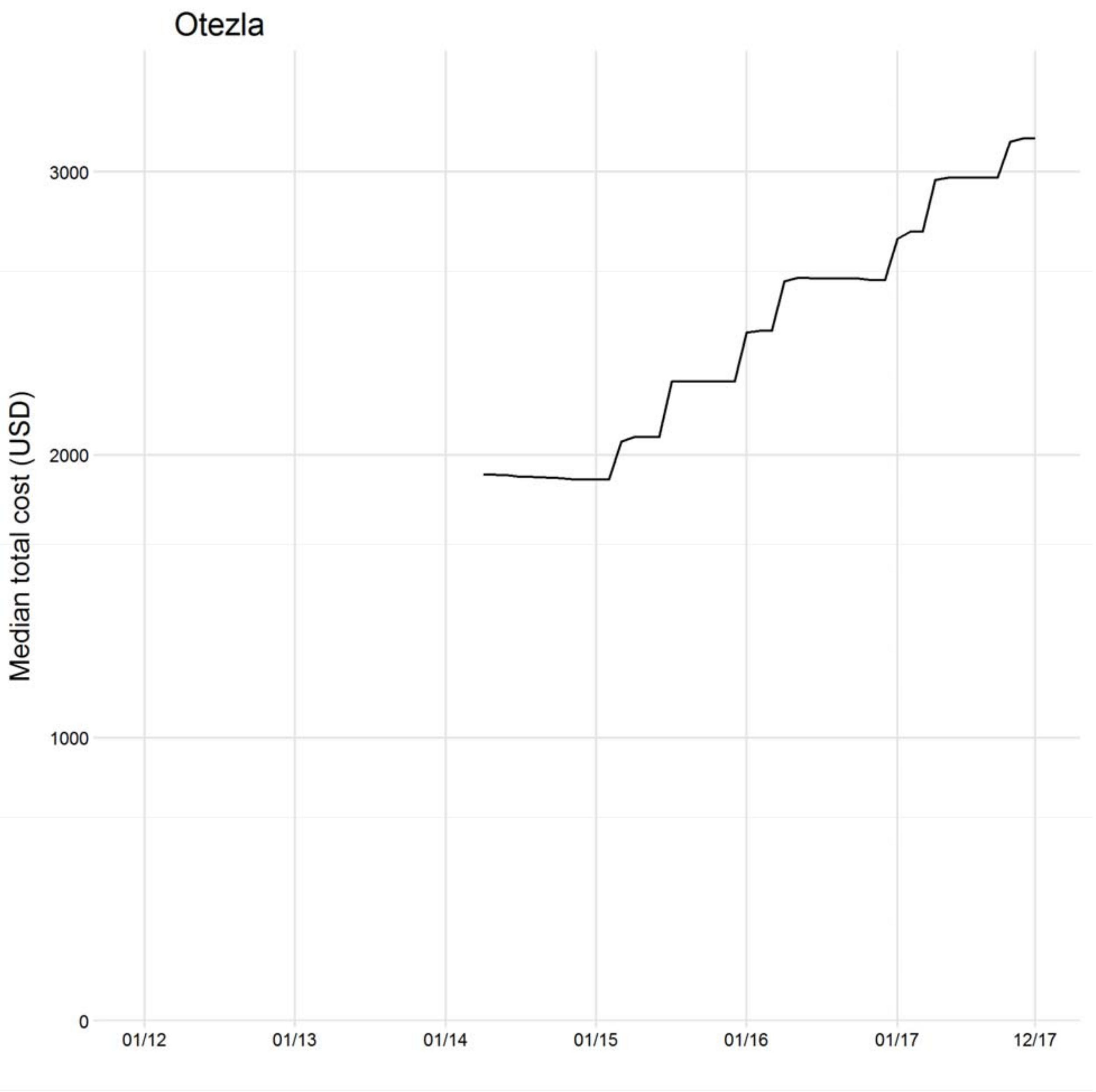

©2019 Wineinger NE et al. *JAMA Network Open*

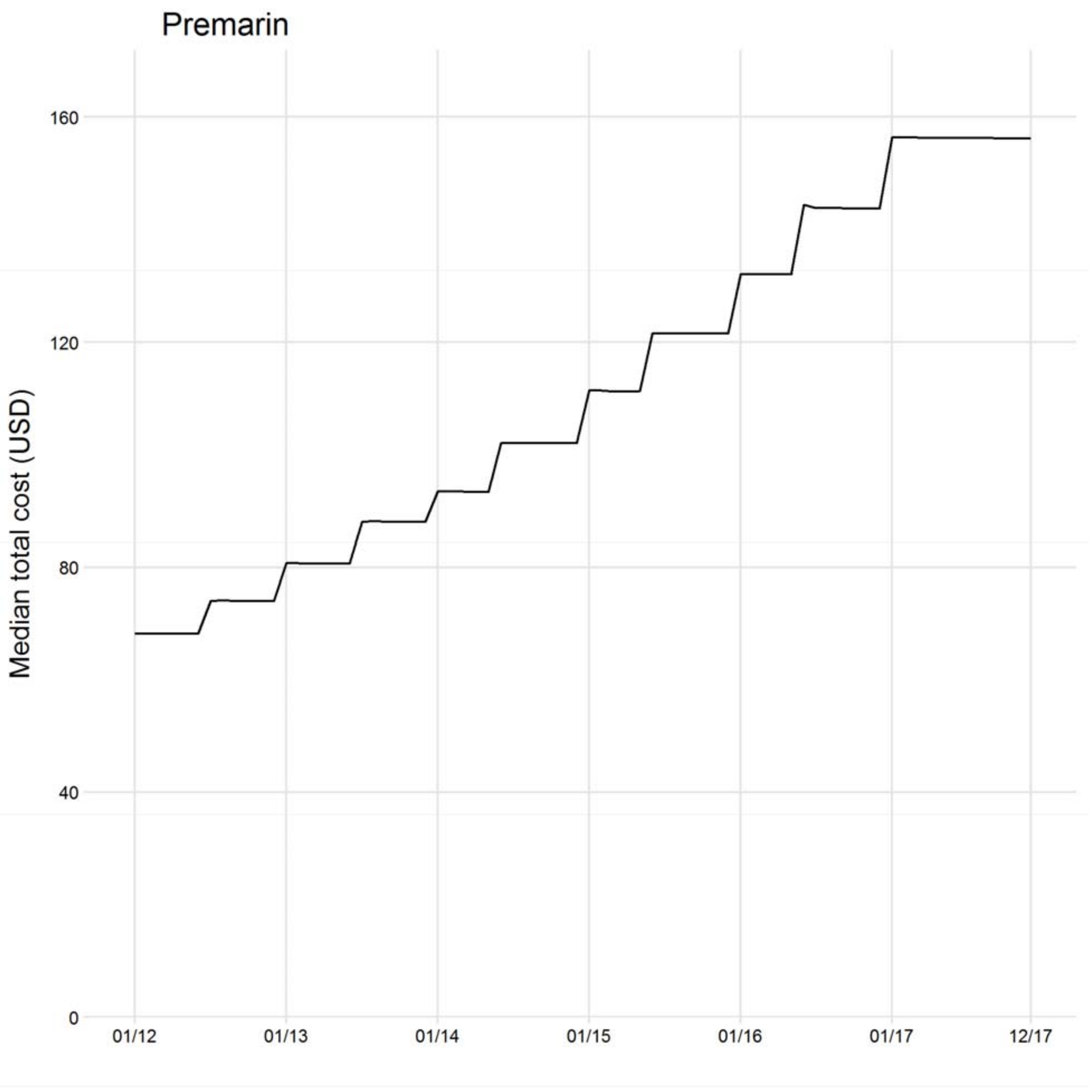

©2019 Wineinger NE et al. *JAMA Network Open*

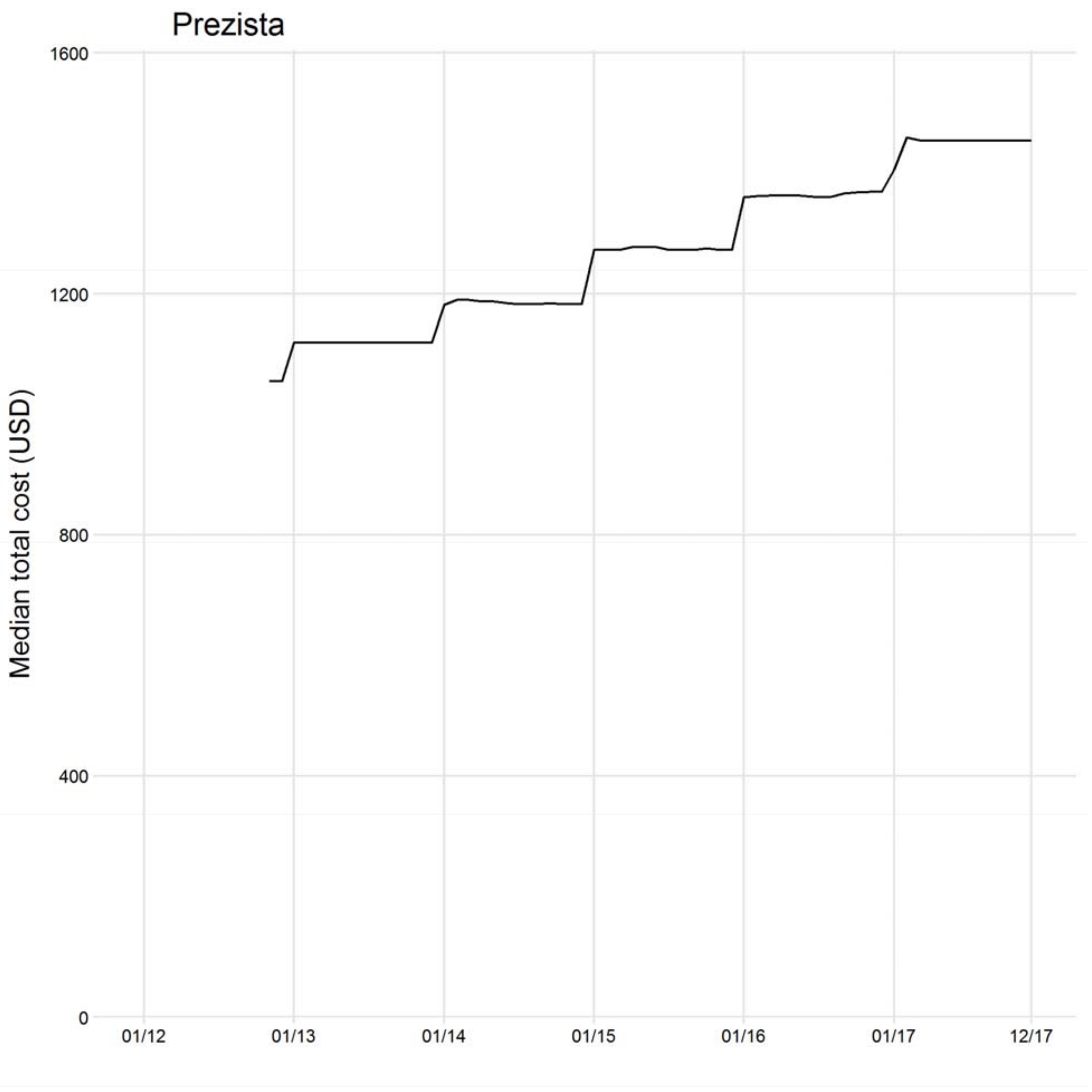

©2019 Wineinger NE et al. *JAMA Network Open*

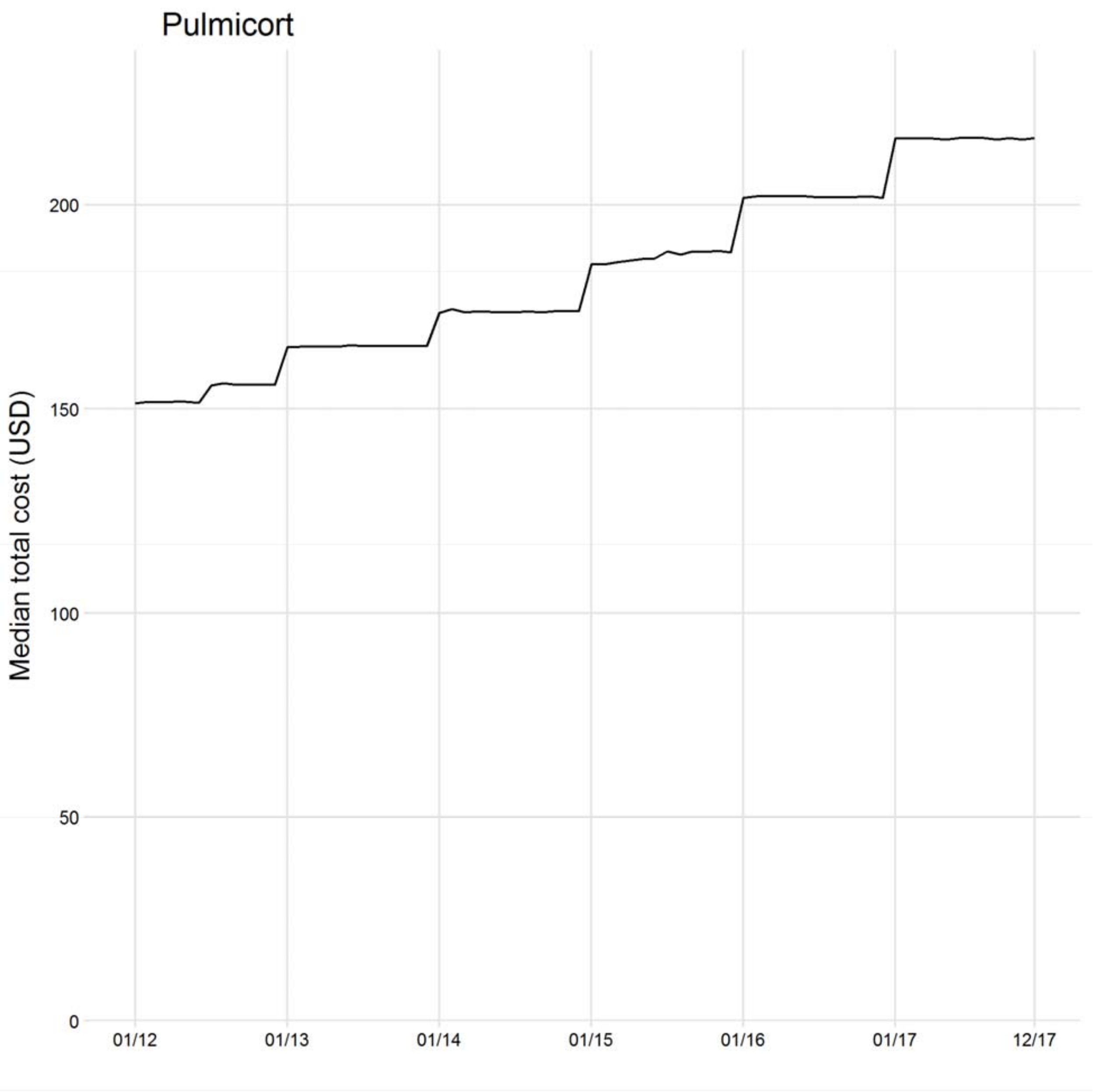

©2019 Wineinger NE et al. *JAMA Network Open*

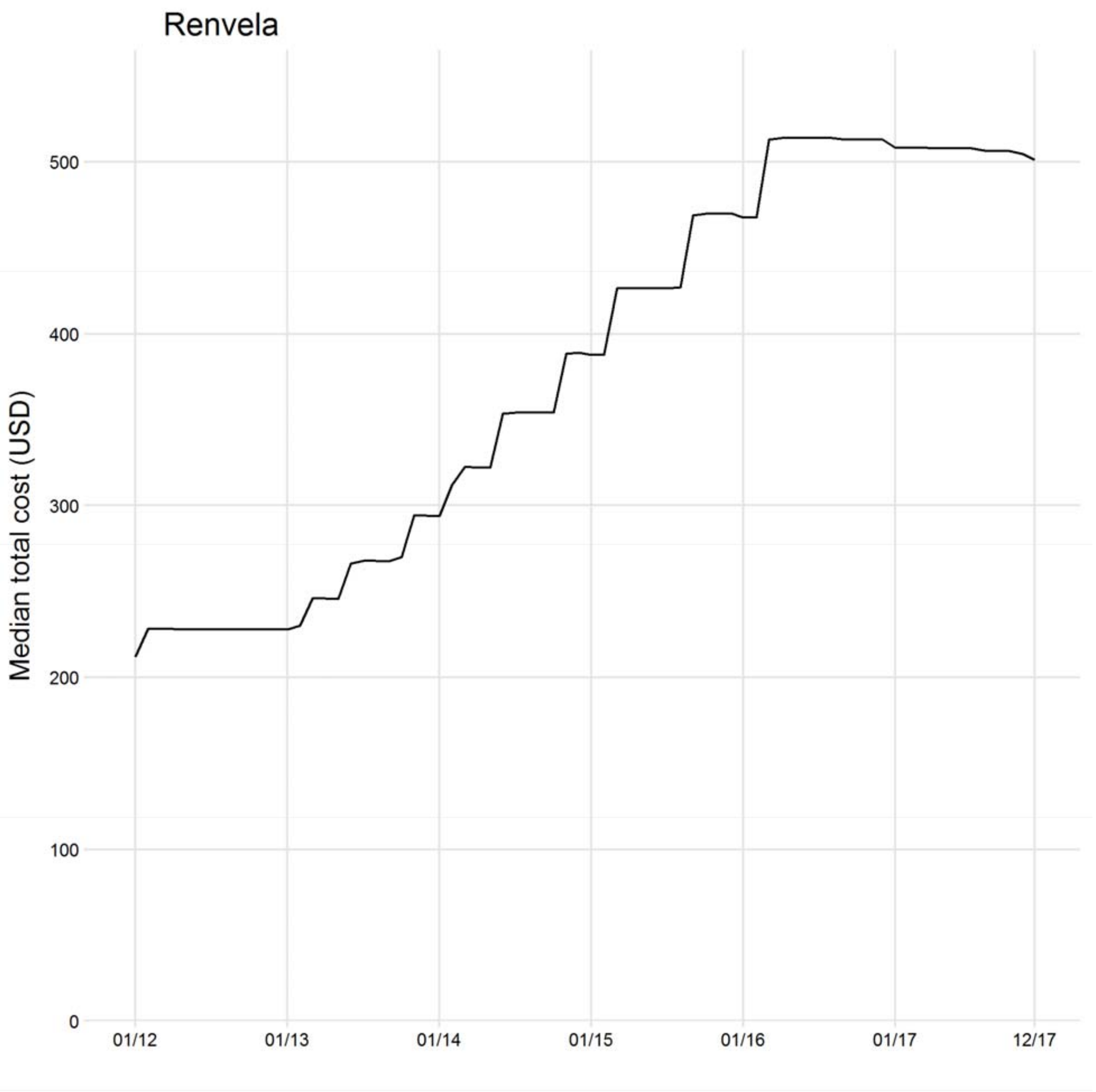

©2019 Wineinger NE et al. *JAMA Network Open*

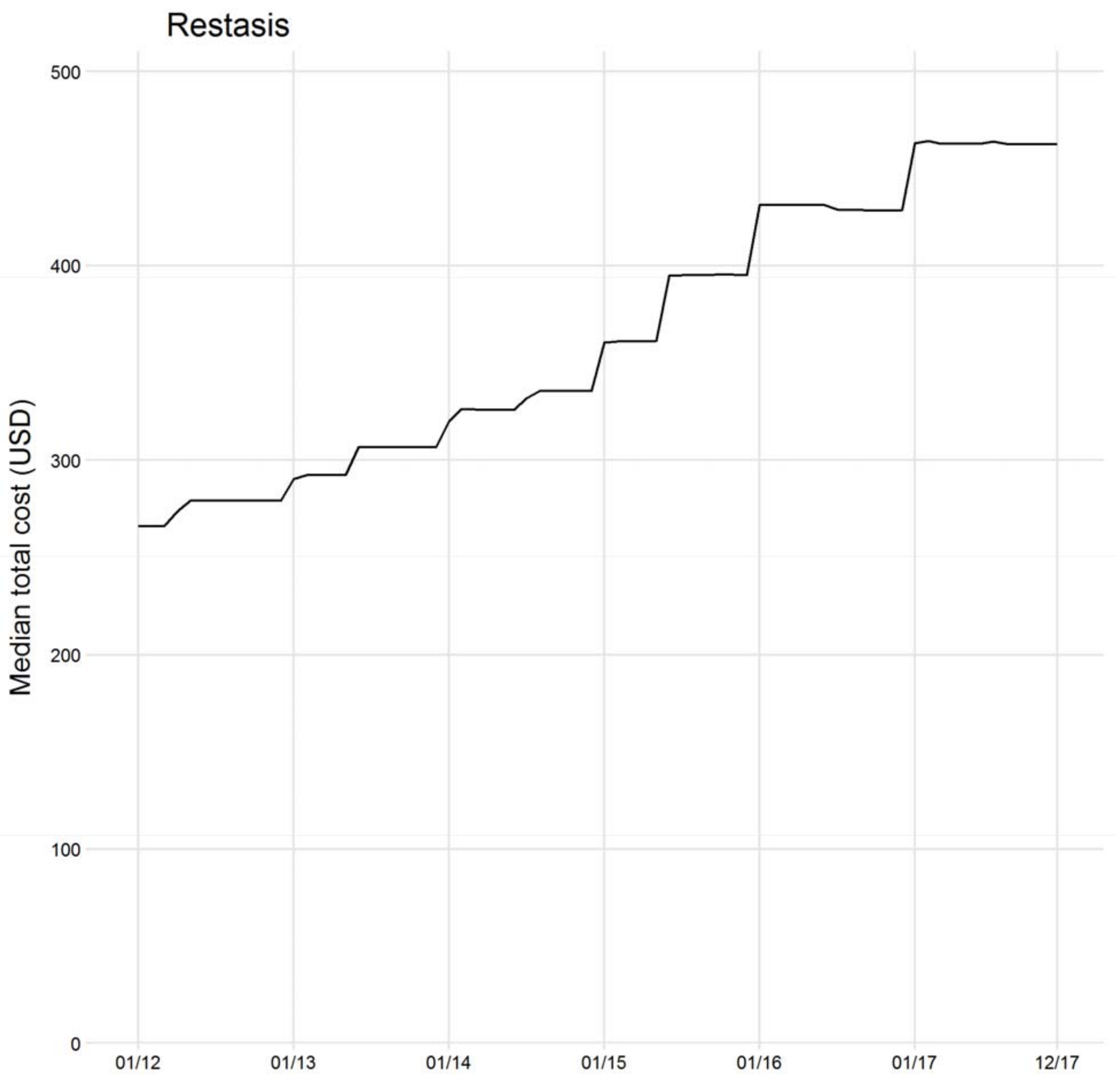

©2019 Wineinger NE et al. *JAMA Network Open*

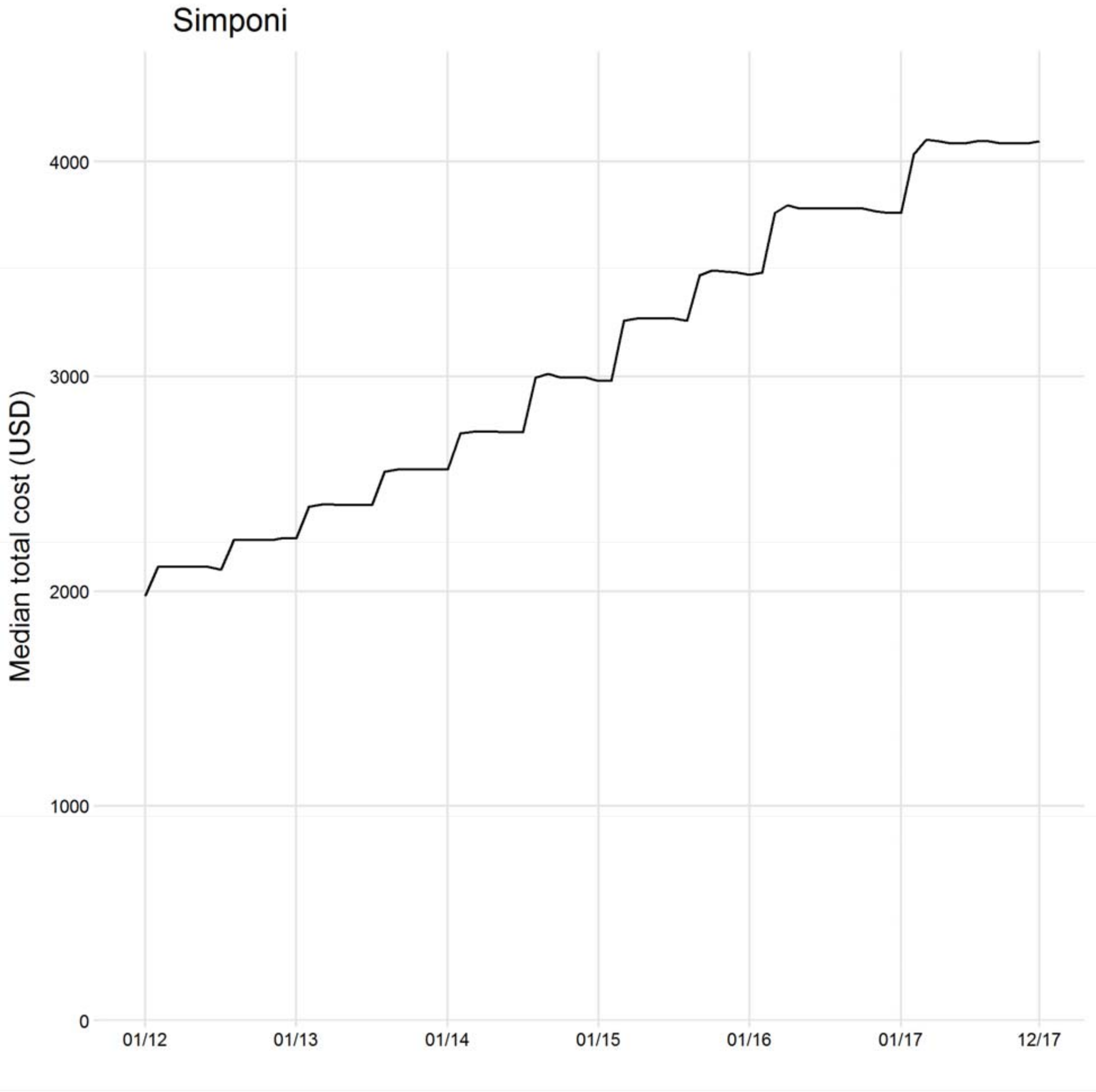

©2019 Wineinger NE et al. *JAMA Network Open*

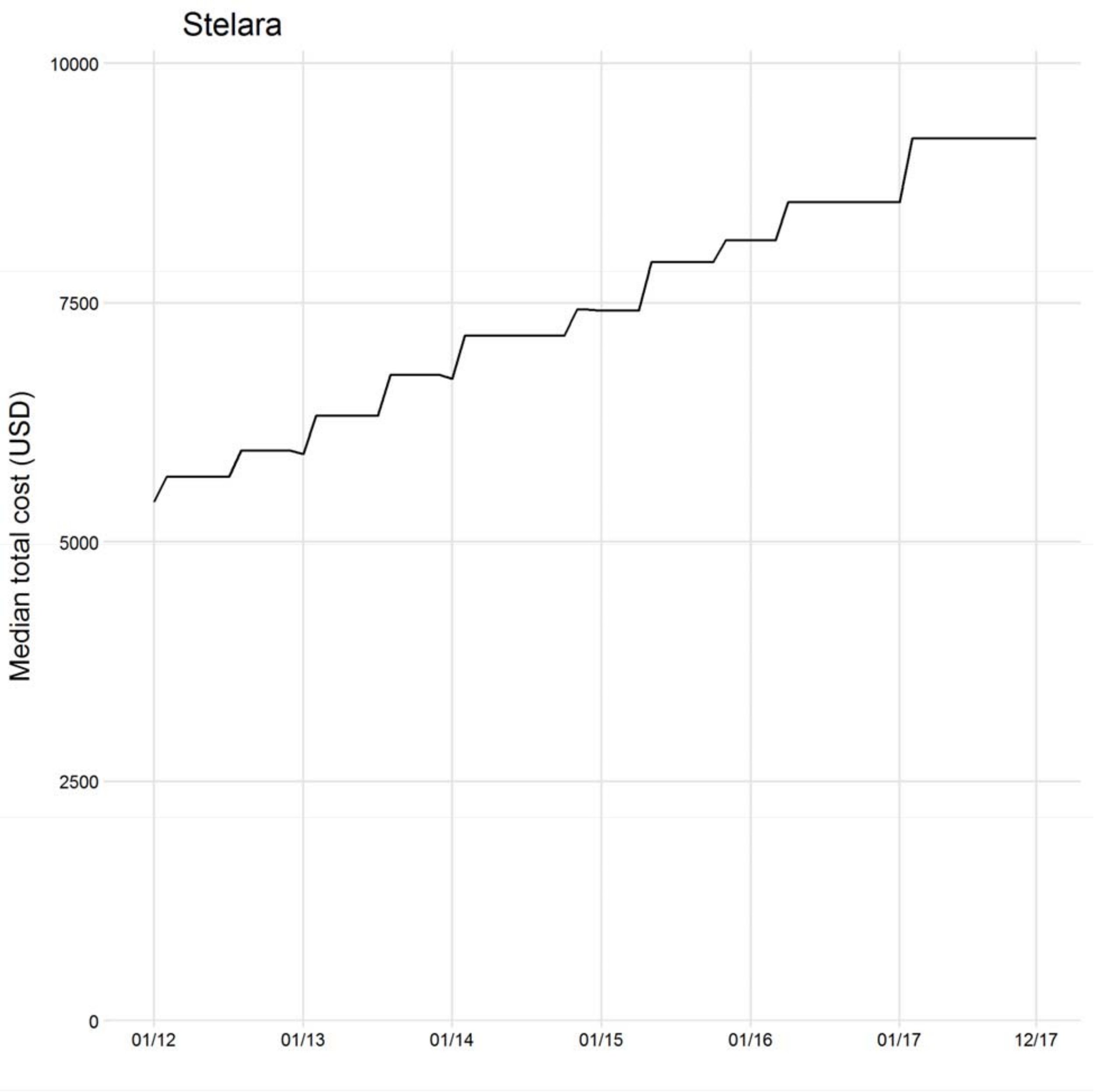

©2019 Wineinger NE et al. *JAMA Network Open*

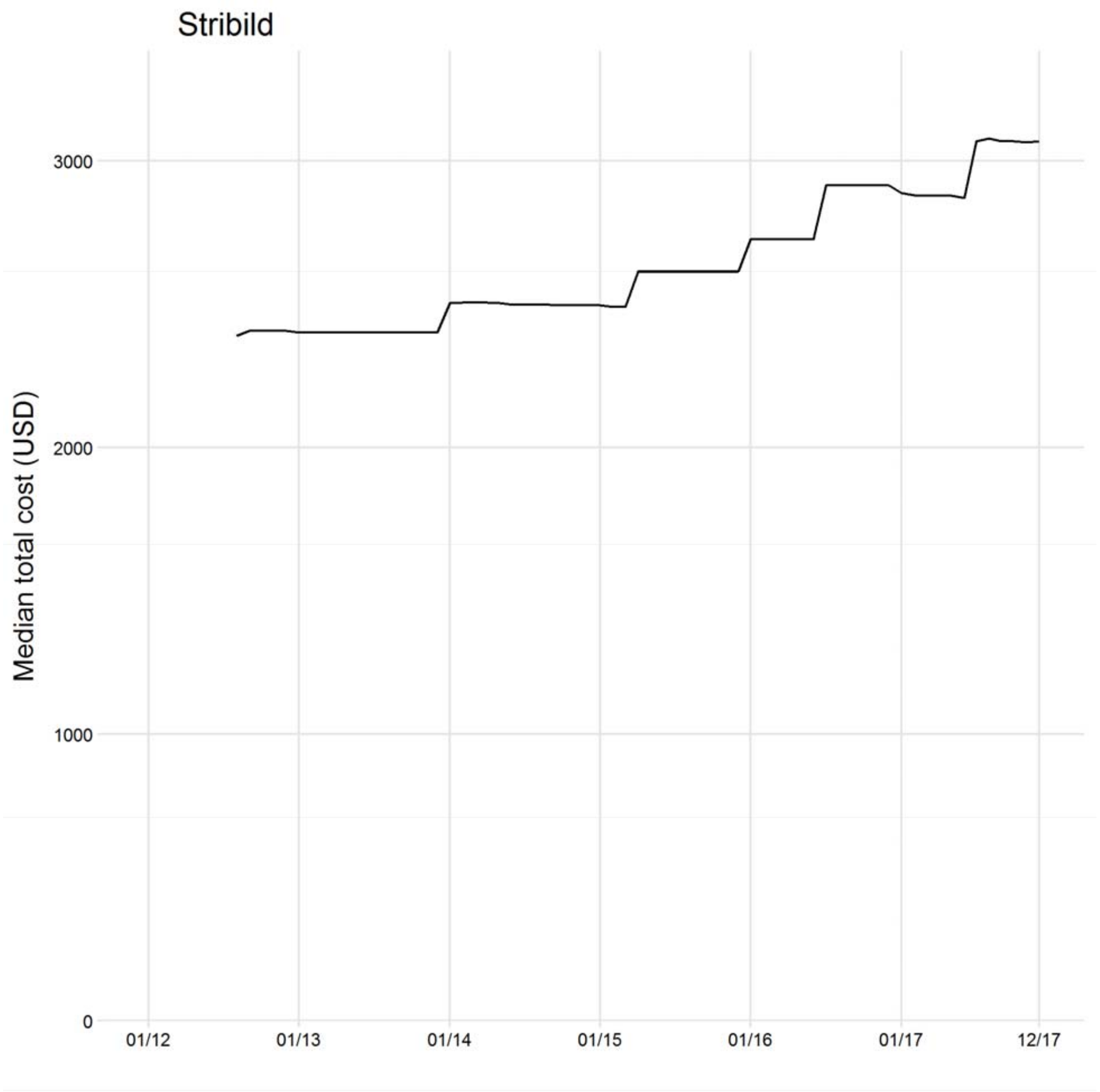

©2019 Wineinger NE et al. *JAMA Network Open*

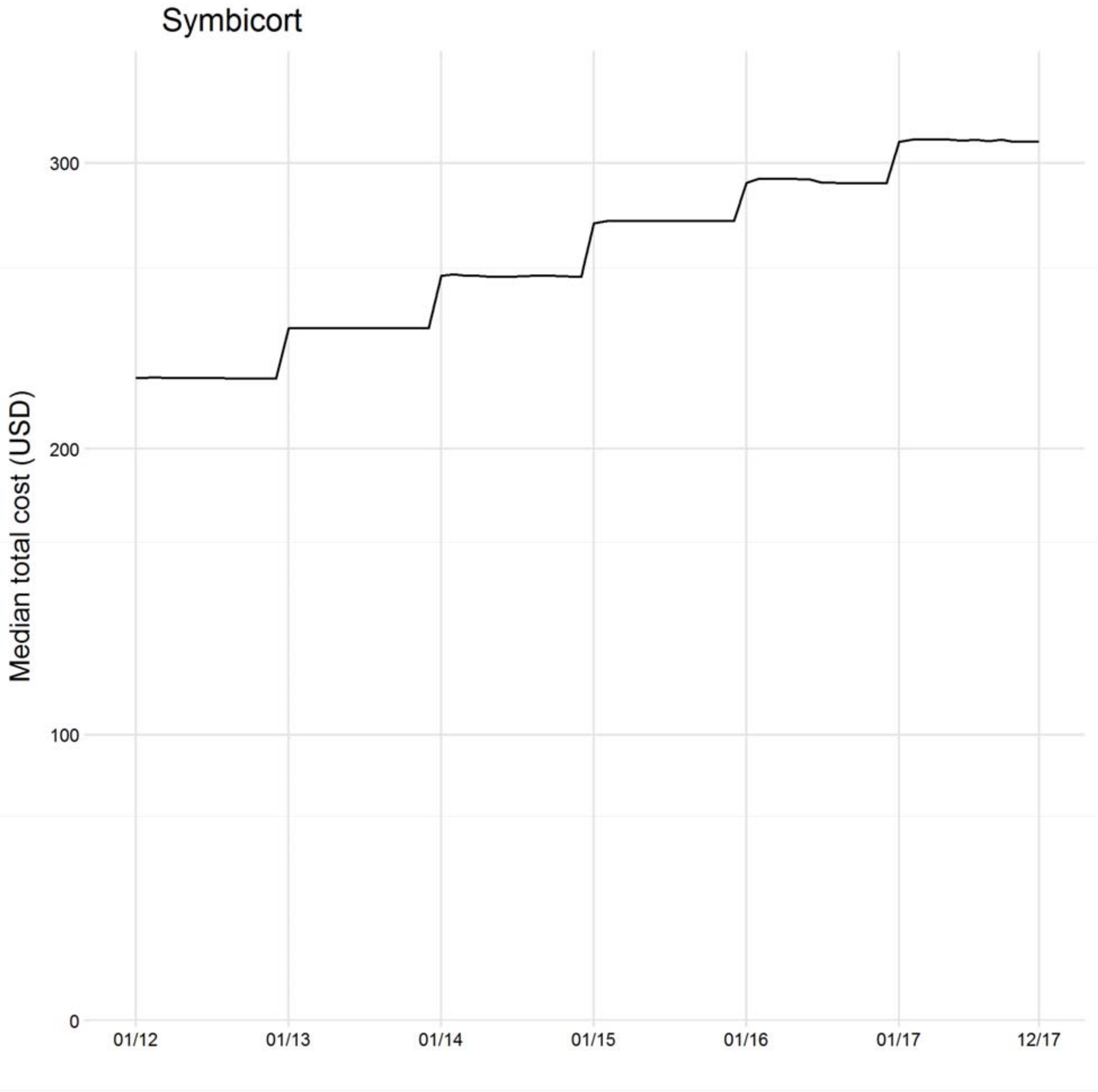

©2019 Wineinger NE et al. *JAMA Network Open*

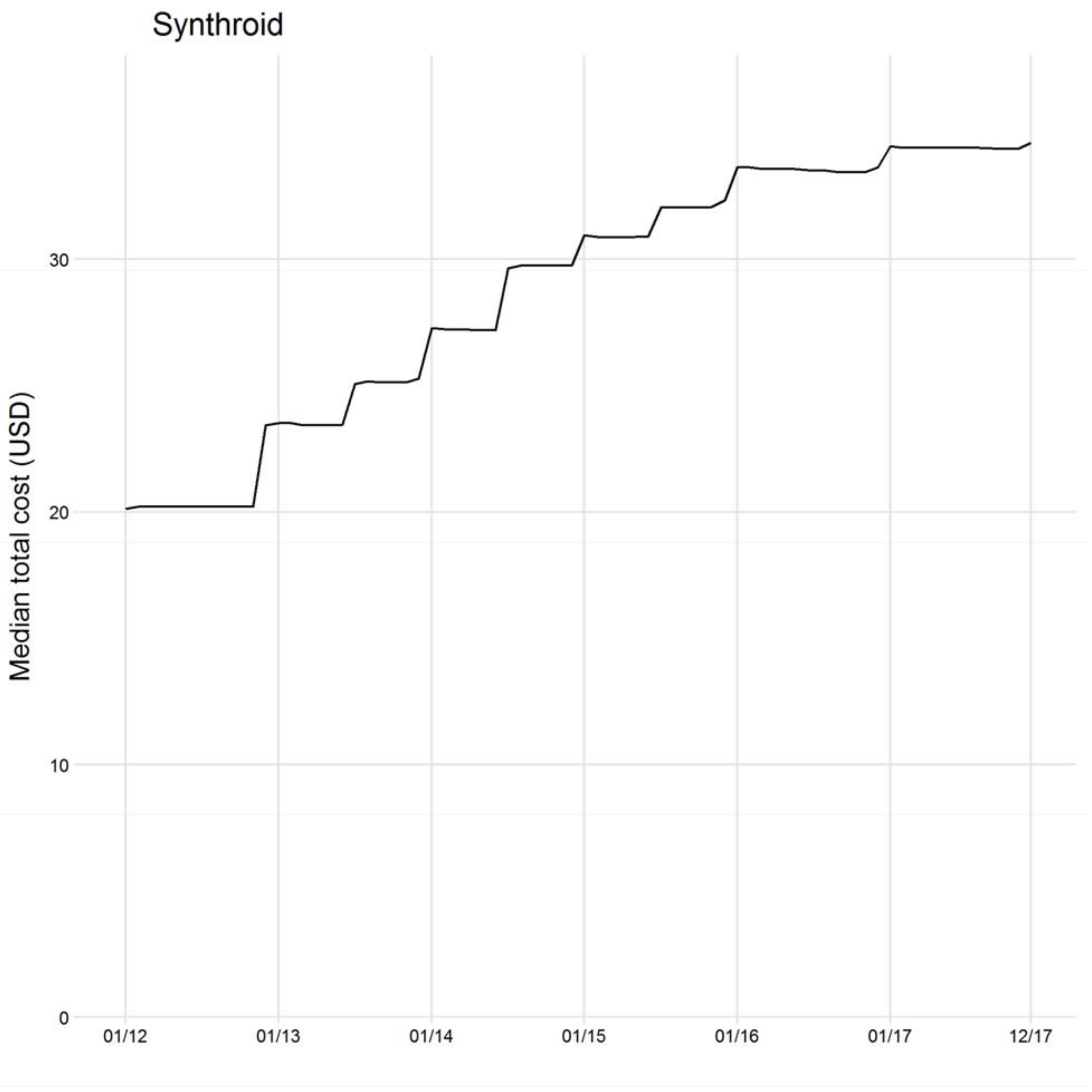

©2019 Wineinger NE et al. *JAMA Network Open*

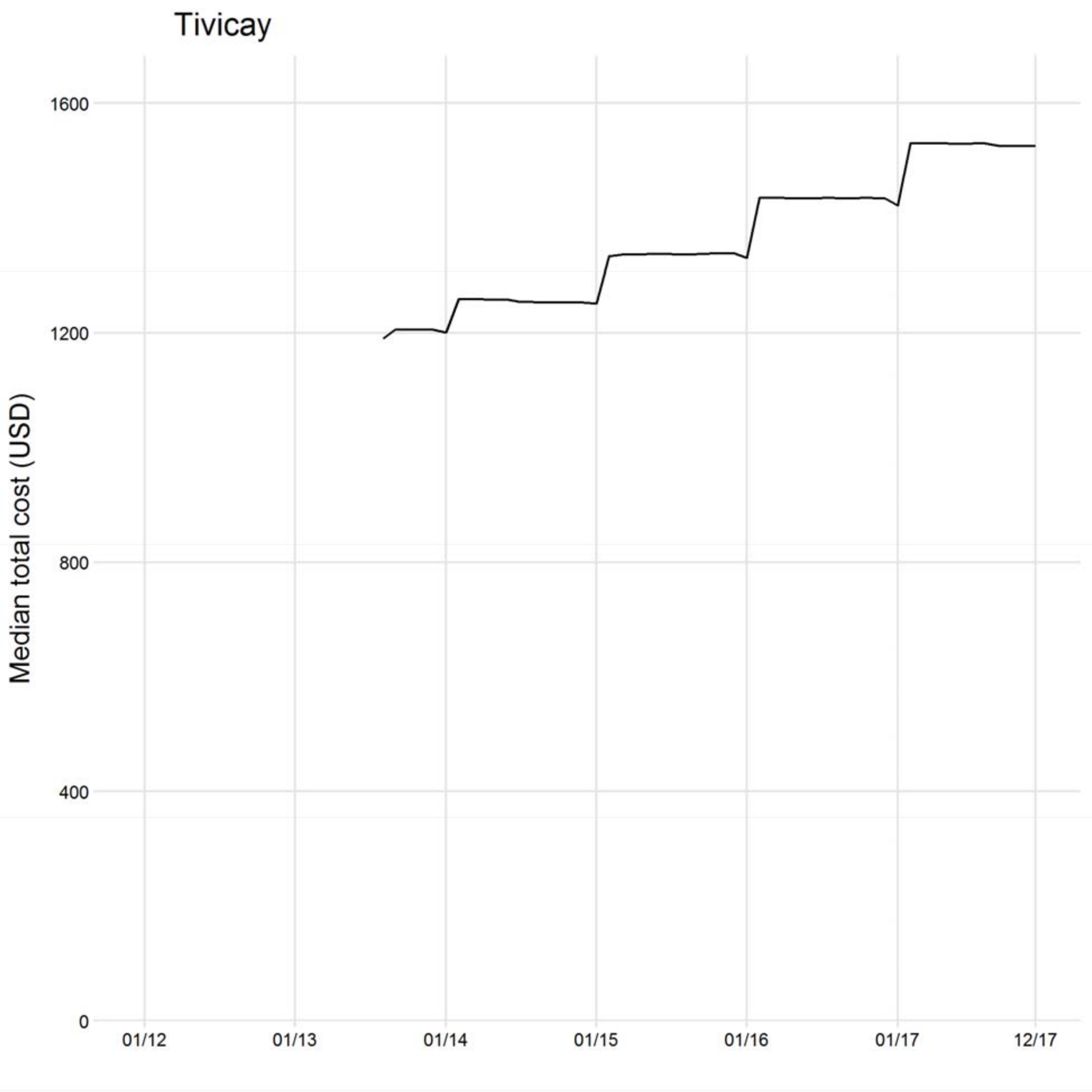

©2019 Wineinger NE et al. *JAMA Network Open*

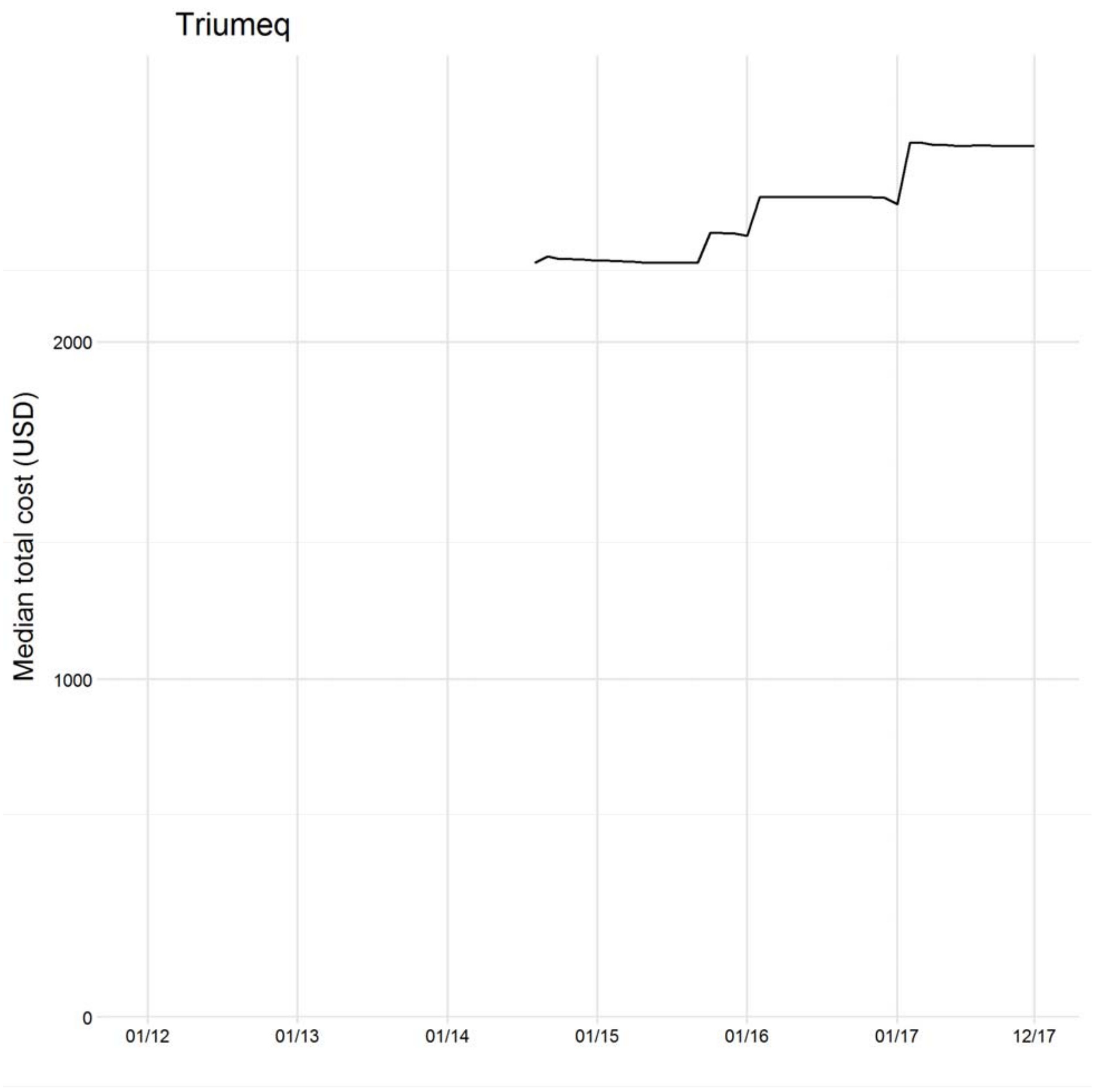

©2019 Wineinger NE et al. *JAMA Network Open*

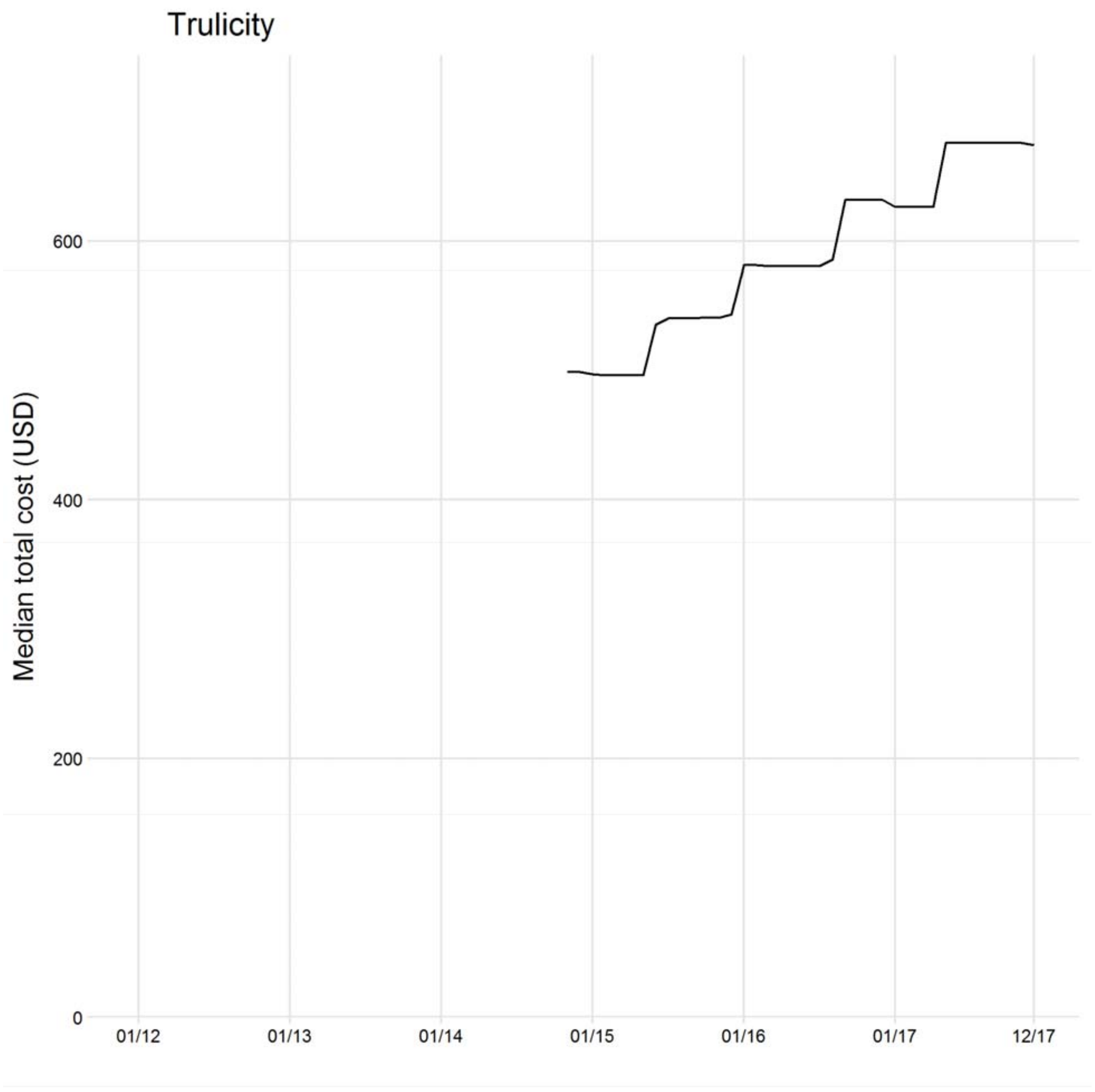

©2019 Wineinger NE et al. *JAMA Network Open*

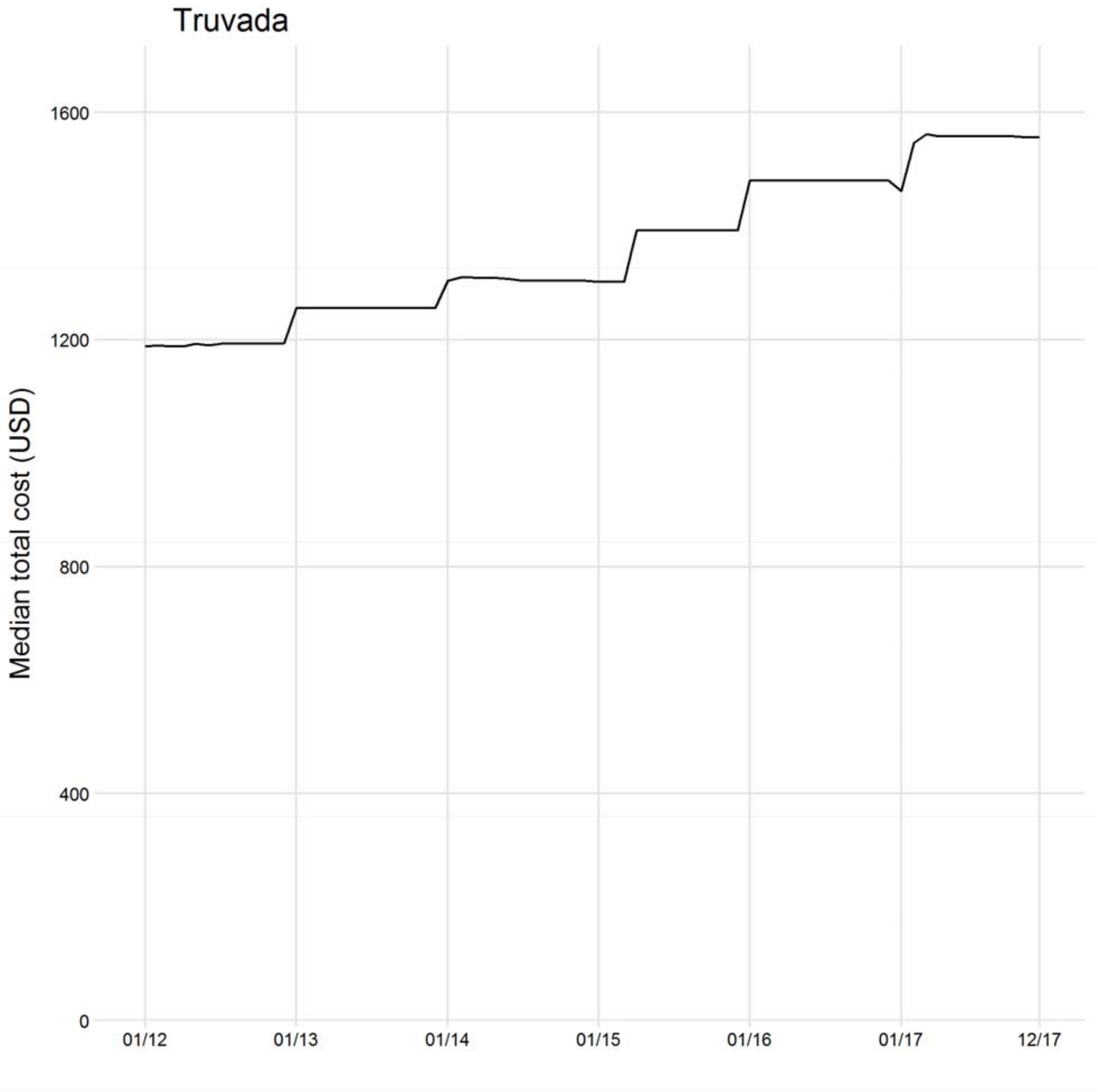

©2019 Wineinger NE et al. *JAMA Network Open*

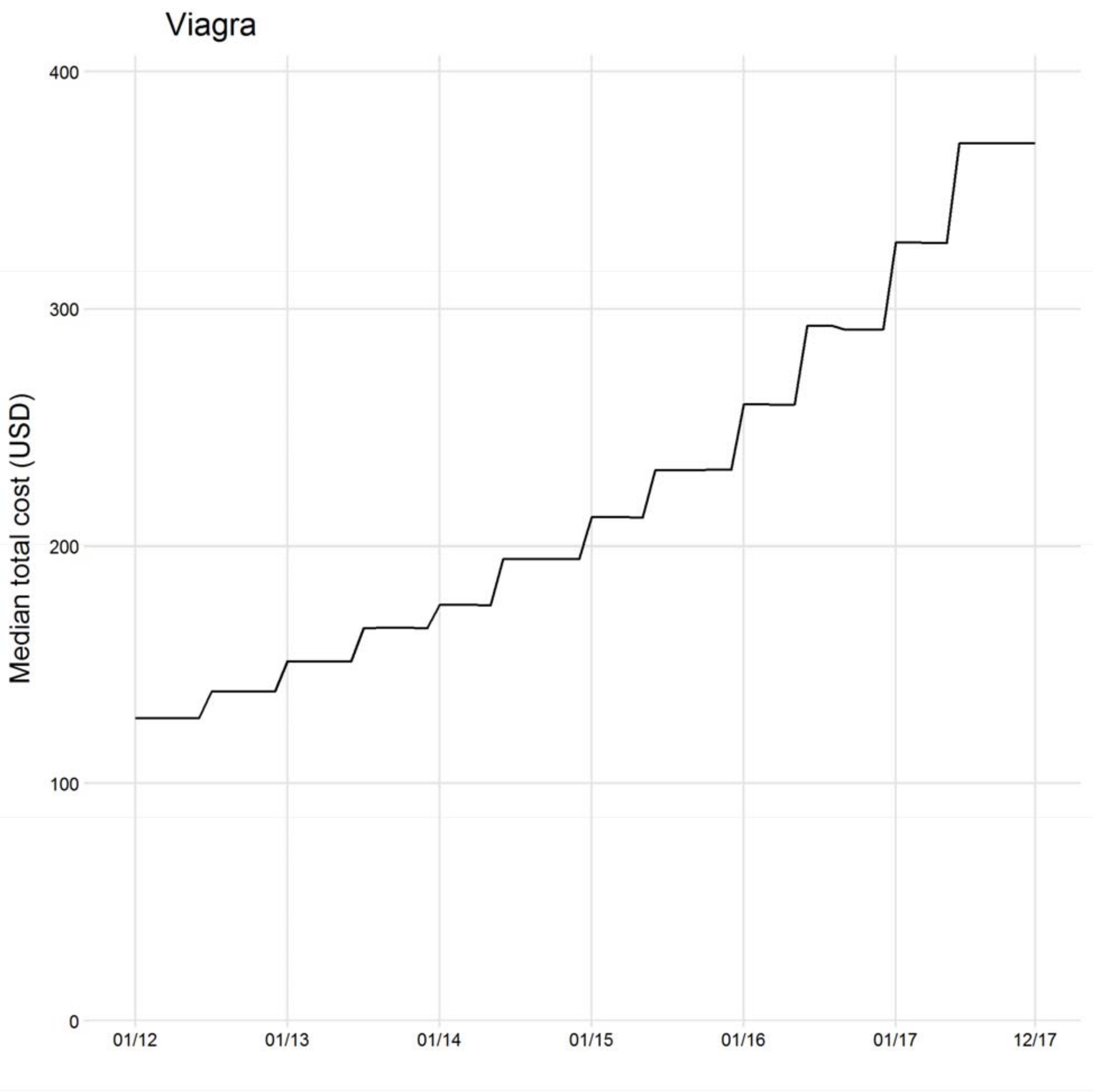

©2019 Wineinger NE et al. *JAMA Network Open*

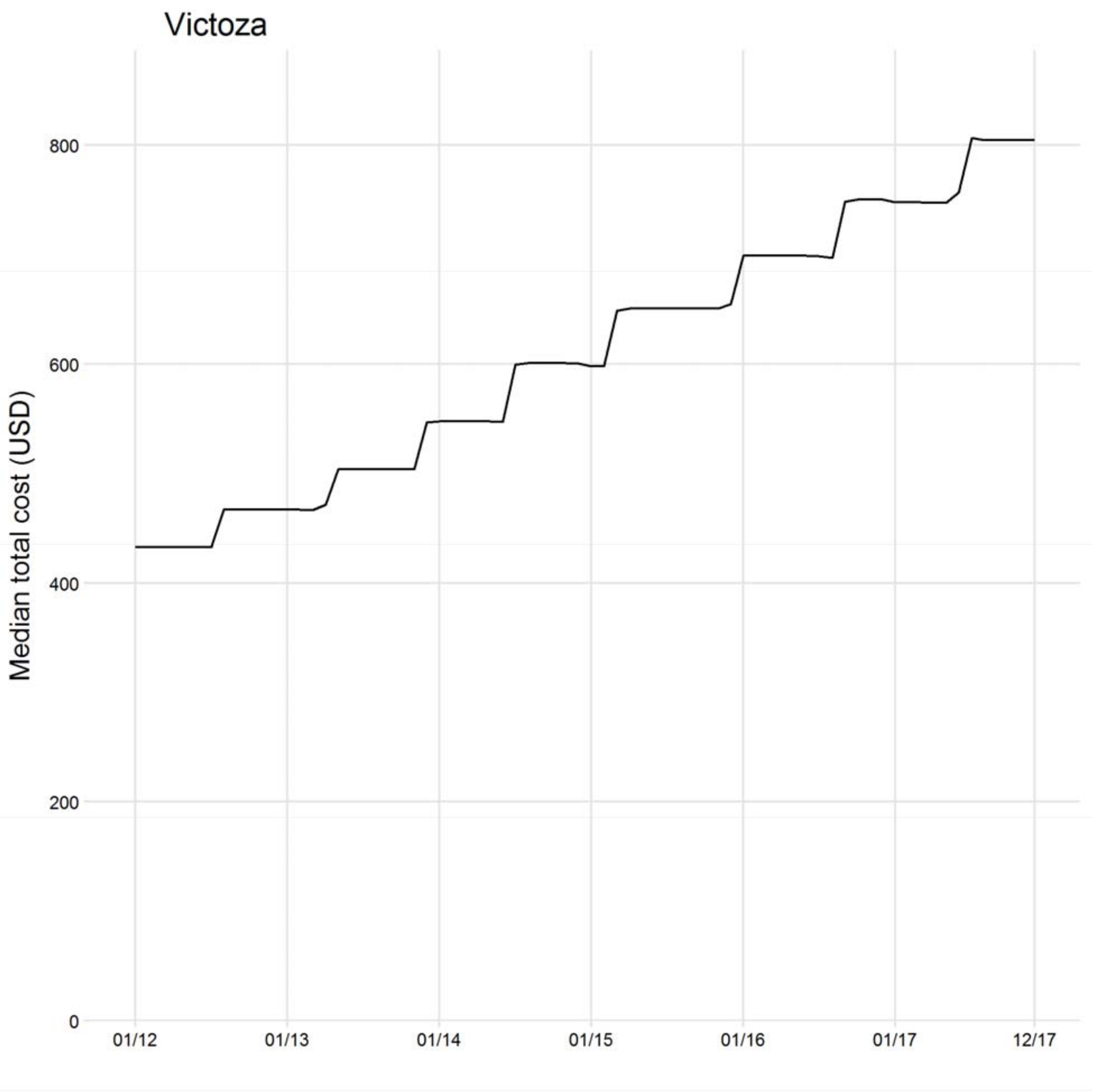

©2019 Wineinger NE et al. *JAMA Network Open*

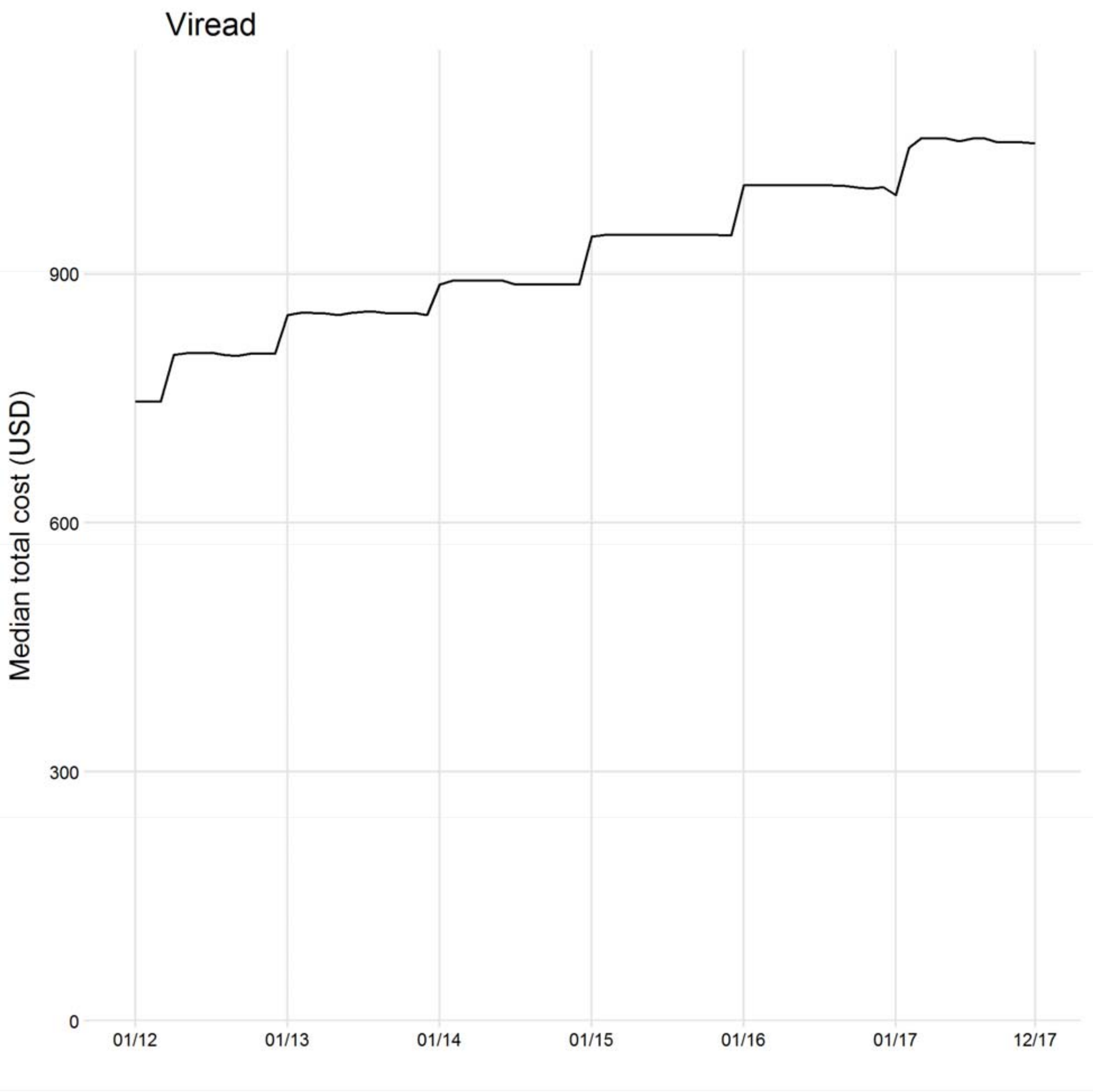

©2019 Wineinger NE et al. *JAMA Network Open*

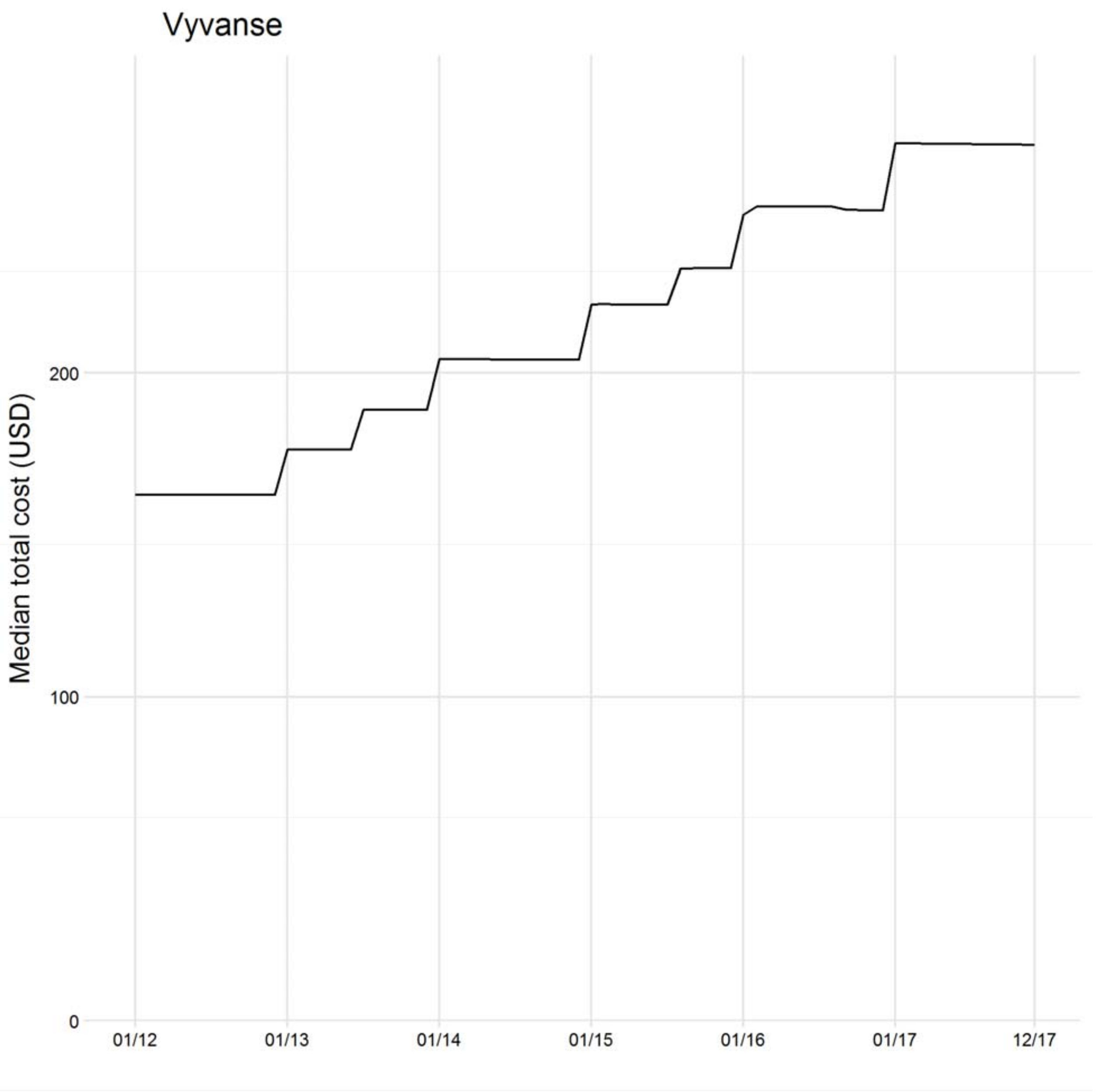

©2019 Wineinger NE et al. *JAMA Network Open*

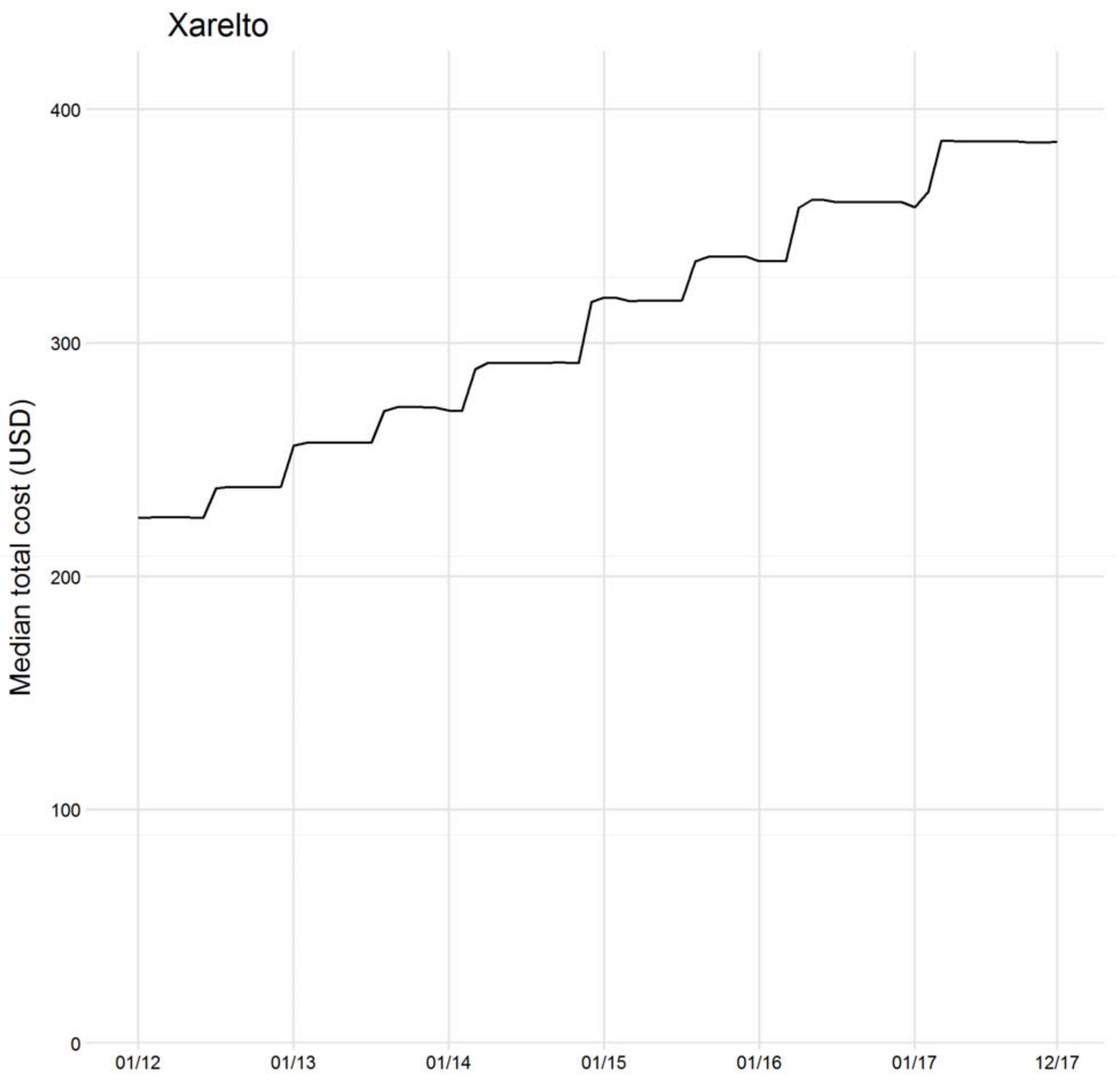

©2019 Wineinger NE et al. *JAMA Network Open*

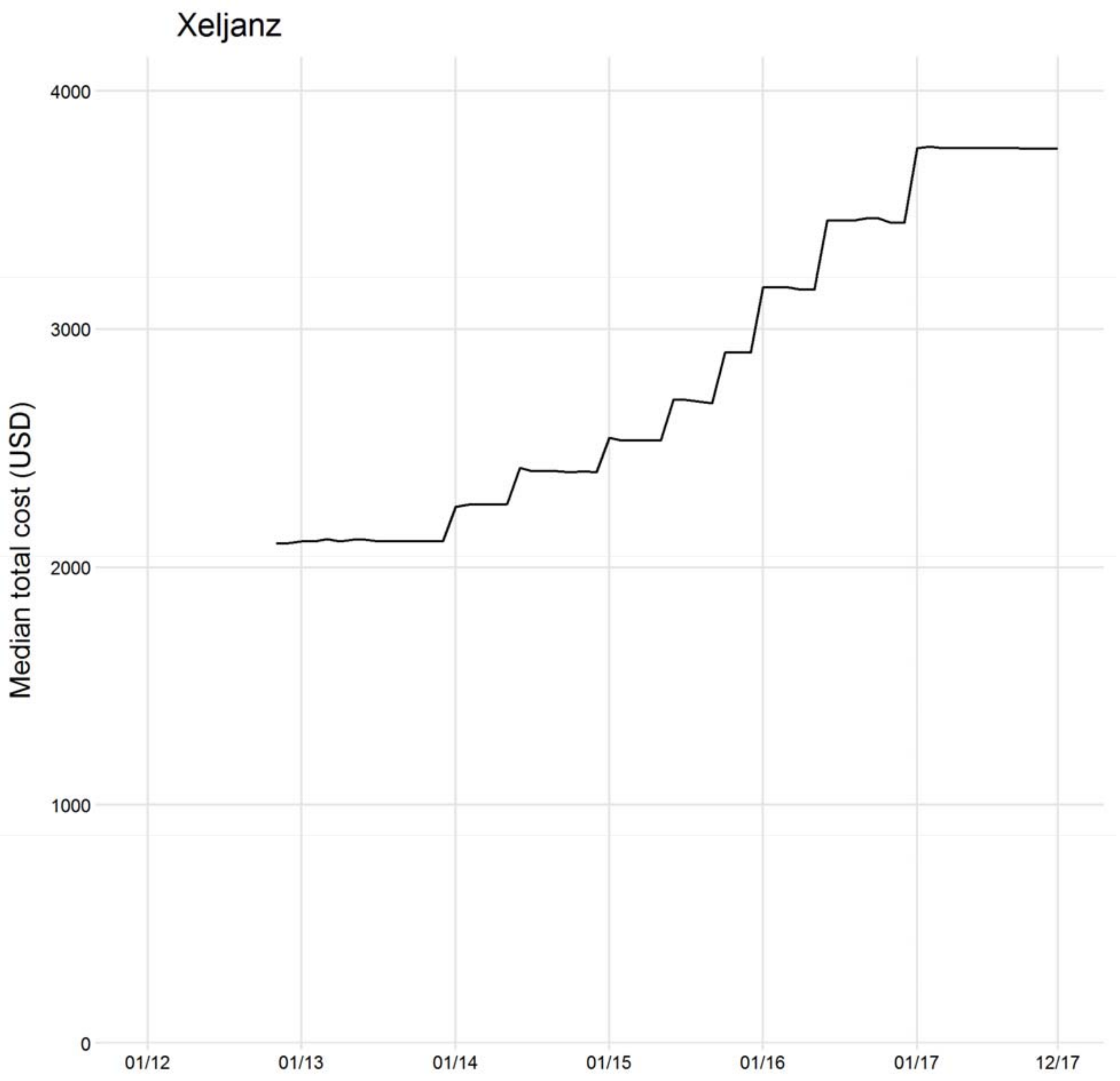

©2019 Wineinger NE et al. *JAMA Network Open*

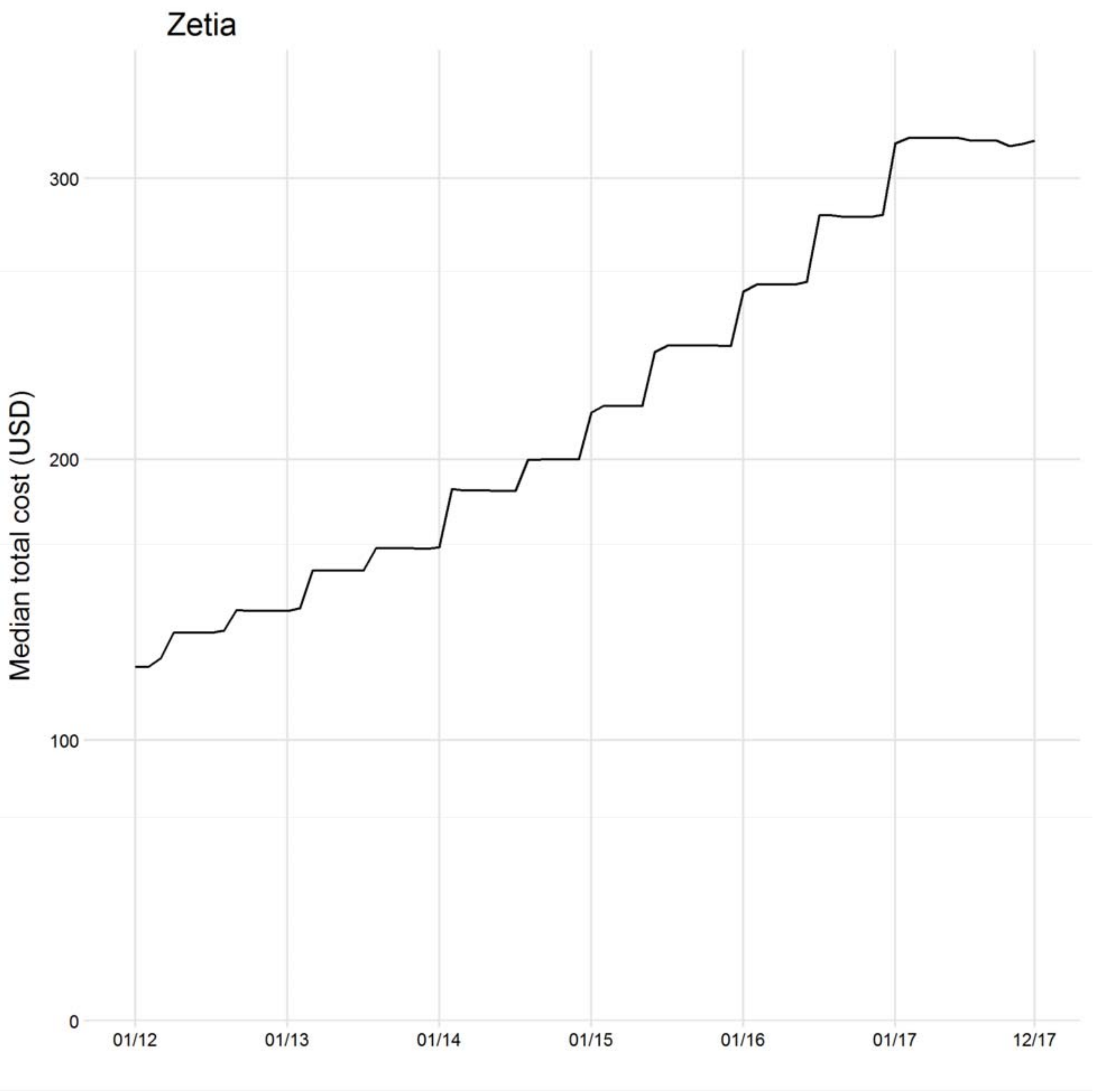

©2019 Wineinger NE et al. *JAMA Network Open*

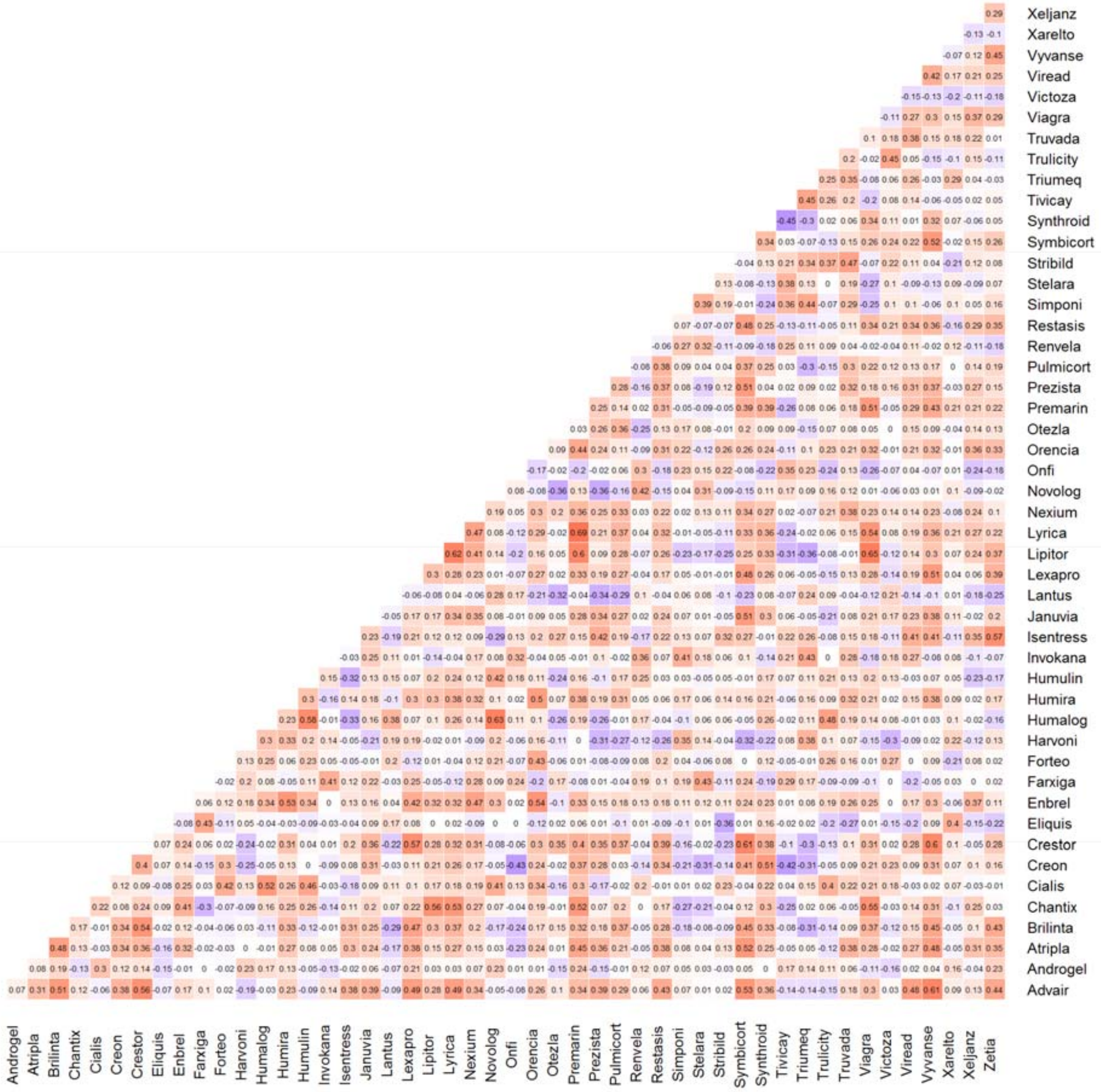

eFigure 2. Spearman correlation between monthly relative cost changes of pairs of drugs.

©2019 Wineinger NE et al. *JAMA Network Open*

eTable 1. Claims = number of claims identified in BCBS data across all NDCs for product, NDC = most common NDC, NDC claims = number of claims with this NDC, Billed quantity = most common billed quantity, Billed QTY claims = number of claims with this NDC and billed quantity.

Product	Claims	NDC	NDC claims	Billed quantity	Billed QTY claims
Abilify Maintena	9917	59148-019-71	5428	1	5274
Actemra	54264	50242-138-01	48124	3.6	25871
Activase	524	50242-041-64	434	1	243
Advair	6785601	0173-0696-00	4122351	60	3490072
Afinitor	48431	0078-0567-51	29351	28	24496
Alimta	291	0002-7623-01	233	2	193
Androgel	2025332	0051-8462-33	1647682	75	1069593
Aranesp	22625	55513-025-04	4436	0.5	1584
Atripla	581651	15584-0101-1	581651	30	503074
Aubagio	92628	58468-0210-2	85275	28	78744
Avastin	1094	50242-060-01	581	4	170
Avonex	97020	59627-333-04	54862	1	49005
Baraclude	63664	0003-1611-12	41915	30	32603
Botox	78613	0023-1145-01	40936	1	17096
Breo_Ellipta	705054	0173-0859-10	466629	60	427821
Brilinta	551709	0186-0777-60	520461	60	444824
Chantix	1638193	0069-0469-56	382327	60	199958
Cialis	3398962	0002-4464-30	1494930	6	401713
Copaxone	569645	68546-325-12	295405	12	269025
Cosentyx	75927	0078-0639-41	56768	2	48632
Creon	351706	0032-1224-01	111721	90	20967
Crestor	11877873	0310-0751-90	4709674	30	3365492
Darzalex	59	57894-502-20	58	80	25
Descovy	57781	61958-2002-1	57781	30	52464
Eliquis	1687440	0003-0894-21	1364578	60	1055326
Enbrel	1614494	58406-445-04	1104479	3.92	871510
Epclusa	14183	61958-2201-1	14183	28	14053
Epogen	5915	55513-144-10	2330	4	917
Erbitux	96	66733-948-23	65	250	28
Exjade	14312	0078-0470-15	9526	60	2663
Eylea	4901	61755-005-02	4901	0.05	3482
Farxiga	821690	0310-6210-30	535916	30	460456
Forteo	224999	0002-8400-01	224999	2.4	189731
Gardasil 9	16217	0006-4119-03	11053	0.5	9739
Genvoya	160083	61958-1901-1	160083	30	148064
Gilenya	109936	0078-0607-15	109936	30	94623
Gleevec	133440	0078-0438-15	66673	30	51772

Harvoni	112915	61958-1801-1	112915	28	111723
Herceptin	740	50242-134-68	689	1	414
H.P. Acthar	8626	63004-8710-1	8626	5	6253
Humalog	3151692	0002-7510-01	1467934	10	399675
Humira	2496282	0074-4339-02	2036856	2	1668610
Humulin	805110	0002-8315-01	241623	10	100226
Ibrance	53628	0069-0189-21	34703	21	34006
Imbruvica	49255	57962-140-09	37820	90	35012
Invega Sustenna	32533	50458-563-01	12870	1	12387
Invega Trinza	1014	50458-608-01	347	1.75	343
Invokamet	244355	50458-543-60	123883	60	98785
Invokana	2233421	50458-141-30	1197030	30	1066751
Isentress	243656	0006-0227-61	242408	60	206483
Jadenu	6281	0078-0656-15	4307	90	1327
Janumet	2856665	0006-0577-61	1358656	60	1095074
Januvia	4631746	0006-0277-31	2618308	30	2197836
Keytruda	152	0006-3026-02	150	8	147
Kyprolis	46	76075-101-01	46	6	23
Lantus	8130408	0088-2219-05	5453873	15	3505554
Levemir	1839372	0169-6438-10	1358136	15	818875
Lexapro	873344	0456-2020-01	425284	30	289524
Lipitor	1228331	0071-0155-23	416898	30	291769
Lovenox	19967	0075-0620-40	6695	12	2213
Lucentis	2997	50242-080-01	1986	0.05	1433
Lupron Depot	67399	0074-3641-03	33406	1	32507
Lyrica	4540293	0071-1014-68	1350597	60	634040
MMR2	23031	0006-4681-00	23031	1	22006
Neulasta	41731	55513-190-01	40570	0.6	30343
Nexium	6931790	0186-5040-31	4846794	30	3783389
Northera	5140	67386-820-19	2049	90	591
Novolog	5518500	0169-6339-10	2582392	15	1589894
NuvaRing	3936757	0052-0273-03	3936757	1	3226520
Odefsey	46459	61958-2101-1	46459	30	41737
Onfi	113030	67386-314-01	72037	60	17167
Opdivo	327	0003-3774-12	231	40	92
Orencia	115926	0003-2188-11	101607	4	93278
Orkambi	17820	51167-809-01	16202	112	15667
Otezla	196944	59572-631-06	191004	60	181626
Perjeta	171	50242-145-01	171	14	138
Plavix	848318	63653-1171-6	376976	30	319888
Pneumovax	251923	0006-4837-03	113249	0.5	104728

Pomalyst	20206	59572-504-21	8369	21	7842
Premarin	4069039	0046-1102-81	1112045	30	823418
Prevnar 13	280309	0005-1971-02	214100	0.5	200724
Prezista	202352	59676-566-30	144184	30	132230
Prolia	61412	55513-710-01	61412	1	61332
ProQuad	101	0006-4171-00	101	1	101
Pulmicort	524198	0186-0916-12	347608	1	294150
Remicade	33743	57894-030-01	33743	4	7182
Renagel	29420	58468-0021-1	27355	90	7638
Renvela	264585	58468-0130-1	256318	90	46482
Restasis	1728075	0023-9163-30	930293	60	496235
Revlimid	187512	59572-410-00	45872	21	32280
Rexulti	83731	59148-038-13	31570	30	28368
Rituxan	3098	50242-053-06	2232	200	1512
Sabril	13493	67386-211-65	7938	120	3112
Sandostatin	6534	0078-0647-81	1743	1	1144
Saxenda	67743	0169-2800-15	67743	15	63386
Sensipar	250842	55513-073-30	164736	30	114103
Simponi	147220	57894-070-02	100573	0.5	80069
Soliris	248	25682-001-01	248	90	91
Spinraza	66	64406-058-01	66	5	59
Sprycel	66870	0003-0852-22	43949	30	38916
Stelara	109836	57894-060-03	57325	0.5	50823
Stribild	222096	61958-1201-1	222096	30	206852
Sustiva	65979	0056-0510-30	63380	30	51695
Sutent	27042	0069-0980-38	10277	28	6889
Symbicort	4005504	0186-0370-20	3082284	10.2	2568869
Synthroid	16535816	0074-6624-90	1918943	30	1295578
Tasigna	42789	0078-0592-87	24036	112	19301
Tecfidera	304874	64406-006-02	284701	60	258274
Tivicay	144046	49702-228-13	143963	30	124048
TNKase	6	50242-120-01	6	1	6
Tresiba	301033	0169-2550-13	203996	9	124789
Trintellix	267094	64764-750-30	123810	30	106978
Triumeq	152961	49702-231-13	152961	30	139948
Trulicity	225488	0002-1433-80	225488	2	197292
Truvada	789062	61958-0701-1	788674	30	695381
Tysabri	11681	64406-008-01	11681	15	11568
Varivax	20556	0006-4827-00	18679	1	17157
Velcade	544	63020-049-01	544	4	276
Viagra	2805494	0069-4220-30	1986138	6	698280

Victoza	2776956	0169-4060-13	1986895	9	1543070
Viread	233571	61958-0401-1	232887	30	188835
Vyvanse	8500693	59417-104-10	1739381	30	1627945
Xarelto	2971580	50458-579-30	1939240	30	1586993
Xeljanz	139255	0069-1001-01	107498	60	98740
Xenazine	11942	67386-421-01	6760	90	2029
Xgeva	4105	55513-730-01	4105	1.7	3592
Xolair	94691	50242-040-62	94691	2	41516
Xtandi	40944	0469-0125-99	40944	120	36676
Yervoy	48	0003-2327-11	28	50	9
Zepatier	2126	0006-3074-02	2126	28	2098
Zetia	3923054	66582-414-31	2616888	30	2156146
Zytiga	53851	57894-150-12	52996	120	44495

©2019 Wineinger NE et al. *JAMA Network Open*

eTable 2: Median monthly costs for all 132 products identified using NDC and billed quantity in eTable 1. Table organized annually; YYYY-MM.

2012

Product	2012-01	2012-02	2012-03	2012-04	2012-05	2012-06	2012-07	2012-08	2012-09	2012-10	2012-11	2012-12
Abilify Maintena												
Actemra												
Activase		109	108	108	121	106	110	111	110	110	111	111
Advair	225	241	241	241	241	241	241	241	240	250	250	250
Afinitor	7620	7629	7620	7627	7627	7581	7622	7581	7627	7622	7602	7918
Alimta	5592	5592	5619	5592	5680	5468		5738	5738	5738	5738	5738
Androgel	321	322	322	322	322	322	322	322	322	321	321	321
Aranesp	592	606	606	610	606	606	608	630	634	634	635	630
Atripla	1776	1784	1784	1782	1784	1784	1784	1804	1807	1807	1807	1807
Aubagio										3521	3532	3510
Avastin	626	606	708	607	708	708	665	629	629	624	629	662
Avonex												
Baraclude	873	873	873	873	874	874	934	934	934	935	935	935
Botox	568	568	568	568	568	568	568	568	568	568	568	568
Breo Ellipta												
Brilinta	236	238	238	238	238	238	238	238	238	237	238	237
Chantix	175	175	175	175	175	175	190	190	190	190	190	190
Cialis	127	139	139	139	139	139	149	151	151	151	151	151
Copaxone												
Cosentyx												
Creon	293	294	294	294	294	320	320	320	320	320	320	320
Crestor	146	146	146	146	146	146	157	157	157	157	157	157
Darzalex												
Descovy												
Eliquis												
Enbrel	1862	1950	1950	1950	1950	1959	1990	2094	2094	2094	2094	2094
Epclusa												
Epogen	532	535	532	536	532	532	532	532	532	537	535	532
Erbitux	2511	2511	2511	2511	2511	2656	2696	2696	2696			
Exjade	4836	4866	4866	4915	4866	4883	4866	4844	4842	4860	4860	5194

©2019 Wineinger NE et al. *JAMA Network Open*

Eylea		1966	1954	1966	1966	1966	1953	1867	1867	1867	1966	1867
Farxiga												
Forteo	1116	1116	1116	1116	1116	1116	1116	1116	1116	1217	1217	1217
Gardasil 9												
Genvoya												
Gilenya												
Gleevec	5891	5928	5928	5923	5922	5915	5928	5928	5928	5928	5928	6446
Harvoni												
Herceptin	3214	3214	3214	3309	3309	3309	3303	3303	3309	3393	3393	3393
H.P. Acthar												
Humalog	126	126	126	126	126	126	132	134	134	134	134	134
Humira	1940	1949	1940	1940	1940	1940	2074	2077	2077	2074	2077	2074
Humulin	67	67	67	67	67	67	68	73	73	73	73	73
Ibrance												
Imbruvica												
Invega Sustenna	1103	1103	1117	1113	1105	1111	1133	1145	1138	1136	1136	1134
Invega Trinza												
Invokamet												
Invokana												
Isentress	1005	1005	1014	1054	1054	1054	1054	1054	1054	1054	1054	1054
Jadenu												
Janumet	220	220	220	219	219	221	230	230	230	230	230	230
Januvia	219	219	219	219	219	220	230	230	230	230	230	230
Keytruda												
Kyprolis												
Lantus	212	211	212	212	218	218	218	218	218	234	235	235
Levemir												
Lexapro	120	129	130	130	130	130	141	141	141	142	142	142
Lipitor	116	116	116	116	116	116	121	121	121	121	121	121
Lovenox	921	917	919	918	924	1008	1008	1011	1011	1011	1009	1011
Lucentis	2053	2053	2053	2055	2057	2053	2053	2053	2053	2053	1995	2053
Lupron Depot	737	742	742	742	736	729	729	729	729	729	729	729
Lyrica	174	174	174	174	174	174	189	189	189	189	189	189
MMR2	54	54	54	54	54	54	54	54	54	56	56	56

©2019 Wineinger NE et al. *JAMA Network Open*

Neulasta	3618	3673	3689	3683	3662	3662	3703	3805	3805	3805	3799	3805
Nexium	188	188	188	188	188	188	194	194	194	194	194	194
Northera												
Novolog	244	244	244	244	244	244	244	263	263	263	263	264
NuvaRing	76	76	76	76	76	76	83	83	83	83	83	83
Odefsey												
Onfi												
Opdivo												
Orencia												
Orkambi												
Otezla												
Perjeta						4148	4148	4148	4148	4148	4148	4326
Plavix	199	199	199	199	199	199	199	199	200	200	200	200
Pneumovax												
Pomalyst												
Premarin	68	68	68	68	68	68	74	74	74	74	74	74
Prevnar 13			133	133	50	62	140	124	52	98	51	133
Prezista											1055	1055
Prolia	851	908	908	908	908	908	908	908	908	908	908	908
ProQuad												
Pulmicort	151	152	152	152	152	151	156	156	156	156	156	156
Remicade	2822	2822	2956	2979	2956	2979	2956	2979	3068	3091	3091	3091
Renagel	264	284	286	285	285	284	285	286	285	284	285	285
Renvela	212	228	228	228	228	228	228	228	228	228	228	228
Restasis	266	266	266	274	279	279	279	279	279	279	279	279
Revlimid	7953	7943	7943	7943	7943	7943	7943	7943	7943	7897	8320	8329
Rexulti												
Rituxan		12347	12347	12683	12683	12683	12683	12683	12683	13029	13029	13176
Sabril	4437	4466	4448	4448	4466	5569	5569	5569	5568	5569	5569	5568
Sandostatin												
Saxenda												
Sensipar	425	426	426	426	426	425	432	436	436	436	436	436
Simponi	1978	2114	2114	2114	2114	2114	2101	2238	2238	2238	2238	2246
Soliris					16629							

©2019 Wineinger NE et al. *JAMA Network Open*

Spinraza												
Sprycel	8049	8283	8334	8335	8334	8334	8334	8334	8334	8334	8334	8411
Stelara	5420	5680	5680	5680	5680	5680	5680	5953	5953	5953	5953	5953
Stribild								2391	2409	2409	2409	2409
Sustiva	589	590	589	589	591	590	591	613	613	614	614	614
Sutent	9732	9732	9741	9709	9682	9741	10045	10151	10088	10151	10229	10151
Symbicort	225	225	225	225	225	225	225	225	225	225	225	225
Synthroid	20	20	20	20	20	20	20	20	20	20	20	23
Tasigna	7753	7753	7753	7731	7731	7720	7698	7698	7698	7698	7698	8042
Tecfidera												
Tivicay												
TNKase									3500			
Tresiba												
Trintellix												
Triumeq												
Trulicity												
Truvada	1188	1190	1188	1188	1193	1190	1193	1193	1193	1193	1193	1193
Tysabri												
Varivax	90	91	90	90	90	90	90	90	90	93	93	93
Velcade	5982	6010	6010	5989	5968	6004			6087	6100	6100	6073
Viagra	127	127	127	127	127	127	139	139	139	139	139	139
Victoza	433	433	433	433	433	433	433	468	467	467	467	467
Viread	746	746	746	802	805	805	805	802	801	804	804	804
Vyvanse	162	162	162	162	162	162	162	162	162	162	162	162
Xarelto	225	225	225	225	225	225	238	238	238	238	238	238
Xeljanz											2098	2100
Xenazine	3986	3980	3980	3986	3986	3984	3961	3945	3954	3961	3945	3946
Xgeva	1691	1711	1713	1713	1713	1722	1713	1713	1722	1713	1753	1753
Xolair	1361	1361	1361	1416	1416	1416	1416	1416	1416	1466	1443	1449
Xtandi									7582	7600	7599	7600
Yervoy	30312	30312	30120	30120								
Zepatier												
Zetia	126	126	129	138	138	138	138	139	146	146	146	146
Zytiga	5604	5598	5598	5598	5593	5867	5929	5929	5867	5867	5867	5867

©2019 Wineinger NE et al. *JAMA Network Open*

2013

Product	2013-01	2013-02	2013-03	2013-04	2013-05	2013-06	2013-07	2013-08	2013-09	2013-10	2013-11	2013-12
Abilify Maintena				1467	1480	1495	1498	1496	1491	1496	1477	1493
Actemra											2653	2653
Activase	111	112	110	110	110	110	123	113	110	111	120	111
Advair	250	267	267	267	267	268	275	276	275	275	276	275
Afinitor	7948	7948	7951	7951	7918	7984	8385	8420	8385	8389	8385	8385
Alimta	5891						5950	5950	5950	5950	5950	5950
Androgel	352	353	353	353	353	353	353	353	353	387	388	388
Aranesp	630	630	651	645	651	651	651	651	651	651	654	653
Atripla	1926	1926	1926	1926	1926	1926	1926	1926	1926	1926	1926	1926
Aubagio	3531	3549	3548	3548	3532	3635	3880	3880	3899	3926	4290	4290
Avastin	671	671	671	640	671	633	643	646	643	644	642	645
Avonex												
Baraclude	1018	1018	1018	1018	1018	1018	1059	1059	1059	1059	1059	1059
Botox	565	565	565	565	565	565	565	565	565	565	565	565
Breo Ellipta											275	273
Brilinta	244	244	244	244	244	244	244	244	244	244	244	244
Chantix	208	208	208	208	208	208	218	218	218	218	218	218
Cialis	165	165	165	165	165	165	180	180	180	180	180	181
Copaxone												
Cosentyx												
Creon	350	351	351	351	351	351	351	351	351	351	351	351
Crestor	166	166	166	166	166	166	176	176	176	176	176	176
Darzalex												
Descovy												
Eliquis	258	257	257	257	257	257	257	257	272	272	272	272
Enbrel	2222	2229	2229	2229	2229	2238	2382	2382	2382	2382	2378	2378
Epclusa												
Epogen	532	532	532	532	535	552	562	558	557	557	558	558
Erbitux		26429										
Exjade	5194	5218	5194	5194	5218	5194	5194	5190	5194	5194	5194	5194
Eylea	1955	1887	1883	1927	1927	1867	1867	1867	1867	1867	1867	1867

©2019 Wineinger NE et al. *JAMA Network Open*

Farxiga												
Forteo	1217	1217	1217	1217	1255	1326	1326	1326	1326	1326	1326	1344
Gardasil 9												
Genvoya												
Gilenya												
Gleevec	6475	6475	6475	6475	6475	6475	6475	7026	7027	7027	7026	7027
Harvoni												
Herceptin	3218	3337	3318	3327	3327	3327	3483	3438	3438	3438	3438	3438
H.P. Acthar	29809	30214	30214	30185	30214	30455	30455	30455	30428	30361	30361	30361
Humalog	144	144	144	144	144	144	144	157	157	157	157	165
Humira	2194	2214	2214	2214	2214	2214	2351	2360	2353	2353	2353	2370
Humulin	78	78	78	78	78	83	85	85	85	85	84	86
Ibrance												
Imbruvica											8326	8363
Invega Sustenna	1165	1168	1169	1166	1168	1166	1200	1203	1200	1200	1214	1206
Invega Trinza												
Invokamet												
Invokana				271	271	271	271	271	271	270	270	270
Isentress	1051	1055	1102	1102	1102	1102	1102	1102	1102	1102	1102	1102
Jadenu												
Janumet	252	253	253	253	253	253	253	265	265	265	265	265
Januvia	251	253	253	253	253	253	253	265	265	265	265	265
Keytruda												
Kyprolis										10100	10100	10112
Lantus	235	235	235	235	258	258	258	282	283	283	283	298
Levemir												
Lexapro	154	154	154	154	154	154	168	168	168	168	168	168
Lipitor	132	132	132	132	132	132	144	144	144	144	144	144
Lovenox	1011	1010	1011	1014	993	1010	1008	1010	1009	1010	1018	1012
Lucentis	2041	2041	2041	2041	2041	2041	2057	2041	2041	2049	2049	2057
Lupron Depot	799	809	804	804	804	807	804	809	799	809	809	809
Lyrica	207	207	207	207	207	207	226	226	226	226	226	226
MMR2	56	56	56	56	56	59	59	59	58	58	58	58
Neulasta	3853	3939	3939	3949	3939	3949	4005	4147	4180	4161	4132	4132

©2019 Wineinger NE et al. *JAMA Network Open*

Nexium	205	205	205	205	205	205	230	230	230	230	230	230
Northera												
Novolog	281	281	281	281	281	281	282	304	303	303	304	333
NuvaRing	86	87	87	87	87	87	91	91	91	91	91	91
Odefsey												
Onfi												457
Opdivo												
Orencia											2430	2423
Orkambi												
Otezla												
Perjeta						4169						
Plavix	199	199	199	199	198	198	198	198	198	198	198	198
Pneumovax												
Pomalyst		10781	10618	10466	10466	10529	10618	10545	10529	10499	10618	10618
Premarin	81	81	81	81	81	81	88	88	88	88	88	88
Prevnar 13	131	131	98	141	141	66	126	53	53	98	98	112
Prezista	1119	1119	1119	1119	1119	1119	1119	1119	1119	1119	1119	1119
Prolia	903	903	903	903	903	903	903	918	872	918	918	918
ProQuad												
Pulmicort	165	165	165	165	165	166	166	166	165	165	165	165
Remicade	3049	3049	3194	3194	3194	3194	3194	3194	3311	3315	3315	3315
Renagel	285	288	308	308	308	333	336	335	335	339	369	369
Renvela	228	230	246	246	246	266	268	268	268	270	294	294
Restasis	290	292	292	292	292	307	307	307	307	307	307	307
Revlimid	8329	8329	8253	8493	8493	8493	8493	8493	8493	8747	8747	8774
Rexulti												
Rituxan	12951	12951	12951	12951	12951	12951	13304	13304	13304	13304	13304	13304
Sabril	6780	6899	6923	6899	6923	6923	6923	6932	6932	6933	6933	6933
Sandostatin												
Saxenda												
Sensipar	448	452	452	452	452	452	455	475	475	475	475	475
Simponi	2245	2392	2401	2401	2401	2401	2400	2557	2567	2566	2566	2566
Soliris	17357	17357	17357	17357	17357	17357	17357	17790	17790	17790	17790	17790
Spinraza												

©2019 Wineinger NE et al. *JAMA Network Open*

Sprycel	8687	8689	8689	8689	8689	8685	8689	8689	8797	8950	8911	8912
Stelara	5917	6317	6317	6317	6317	6317	6317	6745	6745	6745	6745	6745
Stribild	2402	2402	2402	2402	2402	2402	2402	2402	2402	2402	2402	2402
Sustiva	673	673	673	673	673	673	673	673	673	673	673	673
Sutent	10989	10963	10955	10849	10945	11008	11486	11501	11449	11517	11391	11511
Symbicort	242	242	242	242	242	242	242	242	242	242	242	242
Synthroid	24	24	23	23	23	23	25	25	25	25	25	25
Tasigna	8077	8075	8075	8098	8075	8075	8075	8075	8075	8309	8358	8358
Tecfidera				4577	4627	4616	4627	4627	4627	4627	4632	4632
Tivicay								1190	1206	1205	1206	1206
TNKase												
Tresiba												
Trintellix												
Triumeq												
Trulicity												
Truvada	1255	1255	1255	1255	1255	1255	1255	1255	1255	1255	1255	1255
Tysabri									4441	4305	4305	4305
Varivax	93	93	93	94	94	97	97	97	97	97	97	97
Velcade			6210			6321	6288	6234	6307	6214	6400	
Viagra	152	151	151	151	151	151	165	166	166	166	166	165
Victoza	467	467	466	471	504	504	504	504	504	504	504	547
Viread	850	853	853	852	851	853	854	854	852	852	853	851
Vyvanse	176	176	176	176	176	176	189	189	189	189	189	189
Xarelto	256	257	257	257	257	257	257	271	272	272	272	272
Xeljanz	2108	2108	2116	2108	2114	2114	2108	2107	2110	2108	2109	2108
Xenazine	4474	4490	4474	4482	4482	4474	4482	4482	4482	4482	4474	4474
Xgeva	1653	1659	1653	1665	1669	1665	1665	1697	1697	1696	1696	1697
Xolair	1443	1443	1443	1501	1501	1501	1501	1501	1501	1561	1561	1561
Xtandi	7599	7577	7599	7598	7599	7573	7573	7573	8010	8020	8020	8020
Yervoy							30842	30842	30842			
Zepatier												
Zetia	146	147	161	161	161	161	161	168	168	168	168	168
Zytiga	5867	5867	6408	6447	6447	6447	6447	6447	6447	6599	6892	6892

©2019 Wineinger NE et al. *JAMA Network Open*

2014

Product	2014-01	2014-02	2014-03	2014-04	2014-05	2014-06	2014-07	2014-08	2014-09	2014-10	2014-11	2014-12
Abilify Maintena	1525	1519	1519	1517	1517	1519	1563	1566	1563	1563	1563	1563
Actemra	2654	2654	2656	2654	2654	2654	2813	2813	2812	2791	2791	2783
Activase	114	115	118	120	115	115	120	119	121	119	119	122
Advair	289	291	291	290	290	290	290	290	290	290	290	290
Afinitor	9214	9214	9214	9214	9214	9235	9665	9665	9665	9607	9607	9665
Alimta	5908	5793	5850	5793	5793	5793	5849	5849	5849	5849	5849	5964
Androgel	386	386	386	386	386	386	412	412	412	412	412	412
Aranesp	652	650	645	646	643	673	681	679	682	675	678	685
Atripla	2033	2043	2041	2038	2038	2082	2084	2084	2084	2084	2084	2084
Aubagio	4286	4441	4670	4670	4670	4670	4634	4725	4895	4890	4890	4933
Avastin	649	663	666	662	656	666	677	678	674	720	671	
Avonex												
Baraclude	1183	1188	1189	1188	1188	1188	1259	1260	1259	1260	1261	1260
Botox	578	578	578	578	578	578	578	578	578	578	578	578
Breo Ellipta	274	273	274	273	273	273	273	273	273	273	273	273
Brilinta	250	250	250	250	250	250	252	258	258	258	258	257
Chantix	231	231	231	231	231	252	253	252	253	253	253	253
Cialis	195	195	195	195	195	195	212	213	213	213	213	233
Copaxone		4651	4623	4623	4623	4623	4623	4623	4623	4595	4595	4595
Cosentyx												
Creon	384	384	384	383	384	383	420	421	421	421	421	421
Crestor	186	186	186	186	186	186	191	191	191	191	191	191
Darzalex												
Descovy												
Eliquis	271	271	276	297	297	297	297	297	297	297	297	312
Enbrel	2539	2547	2547	2547	2547	2690	2722	2722	2722	2690	2788	2903
Epclusa												
Epogen	565	557	565	562	565	594	593	593	593	593	598	593
Erbitux												
Exjade	5692	5708	5692	5692	5682	5674	6218	6236	6218	6244	6236	6218
Eylea	1944	1944	1916	1944	1944	1944	1940	1944	1916	1944	1887	1887

Farxiga												320
Forteo	1446	1447	1451	1447	1448	1446	1576	1577	1576	1576	1576	1789
Gardasil 9												
Genvoya												
Gilenya												
Gleevec	7694	7722	7722	7722	7722	7722	8373	8448	8448	8347	8347	8347
Harvoni										31980	31942	31942
Herceptin	3523	3562	3562	3562	3562	3505	3657	3600	3600	3600	3600	3600
H.P. Acthar	30455	31879	31879	31879	31642	31879	31879	31879	31879	31826	31826	31879
Humalog	172	172	172	171	171	187	188	188	188	188	189	207
Humira	2515	2515	2515	2515	2509	2508	2706	2706	2706	2706	2801	2919
Humulin	92	92	92	92	92	95	100	100	100	100	100	110
Ibrance												
Imbruvica	8326	8326	8326	8326	8326	8326	8326	8326	8844	8908	8908	8908
Invega Sustenna	1237	1245	1240	1240	1240	1240	1272	1274	1273	1277	1272	1272
Invega Trinza												
Invokamet								321	319	319	319	319
Invokana	295	296	296	296	296	296	296	318	319	319	319	319
Isentress	1148	1150	1150	1150	1149	1147	1145	1145	1145	1145	1145	1145
Jadenu												
Janumet	291	291	290	290	290	290	290	307	307	307	307	307
Januvia	290	290	290	290	289	289	289	306	306	306	306	306
Keytruda												
Kyprolis	10447	10257							10509	10509		
Lantus	310	310	310	310	310	340	340	340	340	340	375	380
Levemir		24				339	339	339	340	339	343	379
Lexapro	181	182	182	182	181	181	197	197	198	198	198	198
Lipitor	156	156	156	156	156	170	170	170	170	170	170	170
Lovenox	995	1005	1005	995	995	1005	996	996	995	988	976	999
Lucentis	2028	2028	2028	2028	2028	2028	2028	2028	2044	2028	2028	2028
Lupron Depot	865	872	873	873	873	873	869	868	873	873	873	873
Lyrica	246	246	246	246	246	269	269	269	269	269	269	269
MMR2	58	58	58	72	73	73	73	73	73	73	77	77
Neulasta	4156	4241	4241	4241	4280	4292	4344	4502	4502	4448	4448	4430

©2019 Wineinger NE et al. *JAMA Network Open*

Nexium	242	242	242	242	242	242	242	242	242	242	241	241
Northera										1429	1429	1438
Novolog	332	332	332	331	332	364	364	364	364	364	367	400
NuvaRing	95	95	95	95	95	95	95	104	104	104	104	104
Odefsey												
Onfi	496	496	495	496	496	496	496	496	496	496	514	570
Opdivo												
Orencia	2482	2558	2558	2558	2557	2555	2729	2731	2731	2707	2707	2921
Orkambi												
Otezla				1932	1931	1929	1922	1922	1920	1917	1914	1913
Perjeta	4078	4145	4145	4145		4181	4145	4116	4145	4145	4145	4274
Plavix	197	197	197	197	197	197	197	197	197	197	197	197
Pneumovax							86	76	76	78	83	83
Pomalyst	10770	10820	10820	10845	10785	10820	10846	11170	11145	11170	11170	11168
Premarin	93	93	93	93	93	102	102	102	102	102	102	102
Prevnar 13	98	89	98	84	98	54	54	145	160	160	160	160
Prezista	1182	1190	1190	1188	1187	1185	1183	1183	1184	1184	1183	1183
Prolia	913	889	928	928	928	928	928	952	952	952	952	952
ProQuad												
Pulmicort	174	175	174	174	174	174	174	174	174	174	174	174
Remicade	3318	3311	3452	3473	3452	3473	3473	3473	3616	3616	3639	3616
Renagel	367	377	403	402	403	441	442	443	443	443	486	487
Renvela	294	312	323	322	322	354	354	354	354	354	388	389
Restasis	320	326	326	326	326	326	332	336	336	336	336	336
Revlimid	8747	8668	9015	9015	9015	9015	9015	9015	9415	9415	9415	9505
Rexulti												
Rituxan	13584	13584	13584	13584	13584	13584	13955	13955	13955	13955	13955	13955
Sabril	8572	8623	8576	8581	8574	8572	8573	8573	8573	8527	8522	8572
Sandostatin												
Saxenda												
Sensipar	513	513	515	514	514	528	530	529	530	529	553	561
Simponi	2566	2733	2743	2741	2743	2739	2739	2994	3012	2994	2994	2994
Soliris	16959	16959	16959	16959	16959	16959	16959	17430	17430	17430	17430	18148
Spinraza												

©2019 Wineinger NE et al. *JAMA Network Open*

Sprycel	8911	8910	9070	9258	9258	9258	9258	9258	9258	9596	9677	9677
Stelara	6704	7158	7158	7158	7158	7158	7158	7158	7158	7158	7433	7433
Stribild	2505	2508	2507	2507	2505	2500	2500	2500	2500	2499	2500	2499
Sustiva	730	734	734	734	734	781	781	781	781	781	781	781
Sutent	12086	12086	12012	12012	12068	12080	12080	12106	12079	12007	12079	12079
Symbicort	261	261	261	261	260	260	260	261	261	261	261	260
Synthroid	27	27	27	27	27	27	30	30	30	30	30	30
Tasigna	8358	8357	8714	8766	8766	8766	8766	8787	8766	8867	9196	9196
Tecfidera	5072	5067	5044	5012	4997	4997	4989	4989	4989	5116	5238	5238
Tivicay	1200	1259	1259	1258	1258	1257	1254	1254	1253	1253	1254	1253
TNKase			3815									
Tresiba												
Trintellix												
Triumeq								2232	2252	2244	2244	2242
Trulicity											499	499
Truvada	1304	1310	1310	1309	1309	1306	1304	1304	1304	1304	1304	1304
Tysabri	4528	4528	4528	4528	4528	4528	4528	4528	4528	4528	4528	4528
Varivax	96	97	97	98	98	97	98	98	98	98	113	113
Velcade	6252	6272	6272	6272		6291	6338	6338	6358	6377	6358	6377
Viagra	175	175	175	175	175	195	195	195	195	195	195	195
Victoza	548	548	548	548	548	547	600	601	601	601	601	601
Viread	887	892	892	892	892	892	887	887	887	887	887	887
Vyvanse	204	204	204	204	204	204	204	204	204	204	204	204
Xarelto	271	271	289	292	291	292	291	291	292	292	291	318
Xeljanz	2252	2262	2263	2262	2263	2417	2401	2401	2401	2397	2401	2397
Xenazine	5097	5110	5101	5101	5101	5101	5101	5094	5094	5094	5094	5094
Xgeva	1717	1737	1754	1737	1754	1737	1754	1788	1788	1767	1771	1771
Xolair	1561	1561	1561	1623	1623	1625	1649	1649	1649	1714	1714	1714
Xtandi	8010	8000	7953	7972	7935	7953	8392	8409	8422	8418	8422	8422
Yervoy								32990	32990			
Zepatier												
Zetia	169	189	189	189	189	189	189	200	200	200	200	200
Zytiga	6891	6850	6805	6809	6801	6805	6801	7179	7285	7285	7259	7285

©2019 Wineinger NE et al. *JAMA Network Open*

2015

Product	2015-01	2015-02	2015-03	2015-04	2015-05	2015-06	2015-07	2015-08	2015-09	2015-10	2015-11	2015-12
Abilify Maintena	1602	1612	1606	1602	1602	1602	1665	1665	1675	1665	1674	1665
Actemra	2852	2859	2859	2852	2851	2851	3048	3088	3085	3080	3085	3085
Activase	126	131	130	127	123	132	135	127	131	146	160	131
Advair	300	303	303	303	303	303	315	315	315	315	315	315
Afinitor	10559	10603	10622	10603	10622	10622	10559	10527	10521	10521	10521	10521
Alimta	6106	6106	6106	6106	6106	6106	6166	6166	6166	6166	6166	6166
Androgel	439	439	439	439	482	482	482	482	482	482	482	482
Aranesp	675	677	710	714	714	714	715	709	712	712	708	708
Atripla	2155	2157	2156	2245	2247	2247	2247	2247	2245	2247	2247	2246
Aubagio	5119	5119	5114	5114	5114	5119	5089	5088	5322	5394	5394	5394
Avastin	650	684	718	0	707	700	697	733	733	733	733	733
Avonex				5014	5014	5014	5014	5026	5315	5315	5315	5315
Baraclude	1260	1260	1260	1256	1256	1256	1259	1259	1259	1259	1259	1259
Botox	579	579	579	579	579	579	579	579	579	579	579	579
Breo Ellipta	284	286	286	286	286	286	286	286	286	286	286	286
Brilinta	276	277	277	277	277	277	290	290	290	290	290	290
Chantix	276	276	276	276	276	293	293	293	293	293	293	293
Cialis	232	232	232	232	236	255	255	255	255	255	255	281
Copaxone	4958	4958	4958	4958	4958	4958	4958	4928	4928	4928	4910	4910
Cosentyx			3479	3458	3447	3440	3641	3664	3673	3678	3685	3664
Creon	448	448	447	449	448	448	448	448	448	448	448	450
Crestor	209	210	210	210	210	210	220	220	220	220	220	220
Darzalex												
Descovy												
Eliquis	319	319	319	319	319	319	319	322	338	338	338	338
Enbrel	2903	2903	2903	2903	3171	3190	3179	3171	3400	3410	3409	3421
Epclusa												
Epogen	594	601	625	630	624	623	623	618	622	618	622	622
Erbitux									2677			
Exjade	6813	6833	6862	6833	6833	6833	6833	6833	6841	6817	6853	6833
Eylea	1877	1877	1877	1877	1877	1877	1877	1877	1877	1877	1877	1877

Farxiga	318	325	349	349	349	349	349	349	349	368	369	369
Forteo	1788	1788	1788	1788	1788	2042	2042	2042	2042	2042	2042	2333
Gardasil 9			175	167	165	165	161	165	165	165	176	176
Genvoya											2615	2616
Gilenya		5543	5453	5692	5790	5790	5790	5745	5745	6078	6080	6078
Gleevec	9118	9174	9365	9363	9354	9368	9544	10280	10296	10204	10204	10235
Harvoni	31752	31752	31564	31563	31563	31563	31563	31563	31563	31563	31450	31399
Herceptin	3774	3729	3752	3761	3774	3774	3862	3885	3885	3885	3885	3885
H.P. Acthar	32518	32518	32464	32491	32491	33796	33898	34306	34249	34306	34306	34306
Humalog	204	204	204	204	204	224	224	224	224	224	224	238
Humira	2919	2902	2902	3189	3189	3189	3189	3189	3441	3441	3433	3433
Humulin	105	110	110	110	110	121	121	121	121	121	121	129
Ibrance		10001	10012	10019	10019	10018	10018	9989	9989	10017	9989	9989
Imbruvica	8871	8871	9242	9270	9270	9270	9270	9270	9270	9687	9687	9687
Invega Sustenna	1304	1305	1305	1304	1309	1355	1355	1355	1355	1360	1360	1360
Invega Trinza							3858	3925	3858		4095	3870
Invokamet	318	349	349	349	349	349	349	349	369	369	369	369
Invokana	318	348	349	349	349	349	349	349	368	369	369	369
Isentress	1218	1221	1221	1222	1222	1222	1221	1221	1221	1222	1222	1221
Jadenu				10484	10311	10311	10293	10280	10323	10311	10288	10333
Janumet	334	336	336	336	336	336	336	336	336	336	339	369
Januvia	332	336	336	336	336	336	336	336	336	335	338	368
Keytruda											8728	8728
Kyprolis		10422		10672						11249	11249	11249
Lantus	379	379	379	379	379	379	379	379	379	379	379	379
Levemir	379	378	378	378	378	378	378	380	409	409	410	409
Lexapro	214	214	214	214	215	214	238	238	238	238	238	238
Lipitor	184	184	184	184	184	193	193	193	193	193	193	193
Lovenox	981	989	979	979	992	970	989	989	992	979	928	977
Lucentis	2040	2040	2040	2040	2040	2040	2040	2040	2040	2040	2040	2040
Lupron Depot	929	928	929	992	995	993	998	987	984	988	987	987
Lyrica	293	293	293	293	293	320	320	320	320	320	320	320
MMR2	78	78	78	78	78	78	78	77	78	77	80	80
Neulasta	4616	4658	4666	4666	4674	4666	4759	4886	4895	4895	4895	4895

©2019 Wineinger NE et al. *JAMA Network Open*

Nexium	241	241	241	241	242	255	255	255	255	256	257	257
Northera	1430	1442	1430	1570	1571	1572	1571	1573	1573	1727	1739	1739
Novolog	399	399	399	399	403	439	439	439	439	439	439	465
NuvaRing	108	108	108	108	108	113	114	114	114	114	114	114
Odefsey												
Onfi	568	568	646	646	646	646	646	646	766	775	775	775
Opdivo						9356	9356	9356	9356	9356	9495	9480
Orencia	2921	2917	2915	2915	2915	3200	3204	3200	3200	3200	3190	3430
Orkambi							20179	20143	20130	20082	20082	20082
Otezla	1913	1913	2047	2063	2063	2063	2260	2259	2259	2259	2259	2259
Perjeta	4130	4156	4156	4156	4130	4130	4156	4251	4251	4251	4251	4251
Plavix	197	196	197	197	197	197	197	196	196	196	197	196
Pneumovax	83	83	83	82	82	82	83	82	82	84	93	93
Pomalyst	11232	11232	11229	11368	11364	11398	11710	11697	11639	12019	12068	12019
Premarin	111	111	111	111	111	122	122	122	122	122	122	122
Prevnar 13	160	160	160	160	160	160	160	171	171	171	179	179
Prezista	1272	1273	1273	1277	1277	1277	1273	1273	1273	1275	1272	1273
Prolia	942	983	983	983	983	983	983	1009	1009	1009	1009	1009
ProQuad								168	168	168	182	182
Pulmicort	185	185	186	186	187	187	189	188	189	188	189	188
Remicade	3641	3641	3815	3815	3815	3815	3798	3798	3982	3985	3985	3985
Renagel	487	487	535	535	535	535	535	535	588	588	588	588
Renvela	388	388	427	427	427	427	427	427	469	470	470	470
Restasis	361	361	361	361	362	395	395	395	395	396	395	395
Revlimid	9697	9697	9697	9697	9697	9742	9988	9988	9988	10388	10388	10388
Rexulti								881	880	881	881	879
Rituxan	14450	14450	14450	14450	14450	14450	14916	14916	14916	14916	14916	14916
Sabril	10534	10607	10652	10659	10652	10652	10652	10659	10716	10717	10716	10717
Sandostatin					3108	3095	3123	3133	3126	3143	3157	3139
Saxenda				1084	1084	1084	1084	1084	1084	1084	1084	1084
Sensipar	560	560	560	560	604	605	605	605	605	652	653	653
Simponi	2980	2980	3261	3271	3271	3271	3271	3261	3473	3496	3490	3486
Soliris	18394	18866	19224	19224	19224	19224	19224	18250	18239	18239	18239	18239
Spinraza												

©2019 Wineinger NE et al. *JAMA Network Open*

Sprycel	9711	9655	9863	10316	10316	10316	10378	10316	10316	10316	10316	10316
Stelara	7417	7417	7417	7417	7923	7923	7923	7923	7923	7923	8150	8150
Stribild	2498	2493	2493	2615	2615	2615	2615	2615	2615	2615	2615	2616
Sustiva	853	856	856	856	856	856	856	855	856	856	856	856
Sutent	12848	12848	12848	12848	12771	13430	13409	13410	13409	13361	13329	13385
Symbicort	279	280	280	280	280	280	280	280	280	280	280	280
Synthroid	31	31	31	31	31	31	32	32	32	32	32	32
Tasigna	9196	9196	9632	9748	9748	9748	9748	9713	9713	9690	9701	9690
Tecfidera	5185	5185	5307	5471	5471	5471	5471	5484	5766	5765	5765	5765
Tivicay	1251	1333	1336	1336	1337	1337	1337	1336	1337	1337	1337	1337
TNKase												4577
Tresiba											542	541
Trintellix												
Triumeq	2239	2239	2237	2237	2232	2233	2232	2232	2233	2321	2321	2320
Trulicity	497	496	496	496	496	536	540	540	540	541	541	544
Truvada	1302	1302	1302	1392	1392	1392	1392	1392	1392	1392	1392	1392
Tysabri	4834	4834	4834	4834	4834	4834	5119	5119	5119	5119	5119	5119
Varivax	113	113	113	113	110	111	105	104	102	105	109	110
Velcade	6429	6237	6252	6312	6252	6252	6279	6264	6279	6279	6279	6249
Viagra	212	212	212	212	212	232	232	232	232	232	232	232
Victoza	598	598	649	651	651	651	651	651	651	651	651	655
Viread	945	946	946	947	947	947	946	946	946	947	946	946
Vyvanse	221	221	221	221	221	221	221	232	232	232	232	232
Xarelto	320	319	318	318	318	318	318	335	337	337	337	337
Xeljanz	2542	2531	2531	2531	2531	2705	2701	2694	2689	2901	2902	2902
Xenazine	5807	5807	5807	5807	5807	6583	6593	6620	7547	7515	7498	7480
Xgeva	1776	1837	1842	1842	1842	1842	1842	1893	1893	1893	1893	1893
Xolair	1704	1704	1704	1764	1768	1768	1768	1768	1768	1835	1835	1835
Xtandi	8422	8422	8422	8887	8887	8887	8887	8887	8887	8887	8887	8887
Yervoy												33262
Zepatier												
Zetia	217	219	219	219	219	238	241	241	241	241	241	240
Zytiga	7285	7259	7259	7259	7772	7869	7868	7868	7868	7868	7868	7868

©2019 Wineinger NE et al. *JAMA Network Open*

2016

Product	2016-01	2016-02	2016-03	2016-04	2016-05	2016-06	2016-07	2016-08	2016-09	2016-10	2016-11	2016-12
Abilify Maintena	1724	1730	1727	1730	1727	1727	1811	1816	1804	1801	1804	1801
Actemra	3304	3325	3304	3304	3304	3304	3354	3360	3354	3364	3356	3342
Activase	132	134	133	133	133	129	138	135	139	137	137	138
Advair	337	337	337	337	337	337	337	336	336	336	336	336
Afinitor	11017	11220	11220	11220	11152	11921	11976	11994	11994	11994	11921	11907
Alimta	6288	6288	6288	6288	4341	6288	3397	3396	6411	3396	3396	6411
Androgel	479	479	479	479	479	481	526	526	526	525	526	524
Aranesp	724	740	742	744	748	745	742	743	744	742	739	739
Atripla	2410	2412	2412	2412	2412	2412	2412	2412	2412	2411	2411	2412
Aubagio	5491	5717	5717	5691	5691	5717	5701	5701	5717	5827	5854	5854
Avastin	748	748	719	748	737	726	735	734	746	734	737	574
Avonex	5453	5439	5435	5435	5439	5707	5707	5707	5707	5707	5665	5665
Baraclude	1253	1253	1249	1254	1313	1310	1310	1310	1314	1313	1314	1307
Botox	599	599	599	599	599	599	599	599	599	599	599	599
Breo Ellipta	301	301	301	301	301	301	300	300	300	300	300	300
Brilinta	312	312	312	312	312	312	312	312	310	310	310	310
Chantix	319	319	319	319	319	341	341	341	340	340	339	341
Cialis	278	278	278	278	278	312	314	314	314	314	314	335
Copaxone	5298	5298	5286	5286	5286	5286	5286	5286	5277	5286	5258	5258
Cosentyx	3664	3893	3916	3916	3916	3916	4048	4073	4073	4073	4048	4048
Creon	470	469	469	468	468	468	467	467	467	467	467	470
Crestor	251	251	251	251	252	252	251	251	250	250	250	250
Darzalex						7245	7245	7245	7230	7446	7458	7461
Descovy				1474	1478	1476	1473	1474	1470	1471	1471	1471
Eliquis	337	337	337	362	364	363	362	362	362	362	362	362
Enbrel	3669	3669	3658	3656	3656	3656	4017	4017	4017	4017	4003	4003
Epclusa							25195	25120	25009	24970	24821	24888
Epogen	641	647	650	649	653	649	653	646	683	653	653	653
Erbitux												
Exjade	7196	7287	7287	7287	7285	7781	7790	7785	7790	7771	7739	7762
Eylea	1877	1877	1877	1877	1877	1877	1877	1877	1877	1877	1877	1877

©2019 Wineinger NE et al. *JAMA Network Open*

Farxiga	368	368	367	390	396	396	396	396	396	396	396	396
Forteo	2333	2333	2333	2333	2333	2555	2555	2555	2555	2555	2555	2725
Gardasil 9	176	180	180	180	180	180	180	183	196	196	195	195
Genvoya	2599	2599	2599	2599	2598	2598	2599	2599	2598	2599	2599	2599
Gilenya	6082	6316	6319	6319	6319	6631	6635	6635	6635	6635	6625	6595
Gleevec	10265	10265	9839	9900	10077	9970	9961	10265	9961	10447	9961	9769
Harvoni	31185	31185	30996	31091	31066	31091	31185	31185	31185	31185	30977	30920
Herceptin	3999	3999	3999	3999	3999	3999	4116	4093	4116	4116	4116	4116
H.P. Acthar	33898	33898	33898	33081	33489	32861	33899	34249	33898	33081	34165	33898
Humalog	238	238	238	238	238	238	243	255	255	255	255	255
Humira	3462	3736	3736	3736	3736	3782	4032	4032	4032	4032	4015	4009
Humulin	128	128	128	128	128	128	130	138	138	138	138	137
Ibrance	10425	10425	10425	10388	10388	10388	10364	10369	10381	10381	10363	10364
Imbruvica	9687	9687	9687	10282	10356	10356	10356	10356	10324	10324	10324	10324
Invega Sustenna	1421	1423	1421	1421	1422	1421	1474	1477	1474	1475	1477	1477
Invega Trinza	4304	4204	4091	4091	4276	4047	4349	4356	3745	4321	4414	4205
Invokamet	368	368	368	396	397	397	397	397	396	396	396	396
Invokana	368	368	368	395	397	396	396	396	396	396	396	396
Isentress	1291	1299	1299	1299	1299	1299	1299	1299	1298	1298	1298	1298
Jadenu	10952	10990	10990	10969	10990	11683	11679	11680	11722	11680	11711	11679
Janumet	367	367	367	367	367	367	365	365	365	365	365	365
Januvia	366	366	366	366	366	366	365	365	364	364	364	365
Keytruda	8578	8429	8429	8706	8706	8554	8554	8554	8831	9116	8796	8911
Kyprolis	11249	11226	11226	11204	11526	11526	11526	11526				11959
Lantus	376	376	376	376	376	376	375	375	374	374	374	374
Levemir	407	407	407	407	407	407	407	407	406	406	406	407
Lexapro	255	255	255	255	255	255	278	278	278	276	277	276
Lipitor	211	211	211	211	211	231	231	231	230	230	230	230
Lovenox	979	778	285	390	994	654	995	979	990	919	1001	1001
Lucentis	2040	2040	2040	2040	2040	2040	2040	2040	2040	2040	2040	2040
Lupron Depot	990	988	987	1047	1055	1055	1052	1052	1047	1052	1052	1045
Lyrica	348	348	348	348	348	348	347	347	346	346	346	346
MMR2	79	79	81	81	81	81	81	79	83	85	83	84
Neulasta	4954	5114	5114	5114	5070	5070	5150	5314	5314	5314	5314	5335

Nexium	257	255	254	251	251	251	251	251	250	250	250	250
Northera	1727	1718	1717	1869	1886	1886	1886	1886	1886	1971	1971	1971
Novolog	462	462	462	462	462	462	495	498	498	498	498	498
NuvaRing	123	124	124	124	124	124	130	130	130	130	130	130
Odefsey			2360	2366	2366	2360	2366	2364	2364	2364	2366	2366
Onfi	770	770	872	878	878	878	878	878	875	914	914	914
Opdivo	9654	9495	9814	9636	9636	9976	9636	9636	9636	9779	9779	9779
Orencia	3443	3443	3443	3432	3432	3584	3584	3584	3584	3584	3571	3571
Orkambi	20082	20082	20082	20082	20082	20082	20082	20082	20082	20082	20082	20082
Otezla	2433	2439	2439	2615	2626	2626	2624	2624	2624	2624	2618	2618
Perjeta	4376	4384	4376	4380	4380	4376						
Plavix	196	196	196	196	196	196	196	195	195	195	195	195
Pneumovax	93	93	90	90	90	90	90	90	98	102	102	102
Pomalyst	12104	12104	12782	12927	12927	12927	13006	13163	13356	13380	13235	13315
Premarin	132	132	132	132	132	144	144	144	144	144	144	144
Prevnar 13	179	179	179	179	179	179	179	179	179	179	189	189
Prezista	1360	1362	1363	1363	1364	1362	1360	1361	1366	1369	1369	1369
Prolia	1009	1054	1054	1054	1054	1054	1054	1082	1082	1082	1082	1082
ProQuad	184	182	182	182	182	116	182	183	192		191	
Pulmicort	202	202	202	202	202	202	202	202	202	202	202	202
Remicade	3985	3985	4180	4180	4180	4180	4180	4180	4189	4343	4343	4343
Renagel	587	587	642	643	643	644	643	642	642	643	643	642
Renvela	467	467	513	514	514	514	514	514	513	513	513	513
Restasis	431	431	431	431	431	431	429	429	429	428	428	429
Revlimid	10388	10388	11022	11094	11094	11094	11094	11229	11427	11427	11427	11427
Rexulti	873	873	873	943	943	943	943	943	943	943	943	943
Rituxan	15435	15435	15435	15435	15435	15435	16011	16011	16011	16011	16011	16011
Sabril	13157	13210	13157	13157	13157	13191	13231	13292	13292	13237	13316	13157
Sandostatin	3292	3291	3291	3314	3324	3284	3438	3476	3460	3493	3498	3502
Saxenda	1084	1084	1084	1164	1168	1171	1168	1168	1160	1158	1158	1159
Sensipar	696	704	702	700	700	700	749	756	756	756	756	756
Simponi	3476	3486	3759	3796	3782	3782	3782	3782	3782	3782	3769	3759
Soliris	18239	9180	18676	18676	18676	18676	18806		19140			
Spinraza												

©2019 Wineinger NE et al. *JAMA Network Open*

Sprycel	10782	10816	10801	10788	10788	10788	11213	11213	11213	11213	11119	11084
Stelara	8150	8150	8150	8545	8545	8545	8545	8545	8545	8545	8545	8545
Stribild	2727	2727	2727	2727	2727	2727	2915	2915	2915	2915	2915	2915
Sustiva	933	934	934	934	934	933	934	934	932	932	933	933
Sutent	13910	14029	13868	13825	14080	14517	14784	14517	14624	14606	14517	14517
Symbicort	293	295	294	294	294	294	293	293	293	293	293	293
Synthroid	34	34	34	34	34	34	34	34	33	33	33	34
Tasigna	10166	10346	10310	10296	10296	10439	10502	10502	10519	10535	10439	10439
Tecfidera	5968	5968	5932	5932	5968	6229	6229	6229	6229	6229	6157	6157
Tivicay	1330	1435	1435	1435	1434	1434	1435	1435	1434	1435	1435	1434
TNKase	4989											
Tresiba	538	539	539	539	538	538	538	538	538	538	538	538
Trintellix					322	322	322	322	321	321	321	321
Triumeq	2314	2428	2427	2427	2427	2427	2427	2427	2427	2427	2427	2426
Trulicity	582	582	580	580	580	580	580	586	632	632	632	632
Truvada	1479	1479	1479	1479	1479	1479	1479	1479	1479	1479	1479	1479
Tysabri	5370	5370	5370	5370	5370	5370	5634	5634	5634	5634	5634	5634
Varivax	108	109	109	111	109	112	112	110	116	116	116	118
Velcade	6257	6257	6257	6257	6257	6257	6222	6222	6252	6252	6252	6252
Viagra	260	260	260	259	260	293	293	293	291	291	291	292
Victoza	699	699	699	699	699	699	699	697	748	750	750	750
Viread	1007	1007	1007	1007	1007	1007	1007	1007	1006	1004	1003	1005
Vyvanse	249	251	251	251	251	251	251	251	250	250	250	250
Xarelto	335	335	335	358	361	361	360	360	360	360	360	360
Xeljanz	3176	3176	3176	3167	3167	3454	3455	3454	3463	3463	3445	3445
Xenazine	8339	8474	8474	8527	8501	8579	8545	8501	8501	8563	8501	8501
Xgeva	1893	1963	1963	1963	1959	1963	1952	2027	2034	2036	2036	1940
Xolair	1835	1835	1835	1905	1905	1905	1905	1905	1905	1978	1978	1978
Xtandi	8813	8840	8813	8813	8786	8887	9322	9220	9171	9231	9220	9220
Yervoy									33722			
Zepatier		18292	18346	18296	18128	18216	18128	18127	18127	18127	18127	18142
Zetia	260	262	262	262	262	263	287	287	286	286	286	287
Zytiga	7820	7820	8342	8438	8438	8438	8432	8438	8397	8435	8386	8386

©2019 Wineinger NE et al. *JAMA Network Open*

2017

Product	2017-01	2017-02	2017-03	2017-04	2017-05	2017-06	2017-07	2017-08	2017-09	2017-10	2017-11	2017-12
Abilify Maintena	1881	1882	1885	1876	1885	1884	1975	1975	1969	1969	1969	1975
Actemra	3609	3609	3609	3600	3600	3600	3654	3658	3654	3656	3654	3654
Activase	142	149	143	146		141	149	148	160	148	152	153
Advair	361	361	361	361	361	361	361	361	361	360	360	360
Afinitor	12590	12590	12606	12590	12628	12873	12982	12942	12950	12942	12903	12942
Alimta				6494	6485	6494	6607	6622	6622	6667	6593	6622
Androgel	521	521	521	566	567	566	566	566	566	566	566	566
Aranesp	762	770	769	768	768	773	775	770	768	773	793	767
Atripla	2438	2516	2539	2539	2539	2537	2539	2539	2531	2531	2531	2531
Aubagio	5836	5836	5836	5836	5827	5832	5827	5827	5827	6109	6118	6118
Avastin	413	753	758	758	758	758	774	774	774	774	771	627
Avonex	6119	6119	6119	6119	6119	6119	6111	6111	6111	6111	6111	6111
Baraclude	1386	1388	1388	1388	1388	1384	1384	1379	1387	1384	1379	1377
Botox	599	599	599	599	599	599	620	620	620	620	620	620
Breo Ellipta	321	321	321	321	321	321	321	321	321	322	322	322
Brilinta	334	334	334	334	334	334	333	333	333	333	333	333
Chantix	369	369	369	368	369	392	392	392	392	392	392	392
Cialis	333	332	332	332	365	365	365	365	365	365	365	365
Copaxone	5669	5669	5669	5669	5669	5668	5668	5668	5668	5668	5662	5655
Cosentyx	4048	4048	4291	4291	4291	4291	4382	4407	4407	4407	4407	4407
Creon	487	487	487	487	487	487	487	486	487	486	486	487
Crestor	261	261	261	261	262	261	261	261	261	261	261	261
Darzalex	7461	7676	7646	7676	7676	7676	7676	7676	7737	7832	7832	7899
Descovy	1461	1548	1557	1556	1556	1556	1556	1556	1556	1556	1556	1556
Eliquis	389	389	389	388	388	388	388	388	388	388	388	388
Enbrel	4070	4339	4339	4339	4339	4339	4339	4339	4339	4334	4334	4334
Epclusa	24820	24820	24820	24820	24747	24820	24820	24820	24806	24820	24820	24820
Epogen	687	678	690	676	675	672	667	680	676	731	731	731
Erbitux												
Exjade	8236	8223	8236	8254	8239	8398	8448	8448	8479	8453	8485	8479
Eylea	1877	1877	1877	1877	1877	1877	1877	1877	1877	1877	1877	1877

©2019 Wineinger NE et al. *JAMA Network Open*

Farxiga	426	431	431	431	431	431	431	431	431	431	431	431
Forteo	2725	2725	2725	2725	2986	2986	2986	2986	2986	2986	2986	3088
Gardasil 9	195	195	196	195	194	197	196	204	206	205	202	206
Genvoya	2560	2719	2734	2734	2734	2734	2734	2734	2734	2734	2734	2734
Gilenya	6595	6956	7016	6982	6991	6961	7183	7218	7240	7209	7209	7209
Gleevec	9985	9778	9961	9961	9961	10265	1244	1244	8821			
Harvoni	30920	30920	30939	30920	30920	30920	30920	30977	30977	30920	30920	30920
Herceptin	4237	4237	4237	4237	4237	4237	4361	4361	4361			
H.P. Acthar	34306	36520	36368	36444	36236	36236	36444	36542	36542	36577	36673	36236
Humalog	254	254	254	254	274	274	274	274	274	274	274	274
Humira	4118	4338	4344	4338	4338	4338	4338	4338	4338	4338	4338	4338
Humulin	137	136	136	136	138	145	147	146	146	146	146	146
Ibrance	10920	10932	10920	10919	10919	10919	10887	10919	10887	10887	10886	10887
Imbruvica	10499	11152	11152	11152	11152	11152	11152	11152	11152	11152	11152	11152
Invega Sustenna	1508	1536	1535	1530	1532	1531	1570	1582	1580	1580	1584	1587
Invega Trinza	4319	4530	4557	4587	4503	4536	4641	4702	4702	4579	4702	4671
Invokamet	392	421	427	427	427	427	427	427	427	427	427	427
Invokana	392	422	427	427	427	427	427	427	427	427	427	427
Isentress	1370	1381	1381	1381	1381	1381	1381	1381	1381	1379	1379	1379
Jadenu	12449	12484	12484	12484	12491	12728	12757	12796	12756	12756	12757	12756
Janumet	378	381	381	381	381	392	397	397	397	397	397	397
Januvia	376	380	380	380	380	392	397	397	397	396	396	396
Keytruda	8681	8681	8759	8810	8810	8810	8810	8810	8839	8940	8940	8940
Kyprolis	11827	11827	11827	11827	12286							12919
Lantus	373	373	373	373	373	373	373	373	373	384	384	384
Levemir	403	403	403	403	403	403	403	403	403	403	403	403
Lexapro	300	300	300	300	300	300	300	300	300	300	300	300
Lipitor	251	251	251	251	251	274	274	274	274	274	274	274
Lovenox	850	927	970	942	803	640	463	807	807	560	361	884
Lucentis	2040	2040	2040	2040	2040	2040						
Lupron Depot	1062	1052	1050	1115	1125	1116	1124	1124	1122	1117	1117	1115
Lyrica	377	377	377	376	376	411	412	411	411	411	411	411
MMR2	89	89	88	84	84	83	83	83	85	79	78	85
Neulasta	5463	5570	5570	5570	5570	5570	5617	5828	5828	5828	5817	5828

©2019 Wineinger NE et al. *JAMA Network Open*

Nexium	251	252	252	252	252	252	252	252	252	252	252	252
Northera	1971	1971	1970	2136	2165	2146	2153	2150	2139	2146	2146	2139
Novolog	494	494	532	532	532	532	532	532	532	532	532	532
NuvaRing	136	136	136	136	136	140	141	141	141	141	141	141
Odefsey	2336	2483	2491	2488	2488	2488	2488	2488	2488	2488	2488	2488
Onfi	909	909	909	993	997	996	996	996	996	996	996	996
Opdivo	9869	9885	10110	10201	10078	10078	10003	9969	9947	10119	10095	10072
Orencia	3786	3790	3790	3786	3786	3777	3786	3786	3786	3781	3777	3777
Orkambi	20023	20023	20023	20023	20023	20023	21008	20949	20949	20836	20836	20836
Otezla	2764	2788	2788	2971	2978	2978	2978	2978	2978	3106	3118	3118
Perjeta	4637	4614	4637	4637	4637	4637	4773	4773		4751	4760	4773
Plavix	194	194	194	194	194	194	194	194	194	194	194	194
Pneumovax	102	102	100	101	101	98	98	105	109	109	109	109
Pomalyst	13235	13276	13222	14209	14381	14424	14381	14359	14381	14642	15504	15480
Premarin	156	156	156	156	156	156	156	156	156	156	156	156
Prevnar 13	189	189	189	189	189	189	189	189	182	182	185	193
Prezista	1406	1459	1455	1454	1454	1454	1454	1454	1454	1454	1454	1454
Prolia	1082	1131	1131	1131	1131	1131	1131	1180	1180	1180	1180	1180
ProQuad	192	191	192	111	192	179	192	198	203	203	203	
Pulmicort	216	216	216	216	216	216	216	216	216	216	216	216
Remicade	4343	4528	4556	4556	4556	4556	4556	4556	4556	4556	4556	4556
Renagel	635	635	635	635	635	635	635	635	633	635	635	634
Renvela	508	508	508	508	508	508	508	507	506	506	505	501
Restasis	463	464	463	463	463	463	464	463	463	463	463	463
Revlimid	12250	12341	12341	12341	12341	12341	12463	12511	12511	12565	13637	13637
Rexulti	935	932	932	1006	1006	1007	1006	1007	1006	1006	1006	1007
Rituxan	16647	16647	16647	16647	16647	16647	17310	17310	17310	17310	17310	17310
Sabril	14355	14480	14459	14501	14459	14459	14459	14372	14372	14372	14372	14372
Sandostatin	3475	3498	3074		3498	3498						
Saxenda	1154	1154	1154	1154	1154	1154	1154	1154	1154	1154	1154	1154
Sensipar	807	812	812	810	811	812	811	811	810	810	810	810
Simponi	3759	4034	4104	4094	4084	4084	4094	4094	4084	4084	4084	4094
Soliris											19487	
Spinraza			131250	130500	130500	128250	126390	129000	126860	123860	123860	125930

©2019 Wineinger NE et al. *JAMA Network Open*

Sprycel	11828	11849	11828	11849	11849	11828	11838	11824	11813	11758	11755	11758
Stelara	8545	9213	9213	9213	9213	9213	9213	9213	9213	9213	9213	9213
Stribild	2888	2880	2880	2880	2880	2871	3069	3078	3069	3069	3065	3069
Sustiva	978	978	978	978	977	975	975	973	975	973	973	973
Sutent	15168	15242	15256	15256	15467	15778	15999	15778	16018	15979	15778	15855
Symbicort	308	308	308	308	308	308	308	308	308	308	308	308
Synthroid	34	34	34	34	34	34	34	34	34	34	34	35
Tasigna	11098	11127	11125	11098	11125	11283	11409	11409	11368	11349	11347	11409
Tecfidera	6650	6650	6650	6650	6637	6637	6645	6629	6629	6629	6629	6629
Tivicay	1422	1530	1530	1530	1530	1529	1530	1530	1526	1526	1526	1526
TNKase										5389		5389
Tresiba	533	533	533	533	533	533	533	533	533	533	533	533
Trintellix	346	347	347	347	347	347	347	347	347	347	347	347
Triumeq	2406	2590	2589	2582	2582	2578	2581	2581	2578	2578	2578	2578
Trulicity	626	626	626	626	676	676	676	676	676	676	676	674
Truvada	1461	1546	1561	1558	1558	1557	1557	1558	1557	1557	1556	1557
Tysabri	5828	5828	5828	5828	5828	5828	5828	5828	5828	5828	5828	5828
Varivax	117	120	120	117	121	123	118	124	124	122	121	123
Velcade	6252	6252	6252	6252	6312	6252	6252	6252	6252	6252	6282	6319
Viagra	328	328	328	328	328	370	370	370	370	370	370	370
Victoza	748	748	748	747	747	757	806	805	805	805	805	805
Viread	995	1052	1063	1063	1063	1060	1063	1063	1059	1059	1059	1057
Vyvanse	271	271	271	271	271	271	271	270	270	270	270	270
Xarelto	358	365	386	386	386	386	386	386	386	386	386	386
Xeljanz	3758	3767	3758	3758	3758	3758	3758	3758	3758	3757	3757	3757
Xenazine	9343	9309	9456	9329	9309	9292	9275	9275	9236	9264	9174	9309
Xgeva	2027	2093	2032	2080	2061	2089	2055	2094	2094	2098	2151	2140
Xolair	1976	1976	1975	2053	2053	2053	2053	2053	2053	2118	2132	2132
Xtandi	9675	9675	9675	9675	9675	9914	10038	10038	10038	10038	10038	10038
Yervoy			34450	34960	34960	34600	34600	34600	34743	35118	35118	35118
Zepatier	17909	17909	17855	17822	17679	17669	17669	17669	17669	17647	17647	17647
Zetia	313	314	314	314	314	314	314	314	314	311	312	313
Zytiga	8386	9076	9133	9076	9133	9133	9133	9133	9133	9133	9133	9133

©2019 Wineinger NE et al. *JAMA Network Open*

eTable 3: Median monthly costs for 49 products included in main report. Includes same information on these products as in eTable 2.

2012

Product	2012-01	2012-02	2012-03	2012-04	2012-05	2012-06	2012-07	2012-08	2012-09	2012-10	2012-11	2012-12
Advair	225	241	241	241	241	241	241	241	240	250	250	250
Androgel	321	322	322	322	322	322	322	322	322	321	321	321
Atripla	1776	1784	1784	1782	1784	1784	1784	1804	1807	1807	1807	1807
Brilinta	236	238	238	238	238	238	238	238	238	237	238	237
Chantix	175	175	175	175	175	175	190	190	190	190	190	190
Cialis	127	139	139	139	139	139	149	151	151	151	151	151
Creon	293	294	294	294	294	320	320	320	320	320	320	320
Crestor	146	146	146	146	146	146	157	157	157	157	157	157
Eliquis												
Enbrel	1862	1950	1950	1950	1950	1959	1990	2094	2094	2094	2094	2094
Farxiga												
Forteo	1116	1116	1116	1116	1116	1116	1116	1116	1116	1217	1217	1217
Harvoni												
Humalog	126	126	126	126	126	126	132	134	134	134	134	134
Humira	1940	1949	1940	1940	1940	1940	2074	2077	2077	2074	2077	2074
Humulin	67	67	67	67	67	67	68	73	73	73	73	73
Invokana												
Isentress	1005	1005	1014	1054	1054	1054	1054	1054	1054	1054	1054	1054
Januvia	219	219	219	219	219	220	230	230	230	230	230	230
Lantus	212	211	212	212	218	218	218	218	218	234	235	235
Lexapro	120	129	130	130	130	130	141	141	141	142	142	142
Lipitor	116	116	116	116	116	116	121	121	121	121	121	121
Lyrica	174	174	174	174	174	174	189	189	189	189	189	189
Nexium	188	188	188	188	188	188	194	194	194	194	194	194
Novolog	244	244	244	244	244	244	244	263	263	263	263	264
Onfi												
Orencia												
Otezla												
Premarin	68	68	68	68	68	68	74	74	74	74	74	74

©2019 Wineinger NE et al. *JAMA Network Open*

Prezista											1055	1055
Pulmicort	151	152	152	152	152	151	156	156	156	156	156	156
Renvela	212	228	228	228	228	228	228	228	228	228	228	228
Restasis	266	266	266	274	279	279	279	279	279	279	279	279
Simponi	1978	2114	2114	2114	2114	2114	2101	2238	2238	2238	2238	2246
Stelara	5420	5680	5680	5680	5680	5680	5680	5953	5953	5953	5953	5953
Stribild								2391	2409	2409	2409	2409
Symbicort	225	225	225	225	225	225	225	225	225	225	225	225
Synthroid	20	20	20	20	20	20	20	20	20	20	20	23
Tivicay												
Triumeq												
Trulicity												
Truvada	1188	1190	1188	1188	1193	1190	1193	1193	1193	1193	1193	1193
Viagra	127	127	127	127	127	127	139	139	139	139	139	139
Victoza	433	433	433	433	433	433	433	468	467	467	467	467
Viread	746	746	746	802	805	805	805	802	801	804	804	804
Vyvanse	162	162	162	162	162	162	162	162	162	162	162	162
Xarelto	225	225	225	225	225	225	238	238	238	238	238	238
Xeljanz											2098	2100
Zetia	126	126	129	138	138	138	138	139	146	146	146	146

©2019 Wineinger NE et al. *JAMA Network Open*

2013

Product	2013-01	2013-02	2013-03	2013-04	2013-05	2013-06	2013-07	2013-08	2013-09	2013-10	2013-11	2013-12
Advair	250	267	267	267	267	268	275	276	275	275	276	275
Androgel	352	353	353	353	353	353	353	353	353	387	388	388
Atripla	1926	1926	1926	1926	1926	1926	1926	1926	1926	1926	1926	1926
Brilinta	244	244	244	244	244	244	244	244	244	244	244	244
Chantix	208	208	208	208	208	208	218	218	218	218	218	218
Cialis	165	165	165	165	165	165	180	180	180	180	180	181
Creon	350	351	351	351	351	351	351	351	351	351	351	351
Crestor	166	166	166	166	166	166	176	176	176	176	176	176
Eliquis	258	257	257	257	257	257	257	257	272	272	272	272
Enbrel	2222	2229	2229	2229	2229	2238	2382	2382	2382	2382	2378	2378
Farxiga												
Forteo	1217	1217	1217	1217	1255	1326	1326	1326	1326	1326	1326	1344
Harvoni												
Humalog	144	144	144	144	144	144	144	157	157	157	157	165
Humira	2194	2214	2214	2214	2214	2214	2351	2360	2353	2353	2353	2370
Humulin	78	78	78	78	78	83	85	85	85	85	84	86
Invokana				271	271	271	271	271	271	270	270	270
Isentress	1051	1055	1102	1102	1102	1102	1102	1102	1102	1102	1102	1102
Januvia	251	253	253	253	253	253	253	265	265	265	265	265
Lantus	235	235	235	235	258	258	258	282	283	283	283	298
Lexapro	154	154	154	154	154	154	168	168	168	168	168	168
Lipitor	132	132	132	132	132	132	144	144	144	144	144	144
Lyrica	207	207	207	207	207	207	226	226	226	226	226	226
Nexium	205	205	205	205	205	205	230	230	230	230	230	230
Novolog	281	281	281	281	281	281	282	304	303	303	304	333
Onfi												457
Orencia											2430	2423
Otezla												
Premarin	81	81	81	81	81	81	88	88	88	88	88	88
Prezista	1119	1119	1119	1119	1119	1119	1119	1119	1119	1119	1119	1119

©2019 Wineinger NE et al. *JAMA Network Open*

Pulmicort	165	165	165	165	165	166	166	166	165	165	165	165
Renvela	228	230	246	246	246	266	268	268	268	270	294	294
Restasis	290	292	292	292	292	307	307	307	307	307	307	307
Simponi	2245	2392	2401	2401	2401	2401	2400	2557	2567	2566	2566	2566
Stelara	5917	6317	6317	6317	6317	6317	6317	6745	6745	6745	6745	6745
Stribild	2402	2402	2402	2402	2402	2402	2402	2402	2402	2402	2402	2402
Symbicort	242	242	242	242	242	242	242	242	242	242	242	242
Synthroid	24	24	23	23	23	23	25	25	25	25	25	25
Tivicay								1190	1206	1205	1206	1206
Triumeq												
Trulicity												
Truvada	1255	1255	1255	1255	1255	1255	1255	1255	1255	1255	1255	1255
Viagra	152	151	151	151	151	151	165	166	166	166	166	165
Victoza	467	467	466	471	504	504	504	504	504	504	504	547
Viread	850	853	853	852	851	853	854	854	852	852	853	851
Vyvanse	176	176	176	176	176	176	189	189	189	189	189	189
Xarelto	256	257	257	257	257	257	257	271	272	272	272	272
Xeljanz	2108	2108	2116	2108	2114	2114	2108	2107	2110	2108	2109	2108
Zetia	146	147	161	161	161	161	161	168	168	168	168	168

©2019 Wineinger NE et al. *JAMA Network Open*

2014

Product	2014-01	2014-02	2014-03	2014-04	2014-05	2014-06	2014-07	2014-08	2014-09	2014-10	2014-11	2014-12
Advair	289	291	291	290	290	290	290	290	290	290	290	290
Androgel	386	386	386	386	386	386	412	412	412	412	412	412
Atripla	2033	2043	2041	2038	2038	2082	2084	2084	2084	2084	2084	2084
Brilinta	250	250	250	250	250	250	252	258	258	258	258	257
Chantix	231	231	231	231	231	252	253	252	253	253	253	253
Cialis	195	195	195	195	195	195	212	213	213	213	213	233
Creon	384	384	384	383	384	383	420	421	421	421	421	421
Crestor	186	186	186	186	186	186	191	191	191	191	191	191
Eliquis	271	271	276	297	297	297	297	297	297	297	297	312
Enbrel	2539	2547	2547	2547	2547	2690	2722	2722	2722	2690	2788	2903
Farxiga												320
Forteo	1446	1447	1451	1447	1448	1446	1576	1577	1576	1576	1576	1789
Harvoni										31980	31942	31942
Humalog	172	172	172	171	171	187	188	188	188	188	189	207
Humira	2515	2515	2515	2515	2509	2508	2706	2706	2706	2706	2801	2919
Humulin	92	92	92	92	92	95	100	100	100	100	100	110
Invokana	295	296	296	296	296	296	296	318	319	319	319	319
Isentress	1148	1150	1150	1150	1149	1147	1145	1145	1145	1145	1145	1145
Januvia	290	290	290	290	289	289	289	306	306	306	306	306
Lantus	310	310	310	310	310	340	340	340	340	340	375	380
Lexapro	181	182	182	182	181	181	197	197	198	198	198	198
Lipitor	156	156	156	156	156	170	170	170	170	170	170	170
Lyrica	246	246	246	246	246	269	269	269	269	269	269	269
Nexium	242	242	242	242	242	242	242	242	242	242	241	241
Novolog	332	332	332	331	332	364	364	364	364	364	367	400
Onfi	496	496	495	496	496	496	496	496	496	496	514	570
Orencia	2482	2558	2558	2558	2557	2555	2729	2731	2731	2707	2707	2921
Otezla				1932	1931	1929	1922	1922	1920	1917	1914	1913
Premarin	93	93	93	93	93	102	102	102	102	102	102	102
Prezista	1182	1190	1190	1188	1187	1185	1183	1183	1184	1184	1183	1183

©2019 Wineinger NE et al. *JAMA Network Open*

Pulmicort	174	175	174	174	174	174	174	174	174	174	174	174
Renvela	294	312	323	322	322	354	354	354	354	354	388	389
Restasis	320	326	326	326	326	326	332	336	336	336	336	336
Simponi	2566	2733	2743	2741	2743	2739	2739	2994	3012	2994	2994	2994
Stelara	6704	7158	7158	7158	7158	7158	7158	7158	7158	7158	7433	7433
Stribild	2505	2508	2507	2507	2505	2500	2500	2500	2500	2499	2500	2499
Symbicort	261	261	261	261	260	260	260	261	261	261	261	260
Synthroid	27	27	27	27	27	27	30	30	30	30	30	30
Tivicay	1200	1259	1259	1258	1258	1257	1254	1254	1253	1253	1254	1253
Triumeq								2232	2252	2244	2244	2242
Trulicity											499	499
Truvada	1304	1310	1310	1309	1309	1306	1304	1304	1304	1304	1304	1304
Viagra	175	175	175	175	175	195	195	195	195	195	195	195
Victoza	548	548	548	548	548	547	600	601	601	601	601	601
Viread	887	892	892	892	892	892	887	887	887	887	887	887
Vyvanse	204	204	204	204	204	204	204	204	204	204	204	204
Xarelto	271	271	289	292	291	292	291	291	292	292	291	318
Xeljanz	2252	2262	2263	2262	2263	2417	2401	2401	2401	2397	2401	2397
Zetia	169	189	189	189	189	189	189	200	200	200	200	200

©2019 Wineinger NE et al. *JAMA Network Open*

2015

Product	2015-01	2015-02	2015-03	2015-04	2015-05	2015-06	2015-07	2015-08	2015-09	2015-10	2015-11	2015-12
Advair	300	303	303	303	303	303	315	315	315	315	315	315
Androgel	439	439	439	439	482	482	482	482	482	482	482	482
Atripla	2155	2157	2156	2245	2247	2247	2247	2247	2245	2247	2247	2246
Brilinta	276	277	277	277	277	277	290	290	290	290	290	290
Chantix	276	276	276	276	276	293	293	293	293	293	293	293
Cialis	232	232	232	232	236	255	255	255	255	255	255	281
Creon	448	448	447	449	448	448	448	448	448	448	448	450
Crestor	209	210	210	210	210	210	220	220	220	220	220	220
Eliquis	319	319	319	319	319	319	319	322	338	338	338	338
Enbrel	2903	2903	2903	2903	3171	3190	3179	3171	3400	3410	3409	3421
Farxiga	318	325	349	349	349	349	349	349	349	368	369	369
Forteo	1788	1788	1788	1788	1788	2042	2042	2042	2042	2042	2042	2333
Harvoni	31752	31752	31564	31563	31563	31563	31563	31563	31563	31563	31450	31399
Humalog	204	204	204	204	204	224	224	224	224	224	224	238
Humira	2919	2902	2902	3189	3189	3189	3189	3189	3441	3441	3433	3433
Humulin	105	110	110	110	110	121	121	121	121	121	121	129
Invokana	318	348	349	349	349	349	349	349	368	369	369	369
Isentress	1218	1221	1221	1222	1222	1222	1221	1221	1221	1222	1222	1221
Januvia	332	336	336	336	336	336	336	336	336	335	338	368
Lantus	379	379	379	379	379	379	379	379	379	379	379	379
Lexapro	214	214	214	214	215	214	238	238	238	238	238	238
Lipitor	184	184	184	184	184	193	193	193	193	193	193	193
Lyrica	293	293	293	293	293	320	320	320	320	320	320	320
Nexium	241	241	241	241	242	255	255	255	255	256	257	257
Novolog	399	399	399	399	403	439	439	439	439	439	439	465
Onfi	568	568	646	646	646	646	646	646	766	775	775	775
Orencia	2921	2917	2915	2915	2915	3200	3204	3200	3200	3200	3190	3430
Otezla	1913	1913	2047	2063	2063	2063	2260	2259	2259	2259	2259	2259
Premarin	111	111	111	111	111	122	122	122	122	122	122	122
Prezista	1272	1273	1273	1277	1277	1277	1273	1273	1273	1275	1272	1273

©2019 Wineinger NE et al. *JAMA Network Open*

Pulmicort	185	185	186	186	187	187	189	188	189	188	189	188
Renvela	388	388	427	427	427	427	427	427	469	470	470	470
Restasis	361	361	361	361	362	395	395	395	395	396	395	395
Simponi	2980	2980	3261	3271	3271	3271	3271	3261	3473	3496	3490	3486
Stelara	7417	7417	7417	7417	7923	7923	7923	7923	7923	7923	8150	8150
Stribild	2498	2493	2493	2615	2615	2615	2615	2615	2615	2615	2615	2616
Symbicort	279	280	280	280	280	280	280	280	280	280	280	280
Synthroid	31	31	31	31	31	31	32	32	32	32	32	32
Tivicay	1251	1333	1336	1336	1337	1337	1337	1336	1337	1337	1337	1337
Triumeq	2239	2239	2237	2237	2232	2233	2232	2232	2233	2321	2321	2320
Trulicity	497	496	496	496	496	536	540	540	540	541	541	544
Truvada	1302	1302	1302	1392	1392	1392	1392	1392	1392	1392	1392	1392
Viagra	212	212	212	212	212	232	232	232	232	232	232	232
Victoza	598	598	649	651	651	651	651	651	651	651	651	655
Viread	945	946	946	947	947	947	946	946	946	947	946	946
Vyvanse	221	221	221	221	221	221	221	232	232	232	232	232
Xarelto	320	319	318	318	318	318	318	335	337	337	337	337
Xeljanz	2542	2531	2531	2531	2531	2705	2701	2694	2689	2901	2902	2902
Zetia	217	219	219	219	219	238	241	241	241	241	241	240

©2019 Wineinger NE et al. *JAMA Network Open*

2016

Product	2016-01	2016-02	2016-03	2016-04	2016-05	2016-06	2016-07	2016-08	2016-09	2016-10	2016-11	2016-12
Advair	337	337	337	337	337	337	337	336	336	336	336	336
Androgel	479	479	479	479	479	481	526	526	526	525	526	524
Atripla	2410	2412	2412	2412	2412	2412	2412	2412	2412	2411	2411	2412
Brilinta	312	312	312	312	312	312	312	312	310	310	310	310
Chantix	319	319	319	319	319	341	341	341	340	340	339	341
Cialis	278	278	278	278	278	312	314	314	314	314	314	335
Creon	470	469	469	468	468	468	467	467	467	467	467	470
Crestor	251	251	251	251	252	252	251	251	250	250	250	250
Eliquis	337	337	337	362	364	363	362	362	362	362	362	362
Enbrel	3669	3669	3658	3656	3656	3656	4017	4017	4017	4017	4003	4003
Farxiga	368	368	367	390	396	396	396	396	396	396	396	396
Forteo	2333	2333	2333	2333	2333	2555	2555	2555	2555	2555	2555	2725
Harvoni	31185	31185	30996	31091	31066	31091	31185	31185	31185	31185	30977	30920
Humalog	238	238	238	238	238	238	243	255	255	255	255	255
Humira	3462	3736	3736	3736	3736	3782	4032	4032	4032	4032	4015	4009
Humulin	128	128	128	128	128	128	130	138	138	138	138	137
Invokana	368	368	368	395	397	396	396	396	396	396	396	396
Isentress	1291	1299	1299	1299	1299	1299	1299	1299	1298	1298	1298	1298
Januvia	366	366	366	366	366	366	365	365	364	364	364	365
Lantus	376	376	376	376	376	376	375	375	374	374	374	374
Lexapro	255	255	255	255	255	255	278	278	278	276	277	276
Lipitor	211	211	211	211	211	231	231	231	230	230	230	230
Lyrica	348	348	348	348	348	348	347	347	346	346	346	346
Nexium	257	255	254	251	251	251	251	251	250	250	250	250
Novolog	462	462	462	462	462	462	495	498	498	498	498	498
Onfi	770	770	872	878	878	878	878	878	875	914	914	914
Orencia	3443	3443	3443	3432	3432	3584	3584	3584	3584	3584	3571	3571
Otezla	2433	2439	2439	2615	2626	2626	2624	2624	2624	2624	2618	2618
Premarin	132	132	132	132	132	144	144	144	144	144	144	144
Prezista	1360	1362	1363	1363	1364	1362	1360	1361	1366	1369	1369	1369

©2019 Wineinger NE et al. *JAMA Network Open*

Pulmicort	202	202	202	202	202	202	202	202	202	202	202	202
Renvela	467	467	513	514	514	514	514	514	513	513	513	513
Restasis	431	431	431	431	431	431	429	429	429	428	428	429
Simponi	3476	3486	3759	3796	3782	3782	3782	3782	3782	3782	3769	3759
Stelara	8150	8150	8150	8545	8545	8545	8545	8545	8545	8545	8545	8545
Stribild	2727	2727	2727	2727	2727	2727	2915	2915	2915	2915	2915	2915
Symbicort	293	295	294	294	294	294	293	293	293	293	293	293
Synthroid	34	34	34	34	34	34	34	34	33	33	33	34
Tivicay	1330	1435	1435	1435	1434	1434	1435	1435	1434	1435	1435	1434
Triumeq	2314	2428	2427	2427	2427	2427	2427	2427	2427	2427	2427	2426
Trulicity	582	582	580	580	580	580	580	586	632	632	632	632
Truvada	1479	1479	1479	1479	1479	1479	1479	1479	1479	1479	1479	1479
Viagra	260	260	260	259	260	293	293	293	291	291	291	292
Victoza	699	699	699	699	699	699	699	697	748	750	750	750
Viread	1007	1007	1007	1007	1007	1007	1007	1007	1006	1004	1003	1005
Vyvanse	249	251	251	251	251	251	251	251	250	250	250	250
Xarelto	335	335	335	358	361	361	360	360	360	360	360	360
Xeljanz	3176	3176	3176	3167	3167	3454	3455	3454	3463	3463	3445	3445
Zetia	260	262	262	262	262	263	287	287	286	286	286	287

©2019 Wineinger NE et al. *JAMA Network Open*

2017

Product	2017-01	2017-02	2017-03	2017-04	2017-05	2017-06	2017-07	2017-08	2017-09	2017-10	2017-11	2017-12
Advair	361	361	361	361	361	361	361	361	361	360	360	360
Androgel	521	521	521	566	567	566	566	566	566	566	566	566
Atripla	2438	2516	2539	2539	2539	2537	2539	2539	2531	2531	2531	2531
Brilinta	334	334	334	334	334	334	333	333	333	333	333	333
Chantix	369	369	369	368	369	392	392	392	392	392	392	392
Cialis	333	332	332	332	365	365	365	365	365	365	365	365
Creon	487	487	487	487	487	487	487	486	487	486	486	487
Crestor	261	261	261	261	262	261	261	261	261	261	261	261
Eliquis	389	389	389	388	388	388	388	388	388	388	388	388
Enbrel	4070	4339	4339	4339	4339	4339	4339	4339	4339	4334	4334	4334
Farxiga	426	431	431	431	431	431	431	431	431	431	431	431
Forteo	2725	2725	2725	2725	2986	2986	2986	2986	2986	2986	2986	3088
Harvoni	30920	30920	30939	30920	30920	30920	30920	30977	30977	30920	30920	30920
Humalog	254	254	254	254	274	274	274	274	274	274	274	274
Humira	4118	4338	4344	4338	4338	4338	4338	4338	4338	4338	4338	4338
Humulin	137	136	136	136	138	145	147	146	146	146	146	146
Invokana	392	422	427	427	427	427	427	427	427	427	427	427
Isentress	1370	1381	1381	1381	1381	1381	1381	1381	1381	1379	1379	1379
Januvia	376	380	380	380	380	392	397	397	397	396	396	396
Lantus	373	373	373	373	373	373	373	373	373	384	384	384
Lexapro	300	300	300	300	300	300	300	300	300	300	300	300
Lipitor	251	251	251	251	251	274	274	274	274	274	274	274
Lyrica	377	377	377	376	376	411	412	411	411	411	411	411
Nexium	251	252	252	252	252	252	252	252	252	252	252	252
Novolog	494	494	532	532	532	532	532	532	532	532	532	532
Onfi	909	909	909	993	997	996	996	996	996	996	996	996
Orencia	3786	3790	3790	3786	3786	3777	3786	3786	3786	3781	3777	3777
Otezla	2764	2788	2788	2971	2978	2978	2978	2978	2978	3106	3118	3118
Premarin	156	156	156	156	156	156	156	156	156	156	156	156
Prezista	1406	1459	1455	1454	1454	1454	1454	1454	1454	1454	1454	1454

©2019 Wineinger NE et al. *JAMA Network Open*

Pulmicort	216	216	216	216	216	216	216	216	216	216	216	216
Renvela	508	508	508	508	508	508	508	507	506	506	505	501
Restasis	463	464	463	463	463	463	464	463	463	463	463	463
Simponi	3759	4034	4104	4094	4084	4084	4094	4094	4084	4084	4084	4094
Stelara	8545	9213	9213	9213	9213	9213	9213	9213	9213	9213	9213	9213
Stribild	2888	2880	2880	2880	2880	2871	3069	3078	3069	3069	3065	3069
Symbicort	308	308	308	308	308	308	308	308	308	308	308	308
Synthroid	34	34	34	34	34	34	34	34	34	34	34	35
Tivicay	1422	1530	1530	1530	1530	1529	1530	1530	1526	1526	1526	1526
Triumeq	2406	2590	2589	2582	2582	2578	2581	2581	2578	2578	2578	2578
Trulicity	626	626	626	626	676	676	676	676	676	676	676	674
Truvada	1461	1546	1561	1558	1558	1557	1557	1558	1557	1557	1556	1557
Viagra	328	328	328	328	328	370	370	370	370	370	370	370
Victoza	748	748	748	747	747	757	806	805	805	805	805	805
Viread	995	1052	1063	1063	1063	1060	1063	1063	1059	1059	1059	1057
Vyvanse	271	271	271	271	271	271	271	270	270	270	270	270
Xarelto	358	365	386	386	386	386	386	386	386	386	386	386
Xeljanz	3758	3767	3758	3758	3758	3758	3758	3758	3758	3757	3757	3757
Zetia	313	314	314	314	314	314	314	314	314	311	312	313

©2019 Wineinger NE et al. *JAMA Network Open*
